# Reduction in Structural Disorder and Functional Complexity in the Thermal Adaptation of Prokaryotes

**DOI:** 10.1371/journal.pone.0012069

**Published:** 2010-08-11

**Authors:** Prasad V. Burra, Lajos Kalmar, Peter Tompa

**Affiliations:** 1 Department of Bioengineering, University of California San Diego, La Jolla, California, United States of America; 2 Institute of Enzymology, Biological Research Center, Hungarian Academy of Sciences, Budapest, Hungary; University College Dublin, Ireland

## Abstract

Genomic correlates of evolutionary adaptation to very low or very high optimal growth temperature (OGT) values have been the subject of many studies. Whereas these provided a protein-structural rationale of the activity and stability of globular proteins/enzymes, the point has been neglected that adaptation to extreme temperatures could also have resulted from an increased use of intrinsically disordered proteins (IDPs), which are resistant to these conditions *in vitro*. Contrary to these expectations, we found a conspicuously low level of structural disorder in bacteria of very high (and very low) OGT values. This paucity of disorder does not reflect phylogenetic relatedness, i.e. it is a result of genuine adaptation to extreme conditions. Because intrinsic disorder correlates with important regulatory functions, we asked how these bacteria could exist without IDPs by studying transcription factors, known to harbor a lot of function-related intrinsic disorder. Hyperthermophiles have much less transcription factors, which have reduced disorder compared to their mesophilic counterparts. On the other hand, we found by systematic categorization of proteins with long disordered regions that there are certain functions, such as translation and ribosome biogenesis that depend on structural disorder even in hyperthermophiles. In all, our observations suggest that adaptation to extreme conditions is achieved by a significant functional simplification, apparent at both the level of the genome and individual genes/proteins.

## Introduction

Life has adapted to extreme conditions from sub-zero temperatures in sea ice of polar regions to boiling temperatures in hydrothermal vents [1], [2]. As temperature dramatically affects all cellular processes, adaptation occurred at many levels, from codon bias through membrane fluidity to protein stability and enzyme activity [3], [4]. This latter, i.e. the adaptation of the catalytic, structural and regulatory functions of proteins to extreme conditions, is of particular interest from both theoretical and practical points of view. The underlying molecular mechanisms have been studied either by comparing the structures of proteins isolated from organisms that thrive at low (psychrophilic), moderate (mesophilic) or high (thermophilic) temperatures [5], [6], [7], [8], or analyzing sequences of the respective genomes/proteomes [9], [10], [11], [12]. It appears that proteins of vastly different optimal temperatures show only subtle differences in structure, and their adaptation relies on an interplay of various factors affecting stability, such as hydrophobicity, H-bonds, structural cavities, ion-pairs, and secondary structural elements, including surface loops [13]. These differences correspond to a characteristic amino acid bias, denoted as charge vs. polar bias, in thermophiles [5], [10]. Genome-level studies suggest that the optimal growth temperature (OGT) of the organism correlates best with the total fraction of amino acids Ile, Val, Tyr, Trp, Arg, Glu and Leu in the proteome in the wide range -10°C to 110°C [12]. Compositional differences contribute to thermal adaptation through fine-tuning stability, flexibility and specific activity of proteins [6], by making them in general more rigid and more stable to thermal unfolding with increasing growth temperatures.

Structural comparisons, however, have been limited to those proteins that have well-defined 3-dimensional structures, the analysis of which provided structural details down to the atomic-level. The recent recognition of intrinsically disordered proteins/regions (IDPs/IDRs), however, complicates this simple picture, and it may shed new light on adaptation to extreme environmental conditions. Unlike globular proteins, IDPs/IDRs lack well-defined 3D-structures in their native state [14], [15], [16], yet they constitute a significant fraction of proteomes, with an increased level in eukaryotes compared to prokaryotes [17], [18], [19]. Long IDRs often have essential functions in bacterial proteins, such as in the case of fibronectin-binding protein A, FnbpA [20] and prokaryotic ubiquitin-like protein, PuP [21]. IDPs/IDRs have a biased amino acid composition, depleted in order-promoting (Trp, Cys, Phe, Ile, Tyr, Val, and Leu) and enriched in disorder-promoting (Ala, Arg, Gly, Gln, Ser, Pro, Glu, and Lys) amino acids [22], [23]. Disordered proteins carry out essential functions mostly associated with signal transduction and transcription regulation [24], [25] in eukaryotes, and also in prokaryotes, as reported in the case of FlgM anti-sigma factor [26], and CcdA antitoxin [27], for example. IDPs are often resistant to boiling temperatures, as witnessed by their usual purification procedure via heat-treatment [14], [28], also applied in their proteomic identification [29], [30]. IDPs are also cold-resistant, as inferred from the involvement of some disordered plant dehydrins in the response to water stress elicited by freezing temperatures [31], [32], also underlined by direct experimental evidence [33].

These features suggest that the increased use of IDPs could contribute to the general evolutionary strategy of thermal adaptation, a feature so far completely neglected in respective studies. In prior analyses, point mutations [5], [6], [7], [8] or deletion of surface loops [13] have been suggested to bring about increased thermal stability concomitant to decreased flexibility. The point, however, has been missed that disordered regions are often not part of ordered structures and they follow a different functional/evolutionary logic. This distinction enables adaptation to proceed by changes of the opposite sign in ordered and disordered proteins, such as a reduction of flexibility of globular proteins by an increase in hydrophobicty and a parallel increase in structural disorder/frequency of IDPs due to a decrease in hydrophobicity. *In vitro*, signs of this dual logic can be witnessed by an increase of thermal stability of proteins by deleting flexible loops that would serve to initiate unfolding [13], but also by fusing disordered terminal appendages, which ablate irreversible aggregation [34], [35].

The data available from systematic studies [36] of the OGT of a large number of bacteria enables us to probe the above inference through bioinformatics analyses. Full genome sequences and actual growth temperatures of about 300 prokaryotes, psychrophiles (OGT: 5–17°C), mesophiles (20–42°C), thermophiles (45–75°C) and hyperthermophiles (75–105°C) can be found in the NCBI Genome Project database. We predicted their disorder by the IUPred [37], [38] and VSL2 [39] algorithms and correlated it with OGT. Unexpectedly, the average disorder is very low in all psychrophilic and hypertheromphilic organisms (2–5%), but it varies a lot in mesophilic and thermophilic organisms, reaching very high levels (25%) in certain thermophiles. By observing a general reduction in genome size and in the number and disorder of transcription factors, we suggest that adaptation to extreme temperatures has occurred via a reduction in functional complexity favoring metabolism at the expense of regulation. Overall, these findings suggest that cold- and heat-resistance of IDPs has not been exploited for evolutionary adaptation to extreme temperatures probably because their functions are mostly compatible with ambient temperatures only.

## Results

### Disorder in bacterial genomes

**Figure 1:**
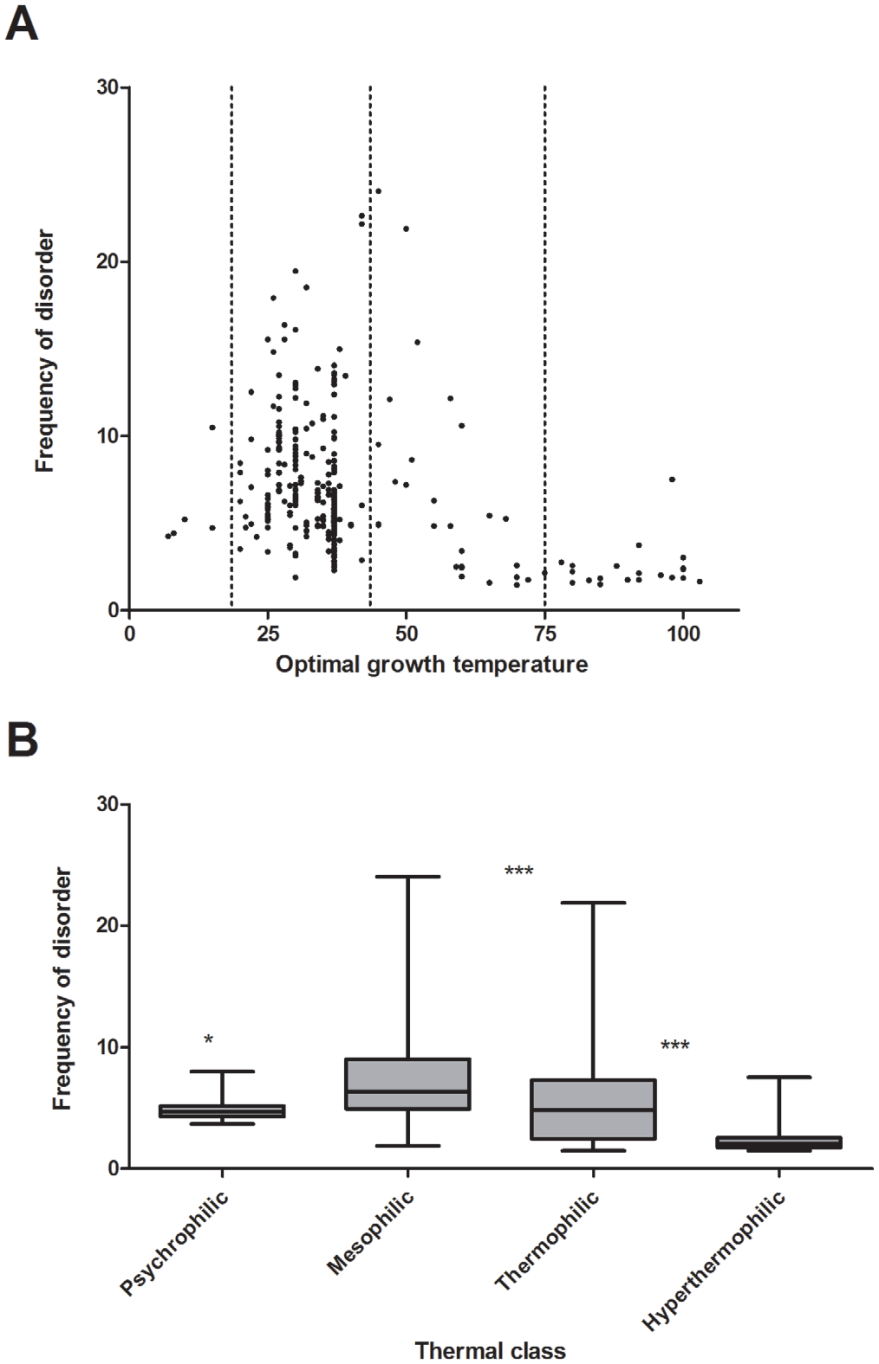
Structural disorder and optimal growth temperature of prokaryotes. (A) Average structural disorder of proteins in prokaryotes was predicted by the IUPred algorithm [37], [38], averaged over all proteins in the proteome, and is shown as a function of the OGT of the bacteria (borders of OGT classes marked by vertical dashed lines). (B) Because the exact value or range of OGT is not reported for all prokaryotes, which, however, are classified as psychrophiles (OGT 5–17°C), mesophiles (20–42°C), thermophiles (45–75°C) and hyperthermophiles (75–105°C), average disorder within these groups has also been calculated. The horizontal line shows median of disorder, whereas the grey box represent standard error of mean (SEM). Error bars show the highest and lowest value observed within that group. Asterisks mark if difference of average disorder of the given group and mesophiles is significant (one asterisk: significant, p<0.05, three asterisks: highly significant, p<0.0001).

Structural disorder in prokaryotic genomes was predicted by the IUPred [37], [38] algorithm, and various measures, such as average disorder score, percent of disordered residues in proteins, percent of proteins with average disorder score above 0.5, percent of proteins with more disordered than ordered amino acids (mostly disordered proteins) and disorder in genomes were calculated (Table S1). To demonstrate that prediction of disorder is not biased by the skewed amino acid composition of extremophiles [12], we have repeated predictions with PONDR VSL2 [39], and have also carried out a very simple disorder-prediction approach that depends only on gross amino-acid composition measures (Charge-Hydropathy (CH) plot or Uversky plot [22]). Neither amino-acid composition, nor distribution of proteins in the CH-plot (Supplementary Figure S1) show a characteristic bias between the four groups, which suggests that disorder predictions by IUPred truly reflect the structural status of proteins encoded by genomes of bacteria of various OGT values (cf. Figure 1).

Average disorder of proteins (Figure 1A) and other measures of structural disorder (Table S1) in mesophiles and thermophiles varies a lot and reaches high levels in certain genomes. Hyperthermophiles, on the other hand, invariably show a low level of disorder, clustering on the lower edge of the apparently acceptable range of disorder characteristic of bacteria (above 1.5%) with the exception of one methanogen (Methanopyrus kandleri), which has 7.51% predicted disorder at an OGT of 98°C, probably reflecting the general positive deviation of disorder in methanogenes. The lifestyle of psychrophiles also appears to be compatible with only a low level of disorder. In all, bacteria with low levels of disorder are found throughout the entire OGT range, whereas the maximum of the frequency of disorder as a function of temperature shows a rather normal distribution that peaks between 40°C and 50°C.

Because several bacteria are noted for their habitat, without an exact OGT value determined, we also compared characteristic structural disorder in different temperature categories. A significant decrease of average disorder content in all non-mesophilic groups compared to mesophiles using nonparametric t-test is seen (Figure 1B). The structural and functional significance of this finding is underscored by a similar dependence on OGT of disorder found in long IDRs and mostly disordered proteins (Supplementary material, Figure S2). IUPred and VSL2 predicted a similar dependence, albeit somewhat different actual values. This distribution is unexpected, given the noted cold-resistance and heat-resistance of IDPs. We next examined possible explanations for this behavior.

### Disorder in different taxons versus disorder in bacteria of different lifestyles

A possible explanation of the observed behavior is that psychrophilic and hyperthermophilic prokaryotes are evolutionarily related to mesophiles of low disorder, whereas relatives of mesophilic prokaryotes of high disorder have not penetrated habitats of extreme temperatures. This is possible because often differences observed are not central to the process of adaptation, only represent side-effects [40]. If this were true, the lack of prokaryotes with a high level of disorder among hyperthermophiles would not reflect a selection against structural disorder driven by adaptation to high temperatures, rather it a random drift or selection for other features more related to phylogenetic relationships [40].

**Figure 2:**
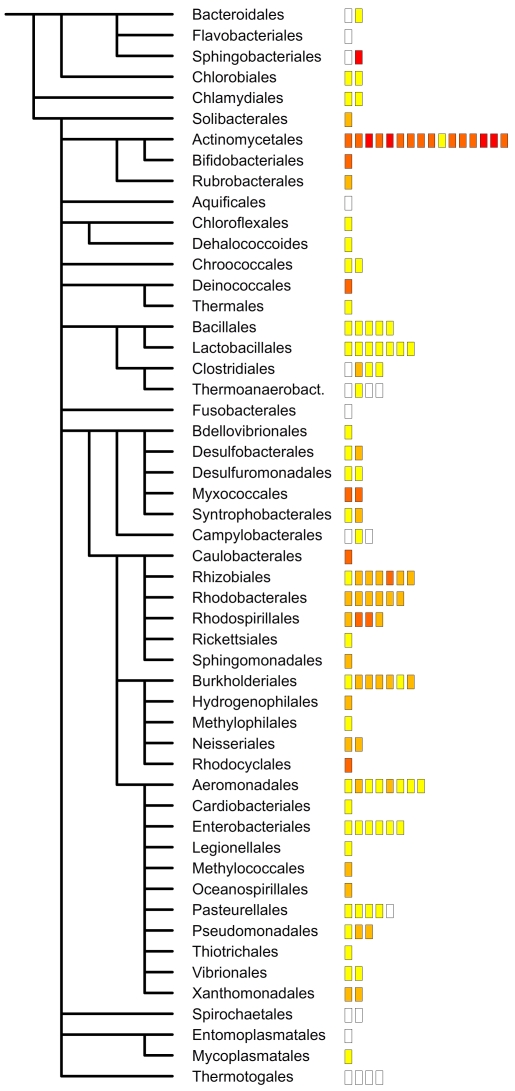
Taxonomic distribution of structural disorder in bacteria. Average structural disorder in bacteria in different odrers has been calculated and is shown by color coding. Orders are given by name, and genera within are colored by boxes that reflect the respective average level of disorder, such as white (0–16%), yellow (16–20%), ochre (20–24%), orange (24–28%) and red (above 28%). Generally, bacteria that belong to the same genus tend to have similar average disorder, but no general correlation between closely related orders is apparent.

To probe this possibility, we have checked if predicted disorder reflects taxonomic relatedness more than optimal habitat of bacteria. To this end, predicted disorder (Table S1) was plotted on the phylogenetic tree of bacteria (Figure 2). The figure shows that except for a few cases (e.g. Actinobacteria) structural disorder correlates with the OGT rather than the taxonomical position of the species, which suggests that low levels in hyperthermophiles and psychrophiles is the result of evolutionary selection process. In principle, it is conceivable by either removal of proteins with a higher-than-average disorder or an overall diminution of disorder in all proteins, or both.

### Thermal adaptation and functional complexity

**Figure 3:**
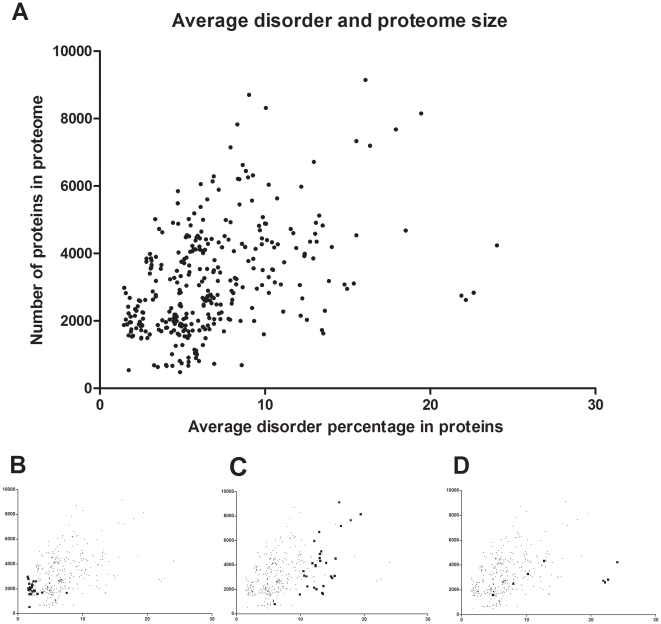
Average disorder of proteins and genome size. (A) The size of proteome (actually the number of annotated genes in the genome) is shown as a function of average predicted disorder of proteins in prokaryotes with known status of thermal adaptation. Particular groups are also shown highlighted, such as hyperthermophiles (B), Actinobacteria (C) and halophiles (D).

The general diminution in the frequency of structural disorder raises a very important issue with respect to how prokaryotes of low and high OGTs live without – or find substitutes for - the functions these proteins fulfill in mesophiles and thermophiles. Because structural disorder is strongly correlated with regulatory functions [19], [25], [41], a significant reduction of disorder upon thermal adaptation may correspond to the reduction of functional complexity of a species. Because the usual measure of complexity of different cell (or tissue) types cannot be applied to bacteria, we may intuitively relate complexity here with the number of genes and their encoded disorder. This is justified by observations that i) disordered proteins/regions in general are implicated in functions related to complexity, such as signaling and transcription regulation [24], [25]; ii) structural disorder correlates with complexity at the level of whole genomes, as underlined by the observation that the frequency of disorder increases with increasing complexity of the organism, with a particularly conspicuous increase in evolution between prokaryotes and eukaryotes [23]; iii) there is a direct link between complexity and disorder in transcription regulation [42], and iv) there is a significant difference between free-living bacteria, such as Actinobacteria of very complex responses and obligatory parasites, such as Mycoplasma, which are functionally “simple” because they live in a constant environment and cannot respond to many changes. Thus, we reasoned that functional simplification may also be apparent at the level of the whole genome/proteome in the thermal adaptation of bacteria, as already suggested based on observing the correlation of simple sequences of proteins and genome size [43]. Because simple sequences are related to structural disorder, we correlated the proteome size (number of proteins) with average protein disorder (Figure 3A). Clearly, proteome size is correlated with average structural disorder, and hyperthermophiles are located in the lower left corner of the plot, with small genomes and low average disorder (Figure 3B). This correlation between proteome size and average disorder applies to all bacteria, with some clear outliers, such as Actinobacteria (Figure 3C), which have a high predicted disorder at varying genome sizes, and halophilic bacteria (Figure 3D), which have small genomes but a high disorder. While high predicted disorder in Actinobacteria can be explained with their high complexity, we presume that disorder is mispredicted in prokaryotes adapted to high saline concentration because of the high surface charge of their globular proteins [44]. Overall, this correlation shows a reduction in genome size also previously observed in obligatory symbionts and parasites [45], which leaves only proteins with lower-than-average disorder.

### Thermal adaptation in transcription factors

These foregoing results suggest that the observed low disorder in hyperthermophiles reflects genuine adaptation at the level of genomes and/or individual proteins. Such an adaptation raises a very serious question with respect to the regulatory functions carried out by IDPs/IDRs in mesophiles: either these functions have been lost or simplified in prokaryotes of low/high OGT, or they have been substituted by ordered proteins/regions. We thought to answer this question by studying transcription factors (TFs), because they represent a prominent and indispensable functional group with a high level of functionally important disorder in both prokaryotes and eukaryotes [25], [46], [47], life in general cannot exist without them and their disorder is correlated with the number of genes they regulate, which suggests that their disorder is directly linked with functional complexity of the organism [42], [45]. Their function-related disorder is most apparent in trans-activation, but also in DNA-binding [46], [47], as also raised in the classic paper on the link between flexibility and specificity in DNA binding [48]. The function of long IDRs in several prokaryotic transcription-regulatory proteins, such as FlgM anti-sigma factor [26], plasmid partition protein KorB ([49], small DNA binding protein H-NS [50] and CcdA antitoxin [27], for has been directly established.

**Figure 4:**
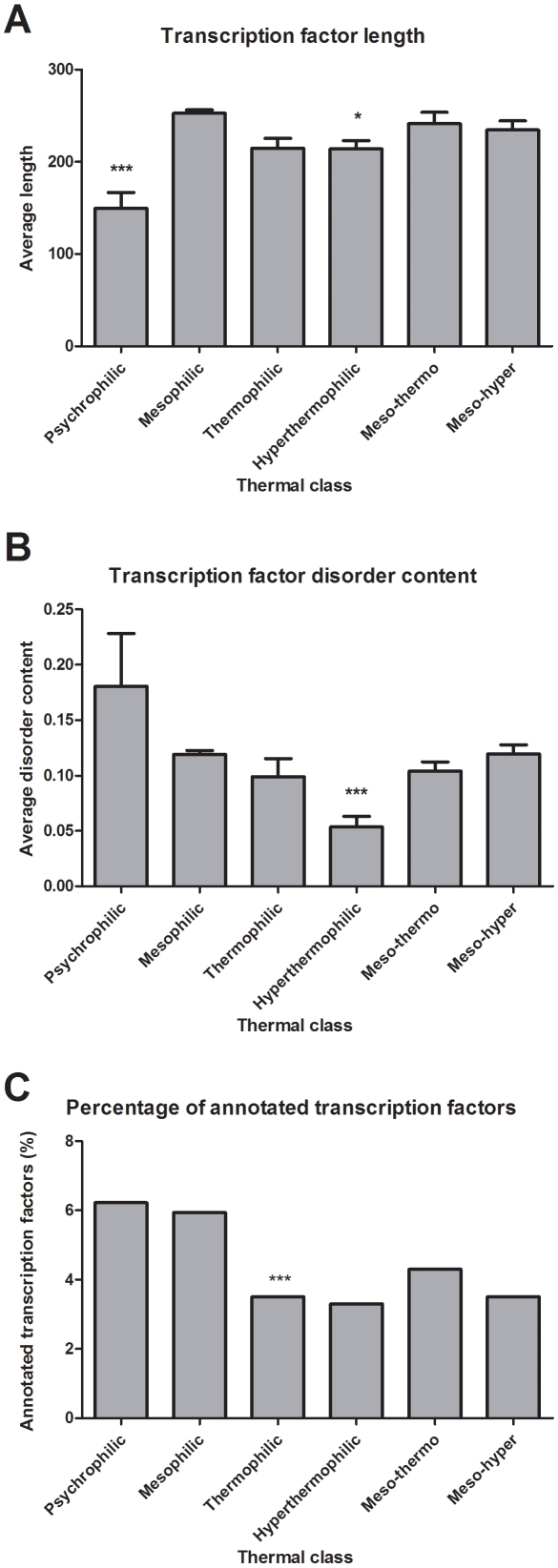
Transcription factors in the thermal adaptation of prokaryotes. It is assessed how transcription factors, i.e. a group of proteins of an essential function that depends on structural disorder is affected by thermal adaptation. (A) The average length of annotated transcription factors (error bars, SEM) is shown for the four groups psychrophiles, mesophiles, thermophiles and hyperthermophiles. The average length of TFs in mesophiles with the same average proteome size as thermophiles (meso-tehrmo) or hyperthermophiles (meso-hyper), is also shown. (B) The average level of predicted disorder of annotated transcription factors (error bars, SEM) for the four groups. The average disorder of groups meso-tehrmo and meso-hyper, as defined above, is also shown. (C) The ratio of TFs among all annotated genes is shown for the four groups and meso-thermo and meso-hyper, as defined above. In all three panels, asterisks mark if difference of average from that of mesophiles is significant (one asterisk: significant, p<0.05, three asterisks: highly significant, p<0.0001)

We used the GO annotation (GO:0003700) to filter out TFs from the high-quality SwissProt database in the four OGT groups and the two mesophilic control groups with the same proteome size as thermophiles (meso-thermo) and hyperthermophiles (meso-hyper) as defined above. As it was previously reported [46], the length of TFs is reduced in prokaryotes compared to eukaryotes, so first we checked if the average length of TFs in psychrophiles and hyperthermophiles is different from that in mesophiles. We found that TFs in both groups are significantly shorter (Figure 4A), but the difference between thermophiles and mesophiles is not significant. The difference between hyperthermophiles and their proteome-size-matched mesophilic controls (meso-hyper) was not significant (Figure 4A). On the other hand, the average predicted disorder content of TFs in hyperthermophiles is significantly decreased (P<0.0001), compared to either mesophiles or the meso-hyper controls (Figure 4B).

These observations are compatible with a general shortening of TFs at the expense of IDRs in adaptation to extremely high temperatures, but they also allow some more drastic changes removing the most highly disordered TFs upon adapting to high-temperature habitats. To check whether this latter has taken place, we assessed if the frequency of TFs has been lowered in hyperthermophiles vs. mesophiles. In doing this, we noted a possible source of error because the ratio of annotated genes is lower in hyperthermophiles than in mesophiles. Thus, by complementing the Swiss-Prot dataset with TrEMBL, we checked the frequency of TFs in all annotated proteins in the four thermal groups (Figure 4C). There is a lower number of TFs in thermophiles than in the thermo-meso group, but not so in the hyperthermophiles vs. the meso-hyper group. This suggests that the number of TFs correlates with the genome size, but structural disorder is under separate selection pressure, not directly linked with the number of TFs.

These observations suggest that hyperthermophiles reduce the level of disorder of their TFs, i.e. even if they find ordered substitutes for some disordered TFs, they experience a significant reduction of functional complexity that primarily affects regulatory functions.

### Residual protein disorder in hyperthermophiles

While the frequency of protein disorder in hyperthermophiles is extremely low, it should be noted that there is a residual predicted disorder throughout the entire OGT range, i.e. life appears to be incompatible with less than about 1.5% disorder (cf. Figure 1A and Figure 3A). Given the major reduction of disorder in TFs, it is possible that there are certain functions which depend even more on disorder that account for this residual disorder. On the contrary, if this low disorder content is distributed with the same pattern among functional groups in hyperthermophiles as in mesophiles, it would rather suggest a noise, i.e. that disorder-related functions can be generally disposed of or substituted by ordered proteins in hyperthermophiles.

Thus, we filtered out proteins with long IDRs, which are likely to mark specific disorder-related functions, and categorized them by their GO biological process annotation. Hyperthermophiles were compared to two mesophilic group, one with low average disorder content (MLD, 1–4%, comparable to that in hyperthermophiles), and the other with higher disorder content (MMD, 8–11%). We reasoned that a comparison with the MLD group reveals the signs of adaptation to high temperatures, not obscured by the effect of reduction in genome size. In accord, we observed that the residual disorder is concentrated in hyperthermophiles in a few functions (Table 1). Most significantly, about 35% of proteins with long IDRs are associated with translation, many of them associated with ribosomal functions. Proteins annotated to transport process (e.g. protein translocases), regulation of transcription and ribosome biogenesis are also significantly overrepresented in hyperthermophiles.

**Table 1:** GO classification of proteins with long disordered region.

GO cellular process annotation	H	MLD	MMD
translation	34,94	12,10	4,84
transport	11,81	4,52	5,52
regulation of transcription, DNA-dependent	5,06	2,15	10,60
chemotaxis	3,86	7,01	5,03
metabolic process	3,13	2,19	5,09
translational elongation	3,13	<1%	<1%
oxidation reduction	2,41	1,80	7,11
ribosome biogenesis	2,41	<1%	<1%
signal transduction	2,41	5,57	4,48
proteolysis	1,93	6,88	6,57
two-component signal transduction system	1,93	1,36	4,77
peptidyl-histidine phosphorylation	1,20	1,14	3,07
cell adhesion	<1%	4,12	<1%
pathogenesis	<1%	2,41	<1%
protein secretion	<1%	1,36	3,22
transcription	<1%	<1%	4,78

The percent of proteins with at least one long IDR (≥30 consecutive residues) in hyperthermophiles (H), mesophiles with a low level of average disorder (1–4%, group MLD) and mesophiles with a medium level of average disorder (8–11%, group MMD).

## Discussion

The predicted disorder in prokaryotes of various OGTs shows an unexpected distribution. Because IDPs often do not aggregate under high- or low-temperature conditions [28], [33], and they can be effective in preventing other proteins from aggregation [31], [32], [34], [35], it was expected that prokaryotes adapted to extremely low (psychrophiles) or extremely high (hyperthermophiles) temperatures have relied on IDPs in their adaptation to these extreme temperatures. The reality of this expectation is probably underscored by a high average disorder in certain thermophiles, with the highest levels found in bacteria with OGTs around 40–50°C. Apparently, these species take advantage of the increased thermal stability of IDPs and the functional advantages they confer. Above these temperatures, however, this is not the case, i.e. bacteria living at very high temperatures have the lowest levels of disorder.

A caveat to this unexpected observation is that prediction of structural disorder in proteins that function at extreme conditions carry a potential element of error. Because disorder predictors have been trained mostly on data deposited in the DisProt database, dominated by mesophilic eukaryotic proteins [51], they may underestimate disorder in hyperthermophilic (and psychrophylic) proteins. There are two points against this objection. First, we have applied two predictors, which rely on different principles. VSL2 has been separately optimized on short- and long disordered sequences [39], whereas IUPred has not actually been trained on IDP sequences, but developed to estimate the total pairwise interresidue energies of sequences [37], [38]. Second, we have calculated the amino acid composition of proteins in all the genomes and plotted them on a CH plot suggested by Uversky [22] to demonstrate that possible differences in amino acid composition do not introduce an element of bias into our predictions. Both these approaches lend credence to our conclusion with respect to the paucity of structural disorder in extremophiles.

This unexpected behavior may have two different explanations. On the one hand, it is conceivable that low disorder is not an adaptive trait in thermal adaptation, only a side effect resulting from neutral drift or adaptation to other selective pressures [40], or from evolutionary descent from mesophiles with low disorder. On the other hand, it is possible that diminution of structural disorder in the course of adaptation to higher temperatures is a genuine adaptive trait. There are several points against the first explanation. The taxonomic distribution of hyperthermophilic behavior and disorder suggests that bacteria that thrive at high OGTs can be found in many taxons. Thus, adaptation to extreme temperatures has occurred in many lineages and has been accompanied by a reduction in genome complexity and protein disorder. This scenario is in full agreement with previous observations that adaptation to high temperatures is a fast process on an evolutionary timescale that could occur several times within a single lineage, resulting in a practically random distribution of hyperthermophiles on the phylogenetic tree [4]. A comparison of different control groups corroborates this conclusion. Structural disorder of TFs is highly significantly different from that of mesophilic/thermophilic TFs, much more so than their lengths. The difference from mesophilic-hyperthermophilic genome-matched controls is also significant, suggesting adaptive forces beyond random noise or mere consequence of genome reduction. Further, TFs in psychrophiles are very significantly shorter, but tend to be more disordered, than those in hyperthermophiles, even though both groups are reduced in genome size. In addition, the number of TFs is not significantly lower in hyperthermophiles than in hyper-meso controls with the same genome size, whereas their disorder is significantly reduced. In all, these observations argue convincingly that a reduction in structural disorder is not a side effect but causatively linked with thermal adaptation.

Thus, a significant reduction of structural disorder in bacteria living at very high (and very low) temperatures is central to the process of thermal adaptation. This adaptive change might have taken place either by losing functional disordered proteins (thus existing without the functions they carry out in mesophiles) or gradually reducing their disorder content by replacing their IDPs/IDRs with ordered functional analogues. Our observations argue for the first mechanism, i.e. a significant functional reduction in hyperthermophiles. First, their genome size is significantly reduced, which suggests a reduction of complexity as a means of adaptation. Second, the comparison of transcription factors, the function of which is indispensable for life, also argues in favor of this observation. TFs are significantly shorter, and have a reduced disorder in hyperthermophiles in a way reminiscent of the situation in prokaryotes as a group in comparison to eukaryotes [46], [47], where shorter and less disordered TFs mark the diminution in regulatory functions, i.e. functional complexity. A similar conclusion has been made by observing a correlation of the number of TFs and genome size in prokaryotes, except for obligatory symbionts and parasites, which have very low numbers and apparently have given up a good deal of their regulatory functions [45]. Although emerging ordered proteins/regions in principle might have taken over these functions, we also observed that hyperthermophilic TFs are less disordered than TFs from mesophiles with a similarly compact genome, which also supports that besides simplification manifested in genome reduction, a functional simplification at the level of proteins has also taken place. In addition, the ratio of TFs among annotated genes is reduced in hyperthermophiles, also arguing against the replacement by novel – more ordered – TFs.

In terms of the evolutionary logic of this change, however, it is still open if reduction in structural disorder is only a consequence of reduction of functional complexity, or rather a driving force of the adaptation of the organism. In a way this is a semantic question, because there is many evidence in the literature that structural disorder and complexity are correlated, both at the level of individual proteins, where IDP functions correlate with signaling and regulation, and whole genomes, where the frequency of disorder increases with increasing complexity of the organism [24], [25], [41], [52]. Thus, evolutionary changes (point mutations, deletions of regions, silencing of genes, etc…) that reduce disorder will tend to strip the organism of functions that increase its complexity, and leave functions that are required for its basic, non-regulated existence. In this sense, reduction in disorder is not a side-effect of selection for reduced complexity, rather the mechanism of this evolutionary drive.

In light of the possible advantages that would result from the heat-resistance of IDPs, their reduction suggests that their functions are incompatible with elevated temperatures (and probably also with low temperatures, to which there is very little data, though). IDPs carry out their functions by two different mechanisms, as entropic chains and by molecular recognition [14], [15]. Entropic chain functions result from the ability of the polypeptide chain to rapidly fluctuate between many alternative conformations, which result in functions such as linkers, spacers, bristles or springs; these functions can be principally fulfilled at elevated temperatures and they might even be operative at low temperatures, where adaptation even of globular proteins (enzymes) is thought to have occurred by way of an increase in flexibility and proportion of flexible loops [5], [6], [7], [8]. IDPs that function by molecular recognition, on the other hand, usually bind their partner via short recognition elements termed preformed structural elements, PSEs [53], molecular recognition features, MoRFs [54] or short linear motifs, SLiMs [55]. These short motifs undergo induced folding upon partner binding from an initially disordered state [56] and usually engage in weak and transient, yet specific interaction with the partner [57], [58]. The result of such binding is the modification of the activity of the partner, the assembly of a complex or local posttranslational modification of the IDP [14], [15]. These short motifs arise by evolutionary convergence, i.e. by random mutations and functional selection, rather than duplication and subsequent divergent spread in the genome, such as in the case of binding domains [59]. Probably it is this double constraint set by thermodynamic fine-tuning and evolutionary adaptability that precludes the widespread use of this functional mode in extremophiles. At high temperatures, it is probably too weak binding that makes short motifs embedded in disordered regions non-functional. At low temperatures, entropic chain linkers may have a significant advantage, as related to the significantly higher flexibility of ordered enzymes, which can thus function under conditions where significant activation energy is difficult to obtain. Short binding motifs, however, may bind too weak, because they primarily rely on hydrophobic interactions [56], [60]. As observed with respect to the increase in flexibility in the catalytic function of psychropilic enzymes, a reduced efficacy of the hydrophobic interactions [61] may have a functional advantage, whereas in the case of short IDP binding motifs it may curtail the functional advantages they provide in mesophiles.

Whereas this scenario applies to TFs, there appears to be a few functions that cannot exist without an appreciable level of disorder even in hyperthermophiles. Proteins involved in translation, transport, regulation of transcription and ribosome biogenesis have a much higher level of disorder in hyperthermophiles than in mesophiles or even in mesophiles with the same genome size as hyperthermophiles. In light of the foregoing arguments, it is not clear how these proteins function at high temperatures, but it is possible that they do not engage in weak binding by short motifs but undergo induced folding of extended regions resulting in much stronger binding, as observed in the assembly of translation initiation [62] or the ribosome [63]. Such extended disordered binding regions have been observed in the case of disordered domains [59], representing a third type of molecular recognition entity besides ordered domains and disordered short motifs.

In conclusion, our data point to a significant reduction in structural disorder accompanied by reduction in genome size in adaptation to habitats of very high (and very low) temperatures, with a concomitant diminution in functional complexity. Apparently, the price an organism pays for the ability to exist under extreme conditions is a reduction in adaptability and responsiveness to environmental changes.

## Methods

### Genome sequences

Genome sequences of 332 prokaryotes with known temperature (or temperature range) for optimal growth were downloaded from the NCBI Genome Project database (Supplementary material, Table S1). In terms of their OGTs, prokaryotes are classified into four groups as psychrophiles (OGT: 5–17°C), mesophiles (20–42°C), thermophiles (45–75°C) and hyperthermophiles (75–105°C), as suggested in the NCBI database. If exact OGT is not specified, we searched the PGTdb [36] for temperature range. Of the 332 cases, exact OGT is given in 195 cases, whereas a respective temperature range (e.g. 20–30°C, cf. Table S1) in 124 cases. In these latter cases, the average of the range was taken as the OGT characteristic of that species. In the remaining 13 cases, no value or range of OGT is reported, but the organism is clearly classified to belong to one of the above four categories.

### Disorder prediction

Structural disorder of proteins was predicted by two predictors, IUPred [37], [38] available at http://iupred.enzim.hu/ and PONDR VSL2 [39] available at http://www.ist.temple.edu/disprot/Predictors.html. A residue was classified as locally disordered if its score was above the threshold of 0.5. From the pattern of disorder of proteins, various measures were calculated, such as the average disorder score of proteins, the percentage of disordered residues in the whole proteome, and the percentage of proteins with more than 50% of their residues disordered (mostly disordered proteins). The frequency of residues in long IDRs (≥30 consecutive residues predicted as disordered), which is generally thought of as functionally important, was also calculated [23].

### Amino acid composition and Charge-Hydropathy (CH) plot

The amino acid composition of proteins in the four thermal categories were extracted from a non-redundant SwissProt dataset by analyzing all proteins from the studied species. CH values were calculated as described by Uversky et al. [22] on 2000 randomly selected proteins from a non-redundant SwissProt dataset in each thermal category. The CH plot is divided into two regions by a line (equation H = (R+1.151)/2.785, R: mean net charge, H: mean hydrophobicity) which best separates disordered (left side) and ordered (right side) proteins. In the calculation, a normalized Kyte-Doolittle scale was used to obtain hydropathy values, while Arg, Lys, Glu and Asp residues were considered in calculating mean net charge values.

### Evolutionary relatedness

Evolutionary relatedness of prokaryotes in terms of disorder was asked by looking whether the level of predicted structural disorder shows characteristic taxonomical distribution, or rather, a correlation with lifestyle. To this end, species of bacteria and archea were categorized according to their taxonomic classification (order and genera within, source: UniProt).

### Frequency, length and disorder of transcription factors in prokaryotes

We asked if a functionally indispensable and usually highly disordered [46], [47] group of proteins, transcription factors, were differentially represented in prokaryotes of various OGTs. To this end, transcription factors in the four groups of bacteria and archea were selected by Gene Ontology (GO) annotation from UniProt SwissProt database. The search resulted in 18 transcription factors in psychrophiles, 1581 in mesophiles, 62 in thermophiles and 101 in hyperthermophiles (Supplementary material, Table S2). For comparisons of length and disorder content, we also created two subsets from mesophiles, with the same average proteome size as thermophiles (meso-thermo) and hyperthermophiles (meso-hyper), respectively. These datasets enabled us to address whether the reduction of disorder in TFs is a result of genome reduction or structural-functional alteration. For each group, the average length was calculated and the frequency of structural disorder was predicted by IUPred and VSL2.

### Functional categorization of proteins

To check for functional correlations, we categorized the proteins containing at least one long IDR (≥30 consecutive disordered residues) by their GO cellular process annotations. We then looked for the prevalence of distinct functional categories in three groups of prokaryotes, hyperthermophiles, mesophiles with a low level of average disorder (1–4%, group MLD) and mesophiles with a medium level of average disorder (8–11%, MMD).

### Statistical analysis and programming

We used the Mann Whitney test and Chi-square analysis with a 95% confidence interval to evaluate the significance of differences between selected groups. All programs were written in BOS(v3.0) [64] – an integrative biological programming environment - (http://www.biobhasha.org) and Perl language. BOS and Perl scripts and other compiled software (e.g., IUPred, etc.) were executed locally.

## Supporting Information

Figure S1 Charge-Hydropathy (Uversky-) plots [22] and amino acid composition of proteins in the four thermal categories. The Charge-Hydropathy plots of proteins from psychrophiles (A), mesophiles (B), thermophiles (C) and hyperthermophiles (D) have been generated as described in Data and analysis. The red line corresponding to the equation H = (R+1.151)/2.785 (R: mean net charge, H: mean hydrophobicity) indicates the border between disordered (left side) and ordered (right side) proteins. No characteristic difference between the pattern of proteins can be observed in the different thermal group. Amino acid composition of all proteins from the studied prokaryotes (E) is also plotted.
(2.11 MB TIF)

Figure S2 Distribution of various measures of structural disorder as a function of OGT of prokaryotes. (A) Percentage of mostly disordered proteins (more than 50 percent of residues in a protein are disordered), (B) frequency of residues in long IDRs (at least 30 consecutive residues predicted as disordered), (C) total average of disorder scores in whole proteome, in the function of OGT.
(0.83 MB TIF)

Table S1 Prokaryote species included in the analysis.
(0.10 MB PDF)

Table S2 Annotated TFs in Swiss-Prot in the four OGT groups psychrophiles, mesophiles, thermophiles and hyperthermophiles.
(0.21 MB PDF)
