# Supplementary figures and images for: Reduction in Structural Disorder and Functional Complexity in the Thermal Adaptation of Prokaryotes
Source: PLoS One. 2010 Aug 11;5(8):e12069. doi: 10.1371/journal.pone.0012069 (PMC2920320; doi:10.1371/journal.pone.0012069)

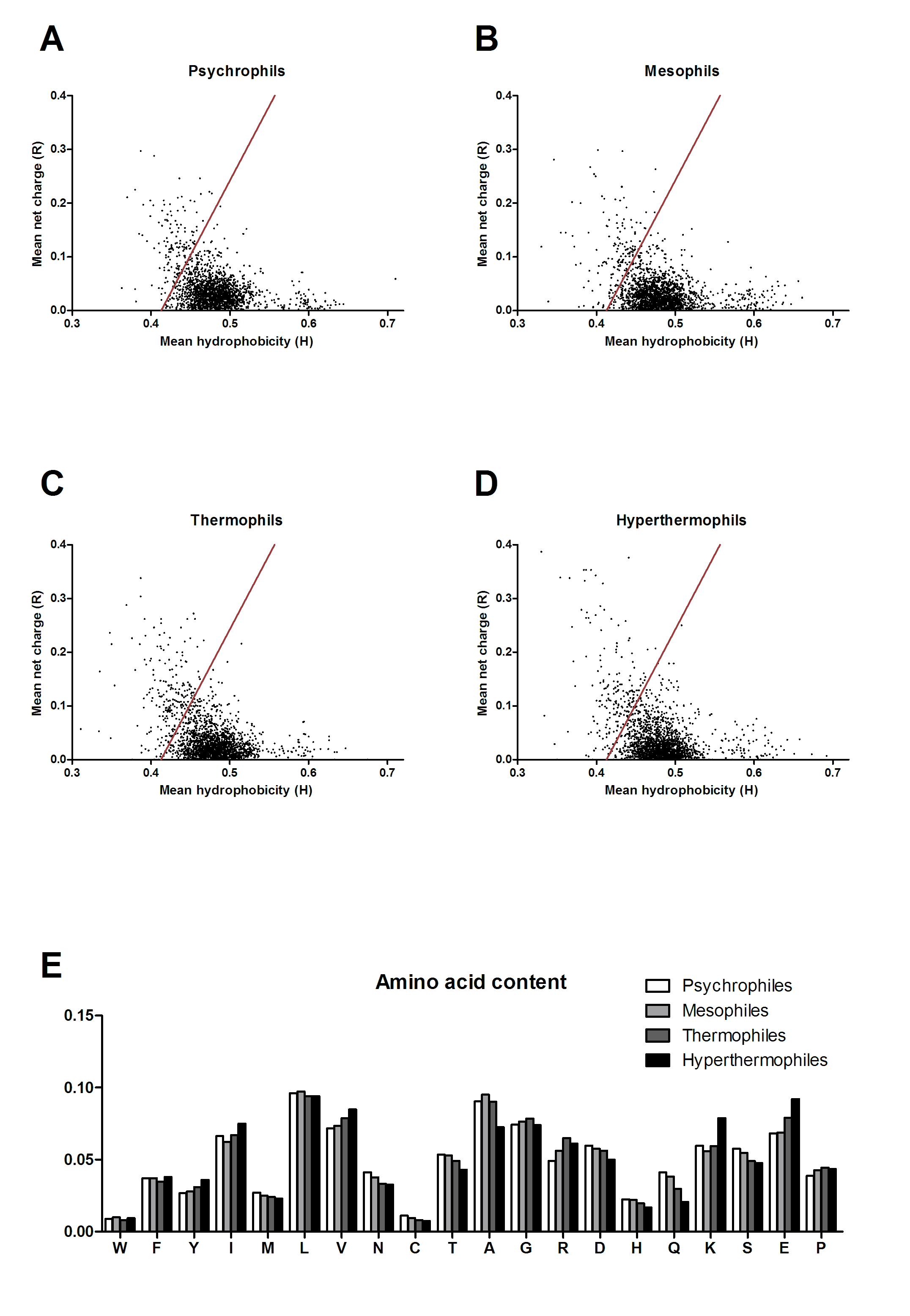

Supplement: Figure S1 — Charge-Hydropathy (Uversky-) plots [22] and amino acid composition of proteins in the four thermal categories. The Charge-Hydropathy plots of proteins from psychrophiles (A), mesophiles (B), thermophiles (C) and hyperthermophiles (D) have been generated as described in Data and analysis. The red line corresponding to the equation H = (R+1.151)/2.785 (R: mean net charge, H: mean hydrophobicity) indicates the border between disordered (left side) and ordered (right side) proteins. No characteristic difference between the pattern of proteins can be observed in the different thermal group. Amino acid composition of all proteins from the studied prokaryotes (E) is also plotted. (2.11 MB TIF) [file pone.0012069.s001.tif]

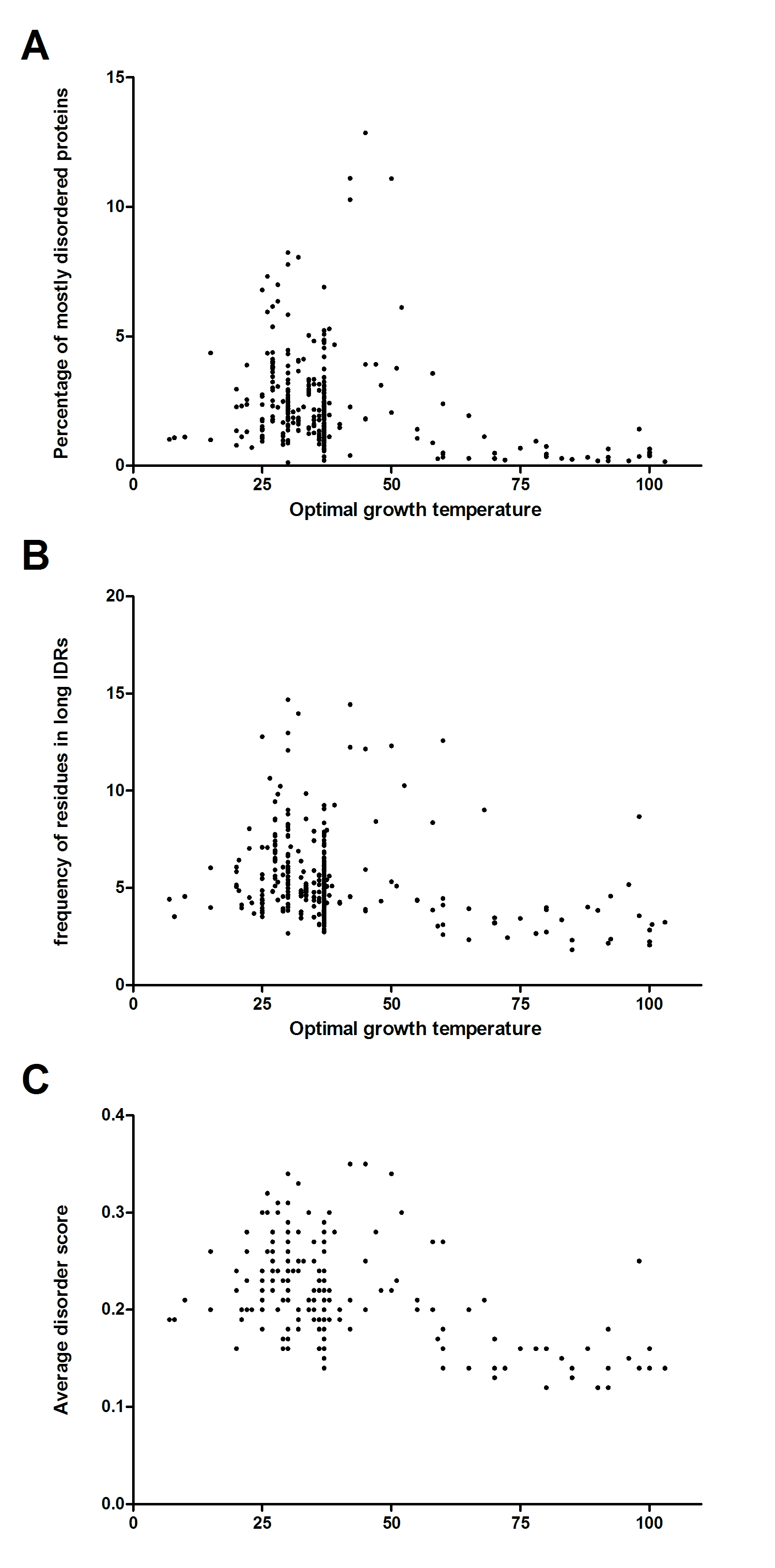

Supplement: Figure S2 — Distribution of various measures of structural disorder as a function of OGT of prokaryotes. (A) Percentage of mostly disordered proteins (more than 50 percent of residues in a protein are disordered), (B) frequency of residues in long IDRs (at least 30 consecutive residues predicted as disordered), (C) total average of disorder scores in whole proteome, in the function of OGT. (0.83 MB TIF) [file pone.0012069.s002.tif]
