# Supplementary material for: Reduction in Structural Disorder and Functional Complexity in the Thermal Adaptation of Prokaryotes
Source: PLoS One. 2010 Aug 11;5(8):e12069. doi: 10.1371/journal.pone.0012069 (PMC2920320; doi:10.1371/journal.pone.0012069)
Supplement: Table S1 — Prokaryote species included in the analysis. (0.10 MB PDF) [file pone.0012069.s003.pdf]

Species	temp range	Opt temp	Average Genome disorder	on Average, the number of residues with disorder >0.5 in genome	percent of proteins with average disorder >0.5	Number of proteins with 50% residues having disorder >0.5	Number of proteins in the genome	number of residues >0.5 in all proteins	total number of residues in the whole proteome of the genome	ratio of 100*column I/column J	FoldIndex
Acidiphilium cryptum JF-5	Mesophilic	35C	0,25	9,3	3,74	119	3559	90364	1180790	7,65284	0,2058
Acidothermus cellulolyticus 11B	Thermophilic	58C	0,27	12,16	4,68	77	2157	79479	729823	10,8902	0,2080
Acinetobacter baumannii	Mesophilic	37C	0,2	5,1	1,37	52	3712	58270	1162802	5,01117	0,1897
Acinetobacter sp. ADP1	Mesophilic	37C	0,19	4,89	1,35	45	3325	48254	1051233	4,59023	0,2027
Actinobacillus succinogenes 130Z	Mesophilic	37C	0,2	4,47	0,82	20	2079	29032	677681	4,28402	0,1593
Aeromonas hydrophila subsp. hydrophila ATCC 7	Mesophilic	22-28C	0,21	5,8	1,5	58	4122	75196	1380361	5,44756	0,1280
Aeropyrum pernix K1	Hyperthermophilic	90-95C	0,18	3,73	0,88	11	1700	15364	492079	3,12226	0,1569
Anaeromyxobacter dehalogenans 2CP-C	Mesophilic	30C	0,28	12,73	5,04	194	4346	178555	1516999	11,7703	0,1901
Anaplasma phagocytophilum HZ	Mesophilic	37C	0,16	4,59	2,29	27	1264	19519	333499	5,85279	0,2024
Aquifex aeolicus VF5	Hyperthermophilic	96C	0,15	2,02	0,38	3	1560	7686	490148	1,5681	0,1966
Archaeoglobus fulgidus DSM 4304	Hyperthermophilic	83C	0,15	1,71	0,21	7	2420	9514	669587	1,42088	0,1413
Arthrobacter aurescens TC1	Mesophilic	30C	0,28	13,03	5,25	198	4587	181054	1520762	11,9055	0,1819
Azoarcus sp. EbN1	Mesophilic	26C	0,26	11,71	6,54	273	4599	127476	1428035	8,92667	0,1850
Bacillus cereus ATCC 10987	Mesophilic	25-35C	0,18	4,73	2,09	118	5844	71583	1533198	4,66887	0,1732
Bacillus subtilis subsp. subtilis str. 168	Mesophilic	25-35C	0,21	6,36	2,41	90	4105	68388	1221709	5,59773	0,2036
Bacteroides fragilis YCH46	Mesophilic	37C	0,18	3,78	0,8	36	4625	52766	1591158	3,3162	0,1998
Bartonella bacilliformis KC583	Mesophilic	28C	0,2	6,24	2,26	29	1283	20227	387541	5,21932	0,2020
Bartonella henselae str. Houston-1	Mesophilic	37C	0,2	6,4	2,49	36	1488	29133	467410	6,23286	0,1517
Bartonella quintana str. Toulouse	Mesophilic	37C	0,2	5,72	1,84	21	1142	19701	379825	5,18686	0,1617
Bdellovibrio bacteriovorus HD100	Mesophilic	28-30C	0,23	7,15	2,56	89	3587	73045	1169120	6,24786	0,2177
Bifidobacterium adolescentis ATCC 15703	Mesophilic	37C	0,28	13,52	6,07	79	1631	76563	601390	12,731	0,1745
Bifidobacterium longum NCC2705	Mesophilic	37-41C	0,28	13,45	5,73	81	1729	85181	641400	13,2805	0,2030
Bordetella bronchiseptica RB50	Mesophilic	35-37C	0,24	7,62	2,36	104	4994	115545	1629896	7,0891	0,1922
Bordetella parapertussis 12822	Mesophilic	35-37C	0,24	7,4	2,27	78	4185	94818	1372931	6,90625	0,1868
Bordetella pertussis Tohama I	Mesophilic	35-37C	0,24	7,28	2,04	57	3436	76329	1122089	6,8024	0,1959
Bradyrhizobium japonicum USDA 110	Mesophilic	25-30C	0,25	10,05	4,08	301	8317	231485	2633756	8,78916	0,1723
Brucella abortus biovar 1 str. 9-941	Mesophilic	37C	0,24	8,25	3,01	91	3085	64620	888817	7,27034	0,1462
Brucella melitensis 16M	Mesophilic	37C	0,24	7,9	2,47	64	3198	67212	949492	7,07873	0,1397
Brucella ovis ATCC 25840	Mesophilic	37C	0,24	7,97	2,66	72	2890	62810	861814	7,28812	0,1565
Brucella suis 1330	Mesophilic	37C	0,23	8,18	2,9	92	3271	66114	930165	7,10777	0,1504
Brucella suis ATCC 23445	Mesophilic	37C	0,23	8,27	3,02	87	3241	67429	923864	7,29859	0,1940
Burkholderia multivorans ATCC 17616	Mesophilic	37C	0,24	8,97	3,32	194	6259	162338	2025361	8,01526	0,1613
Burkholderia thailandensis E264	Mesophilic	25-42C	0,25	10,73	4,47	232	5634	183171	1942079	9,4317	0,1784
Burkholderia xenovorans LB400	Mesophilic	30C	0,24	9,03	3,38	278	8702	218860	2785166	7,85806	0,2049
Caldicellulosiruptor saccharolyticus DSM 8903	Thermophilic	ND	0,14	1,81	0,37	8	2679	13947	852447	1,63611	0,1780
Caldivirga maquilingensis IC-167	Hyperthermophilic	85C	0,14	1,83	0,36	5	1963	8248	601176	1,37198	0,1950
Campylobacter hominis ATCC BAA-381	Mesophilic	37C	0,14	2,78	0,83	17	1687	12354	481482	2,56583	0,1815
Candidatus Methanoregula boonei 6A8	Mesophilic	37C	0,22	6,28	2,04	45	2450	39977	727444	5,49554	0,2005
Carboxydothermus hydrogenoformans Z-2901	Hyperthermophilic	78C	0,16	2,75	0,95	25	2620	14106	721616	1,95478	0,1861
Caulobacter crescentus CB15	Mesophilic	35C	0,27	11,16	5,43	180	3737	112698	1209209	9,31998	0,1742
Chlamydia muridarum Nigg	Mesophilic	37C	0,18	5,37	2,2	18	911	14531	323040	4,4982	0,1625
Chlamydia trachomatis D/UW-3/CX	Mesophilic	37C	0,18	5,82	2,68	22	895	16037	312363	5,13409	0,2030
Chlamydophila caviae GPIC	Mesophilic	37C	0,19	5,87	2,09	22	1005	18694	350813	5,32876	0,1945

Chlamydophila pneumoniae AR39	Mesophilic	37C	0,18	5,83	2,34	24	1112	17978	364115	4,93745	0,1775
Chlamydophila pneumoniae CWL029	Mesophilic	37C	0,18	5,81	2,38	21	1052	17839	362041	4,92734	0,1777
Chlamydophila pneumoniae J138	Mesophilic	37C	0,19	5,82	2,43	22	1069	18008	366322	4,91589	0,1666
Chlorobium tepidum TLS	Thermophilic	48C	0,22	7,37	3,24	70	2252	32527	630738	5,15697	0,1569
Chromobacterium violaceum ATCC 12472	Mesophilic	25C	0,23	7,79	2,88	121	4407	102299	1398086	7,31707	0,1764
Chromohalobacter salexigens DSM 3043	Mesophilic	37C	0,26	10,22	3,73	107	3298	103630	1099708	9,42341	0,1729
Clavibacter michiganensis subsp. michiganensis I	Mesophilic	25-28C	0,3	14,81	5,36	134	3079	140121	1007926	13,9019	0,1880
Clostridium acetobutylicum ATCC 824	Mesophilic	10-65C	0,17	2,83	0,75	24	3848	31720	1177004	2,69498	0,1897
Clostridium botulinum A str. ATCC 19397	Mesophilic	37C	0,16	3,11	1,04	34	3590	26378	1052834	2,50543	0,1798
Clostridium botulinum A str. ATCC 3502	Mesophilic	37C	0,16	3,08	1	35	3552	26620	1042007	2,55469	0,1915
Clostridium botulinum A str. Hall	Mesophilic	37C	0,16	3,03	1,03	32	3404	24669	1007070	2,44958	0,1523
Clostridium botulinum A3 str. Loch Maree	Mesophilic	37C	0,16	3,04	0,9	37	3984	29680	1148930	2,58327	0,1534
Clostridium botulinum B1 str. Okra	Mesophilic	37C	0,16	3,1	1,01	36	3852	28245	1105625	2,55466	0,1618
Clostridium botulinum F str. Langeland	Mesophilic	37C	0,17	3,13	0,98	33	3659	27923	1075716	2,59576	0,2119
Clostridium difficile 630	Mesophilic	37C	0,17	2,82	0,8	25	3753	32879	1173228	2,80244	0,1098
Clostridium perfringens ATCC 13124	Mesophilic	37C	0,17	3,39	1,15	25	2723	25997	856066	3,0368	0,1465
Clostridium perfringens SM101	Mesophilic	37C	0,17	3,2	1,06	31	2876	28989	899399	3,22315	0,1609
Clostridium perfringens str. 13	Mesophilic	37C	0,17	3,21	1,03	27	2631	22509	797744	2,82158	0,1705
Clostridium phytofermentans ISDg	Mesophilic	37C	0,17	3,4	1	35	3902	44021	1318750	3,33809	0,1950
Clostridium tetani E88	Mesophilic	37C	0,16	2,29	0,29	5	2432	17845	814431	2,1911	0,1851
Clostridium thermocellum ATCC 27405	Thermophilic	60C	0,18	3,4	0,75	16	3189	37328	1068839	3,49239	0,1920
Colwellia psychrerythraea 34H	Psychrophilic	8C	0,19	4,43	1,08	53	4910	65172	1512173	4,30982	0,1841
Corynebacterium diphtheriae NCTC 13129	Mesophilic	37C	0,27	11,1	4,05	85	2272	75389	727426	10,3638	0,1801
Corynebacterium efficiens YS-314	Mesophilic	30-45C	0,3	14,97	6,2	156	2950	131263	958318	13,6972	0,1503
Corynebacterium glutamicum R	Mesophilic	30-40C	0,27	10,96	3,96	97	3080	98542	962200	10,2413	0,1839
Coxiella burnetii RSA 493	Mesophilic	37C	0,2	6,92	3,65	70	2052	26827	570164	4,70514	0,1491
Cytophaga hutchinsonii ATCC 33406	Mesophilic	30C	0,16	3,13	0,98	37	3785	36596	1327846	2,75604	0,1925
Dehalococcoides ethenogenes 195	Mesophilic	35C	0,19	4,81	1,46	20	1580	18292	438843	4,16823	0,1959
Deinococcus geothermalis DSM 11300	Mesophilic	47C	0,28	12,11	4,74	120	3062	107725	970757	11,097	0,1771
Deinococcus radiodurans R1	Mesophilic	30-37C	0,3	13,86	6,13	160	3181	130115	985225	13,2066	0,2109
Delftia acidovorans SPH-1	Mesophilic	30C	0,26	10,22	3,08	154	6040	194023	2020772	9,60143	0,1569
Desulfotalea psychrophila LSv54	Psychrophilic	7C	0,19	4,25	0,93	33	3234	41934	1036990	4,04382	0,1760
Desulfotomaculum reducens MI-1	Mesophilic	37C	0,2	4,5	1,19	33	3276	40190	999111	4,02258	0,2097
Desulfovibrio desulfuricans G20	Mesophilic	25-40C	0,24	8,99	3,84	138	3775	82351	1121515	7,34284	0,1943
Desulfovibrio vulgaris subsp. vulgaris str. Hildenb	Mesophilic	25-40C	0,25	10,43	4,39	144	3531	99335	1078093	9,21395	0,2034
Dichelobacter nodosus VCS1703A	Mesophilic	37C	0,19	5	1,48	16	1280	21856	417601	5,2337	0,2156
Dinoroseobacter shibae DFL 12	Mesophilic	33C	0,25	8,8	2,87	95	4187	110459	1317230	8,3857	0,1165
Enterobacter sakazakii ATCC BAA-894	Mesophilic	37C	0,22	6,77	2,35	104	4434	82035	1342332	6,11138	0,1638
Enterococcus faecalis V583	Mesophilic	37C	0,21	6,33	2,85	74	3265	62870	960383	6,54635	0,1929
Escherichia coli CFT073	Mesophilic	37C	0,21	6,1	2,06	105	5379	84947	1569865	5,4111	0,2019
Escherichia coli DH10B	Mesophilic	37C	0,21	5,68	1,62	64	4126	65495	1297117	5,04927	0,1903
Escherichia coli E24377A	Mesophilic	37C	0,21	6,25	2,04	95	4997	82398	1487042	5,54107	0,1783
Escherichia coli HS	Mesophilic	37C	0,21	5,79	1,76	72	4384	69230	1335595	5,18346	0,1855
Escherichia coli W3110	Mesophilic	37C	0,21	5,6	1,51	61	4226	68095	1339321	5,08429	0,1604
Fervidobacterium nodosum Rt17-B1	Thermophilic	70C	0,14	1,9	0,29	5	1750	8926	585328	1,52496	0,1720
Flavobacterium johnsoniae UW101	Mesophilic	20-30C	0,18	3,36	1,08	47	5017	54723	1769529	3,09252	0,1548
Fusobacterium nucleatum subsp. nucleatum ATC	Mesophilic	37C	0,15	2,61	1,02	22	2067	15617	643488	2,42693	0,1090
Geobacillus kaustophilus HTA426	Thermophilic	ND	0,21	6,49	2,77	95	3540	48731	1014285	4,80447	0,2025

Geobacillus thermodenitrificans NG80-2	Thermophilic	65C	0,2	5,43	2,15	67	3445	44507	1003478	4,43527	0,2220
Geobacter metallireducens GS-15	Mesophilic	30C	0,22	6,06	1,81	54	3532	66541	1211145	5,49406	0,1559
Geobacter sulfurreducens PCA	Mesophilic	30C	0,22	6,87	2,41	76	3446	66680	1137980	5,85951	0,1807
Gluconacetobacter diazotrophicus PAl 5	Mesophilic	30C	0,27	12,92	6,28	225	3852	126157	1197244	10,5373	0,2011
Gluconobacter oxydans 621H	Mesophilic	25-30C	0,28	12,25	4,58	100	2664	94256	870283	10,8305	0,1592
Haemophilus ducreyi 35000HP	Mesophilic	35-37C	0,18	4,49	1,16	23	1717	22042	480322	4,589	0,1550
Haemophilus influenzae Rd KW20	Mesophilic	35-37C	0,19	4,32	1,09	14	1657	21013	516236	4,07043	0,1577
Haemophilus somnus 129PT	Mesophilic	35-37C	0,18	4,07	1,17	20	1798	33455	589704	5,67319	0,1556
Haemophilus somnus 2336	Mesophilic	35-37C	0,19	4,46	1,16	24	1980	38208	657271	5,81313	0,1427
Haloarcula marismortui ATCC 43049	Mesophilic	40-50C	0,35	24,04	14,25	545	4240	284145	1203534	23,6092	0,2264
Halobacterium salinarum R1	Thermophilic	50C	0,34	21,88	11,86	305	2749	160850	774914	20,7571	0,1740
Halobacterium sp. NRC-1	Mesophilic	42C	0,35	22,15	12,2	291	2622	156490	737458	21,2202	0,1923
Helicobacter acinonychis str. Sheeba	Mesophilic	37C	0,17	4,72	1,73	29	1618	18086	464163	3,89648	0,1526
Helicobacter hepaticus ATCC 51449	Mesophilic	37C	0,16	3,51	1,12	18	1875	17008	558337	3,04619	0,1832
Helicobacter pylori 26695	Mesophilic	37C	0,17	5,42	2,41	36	1576	22855	501419	4,55806	0,1340
Helicobacter pylori HPAG1	Mesophilic	37C	0,17	5,13	2,4	38	1544	22042	489511	4,50286	0,1574
Helicobacter pylori J99	Mesophilic	37C	0,18	5,32	2,42	37	1489	23208	494009	4,69789	0,1593
Heliobacterium modesticaldum Ice1	Thermophilic	50-52C	0,23	8,64	4	113	3000	59486	880842	6,75331	0,1553
Hyperthermus butylicus DSM 5456	Hyperthermophilic	95-106C	0,16	3,02	0,5	7	1602	10299	449798	2,28969	0,1465
Hyphomonas neptunium ATCC 15444	Mesophilic	37C	0,26	9,85	3,57	99	3505	95495	1114601	8,56764	0,1711
Idiomarina loihiensis L2TR	Mesophilic	4-46C	0,24	8,02	2,51	62	2628	64357	869940	7,39787	0,2011
Jannaschia sp. CCS1	Mesophilic	30C	0,25	8,6	2,64	100	4283	111167	1327554	8,37382	0,1694
Kineococcus radiotolerans SRS30216	Mesophilic	32C	0,33	18,51	9,74	377	4681	242584	1489738	16,2837	0,1802
Klebsiella pneumoniae subsp. pneumoniae MGH	Mesophilic	37C	0,21	5,71	1,7	83	5187	85047	1619320	5,25202	0,1892
Lactobacillus acidophilus NCFM	Mesophilic	25-35C	0,22	6,53	2,69	39	1862	43542	584499	7,44946	0,1914
Lactobacillus brevis ATCC 367	Mesophilic	25-35C	0,22	7,21	2,57	47	2218	45008	650815	6,91564	0,1882
Lactobacillus casei ATCC 334	Mesophilic	30-40C	0,22	7,12	2,35	60	2771	54370	798238	6,81125	0,1778
Lactobacillus delbrueckii subsp. bulgaricus ATCC	Mesophilic	42C	0,21	6,03	2,32	39	1721	24079	475905	5,05962	0,1889
Lactobacillus gasseri ATCC 33323	Mesophilic	25-35C	0,22	6,37	2,22	33	1755	39654	556923	7,12019	0,1542
Lactobacillus johnsonii NCC 533	Mesophilic	25-35C	0,21	6,24	2,58	42	1821	47289	591376	7,99644	0,2029
Lactobacillus plantarum WCFS1	Mesophilic	25-35C	0,21	6,01	2,19	54	3057	60315	931444	6,47543	0,2095
Lactococcus lactis subsp. cremoris MG1363	Mesophilic	40C	0,2	4,92	1,68	36	2434	33616	692229	4,8562	0,1885
Lactococcus lactis subsp. cremoris SK11	Mesophilic	40C	0,19	4,87	1,68	40	2504	32025	693740	4,61628	0,1499
Leifsonia xyli subsp. xyli str. CTCB07	Mesophilic	20-25C	0,28	12,53	5,07	79	2030	67340	602316	11,1802	0,1508
Leptospira interrogans serovar Copenhageni str.	Mesophilic	28-30C	0,17	3,73	0,93	30	3658	42528	1151209	3,6942	0,1805
Leptospira interrogans serovar Lai str. 56601	Mesophilic	28-30C	0,16	3,59	1,04	46	4727	44878	1221026	3,67543	0,1647
Leuconostoc mesenteroides subsp. mesenteroid	Mesophilic	20-30C	0,21	5,95	1,95	36	2005	34268	606767	5,64764	0,2003
Listeria innocua Clip11262	Mesophilic	30-37C	0,21	5,24	1,61	45	3043	46562	911734	5,10697	0,1926
Listeria monocytogenes EGD-e	Mesophilic	30-37C	0,2	4,89	1,51	41	2846	42743	870846	4,90822	0,1795
Listeria monocytogenes str. 4b F2365	Mesophilic	30-37C	0,2	4,81	1,45	35	2821	41317	853671	4,83992	0,1764
Mannheimia succiniciproducens MBEL55E	Mesophilic	37C	0,18	4,32	1,22	33	2380	27721	689990	4,01759	0,1717
Maricaulis maris MCS10	Mesophilic	20-25C	0,26	9,82	3,04	78	3063	93313	1006730	9,26892	0,1702
Marinobacter aquaeolei VT8	Mesophilic	30C	0,26	9,8	3,18	116	4272	130268	1423715	9,14986	0,1543
Mesoplasma florum L1	Mesophilic	20-40C	0,17	3,28	0,88	6	682	7051	244942	2,87864	0,1673
Metallosphaera sedula DSM 5348	Thermophilic	70C	0,17	2,58	0,4	11	2256	13535	640287	2,1139	0,2172
Methanobrevibacter smithii ATCC 35061	Mesophilic	37-40C	0,19	4,01	1,06	20	1793	19020	555768	3,42229	0,1753
Methanococcoides burtonii DSM 6242	Mesophilic	23.4C	0,2	4,21	0,88	16	2273	27034	687337	3,93315	0,1944
Methanococcus aeolicus Nankai-3	Mesophilic	42C	0,18	2,88	0,47	6	1490	10911	439206	2,48426	0,1712

Methanococcus maripaludis S2	Mesophilic	35-40C	0,17	2,5	0,41	6	1722	10639	490051	2,171	0,1844
Methanococcus vannielii SB	Mesophilic	30C	0,16	1,88	0,12	2	1678	8033	489682	1,64045	0,1484
Methanocorpusculum labreanum Z	Mesophilic	37C	0,19	3,57	0,75	10	1739	17193	522502	3,29051	0,1587
Methanoculleus marisnigri JR1	Mesophilic	21-25C	0,23	7,06	2,73	59	2489	42941	719678	5,9667	0,1421
Methanopyrus kandleri AV19	Hyperthermophilic	98C	0,25	7,51	1,9	24	1687	35652	501100	7,11475	0,2406
Methanosaeta thermophila PT	Thermophilic	ND	0,2	3,72	0,47	7	1696	18837	513525	3,66818	0,1950
Methanosarcina acetivorans C2A	Mesophilic	35-40C	0,2	5,27	1,43	58	4540	76140	1415838	5,37773	0,2115
Methanosarcina mazei Go1	Mesophilic	30-40C	0,2	5,39	1,81	52	3370	53447	1021269	5,23339	0,1390
Methanosphaera stadtmanae DSM 3091	Mesophilic	36-40C	0,21	5,21	2,15	30	1534	23510	493971	4,75939	0,2109
Methanospirillum hungatei JF-1	Mesophilic	37C	0,2	4,69	1,02	29	3139	48720	1014087	4,80432	0,1570
Methylibium petroleiphilum PM1	Mesophilic	30C	0,26	9,81	3,33	131	4449	119374	1412766	8,44967	0,2018
Methylobacillus flagellatus KT	Mesophilic	30-42C	0,22	6,63	2,07	53	2753	53764	890782	6,0356	0,1983
Methylobacterium extorquens PA1	Mesophilic	25-30C	0,28	13,49	7,04	297	4829	170083	1527522	11,1346	0,1565
Methylococcus capsulatus str. Bath	Thermophilic	45C	0,25	9,51	4,47	116	2956	74550	982447	7,5882	0,1912
Moorella thermoacetica ATCC 39073	Thermophilic	58C	0,2	4,84	1,38	22	2465	33641	755527	4,45265	0,1847
Mycobacterium avium 104	Mesophilic	37C	0,29	13,27	6,33	260	5120	180531	1612773	11,1938	0,1968
Mycobacterium avium subsp. paratuberculosis K	Mesophilic	37C	0,29	13,12	5,47	198	4350	178532	1469173	12,1519	0,1949
Mycobacterium bovis AF2122/97	Mesophilic	37C	0,28	12,37	5,46	189	3920	151453	1307802	11,5807	0,2140
Mycobacterium leprae TN	Mesophilic	37C	0,26	9,92	3,36	45	1605	46953	538437	8,72024	0,1592
Mycobacterium smegmatis str. MC2 155	Mesophilic	37C	0,29	12,95	6,03	351	6716	232966	2118292	10,9978	0,1670
Mycobacterium tuberculosis CDC1551	Mesophilic	37C	0,29	14,04	7,28	289	4189	160925	1326524	12,1313	0,2006
Mycobacterium tuberculosis F11	Mesophilic	37C	0,28	12,38	5,33	189	3941	156931	1339537	11,7153	0,1940
Mycobacterium tuberculosis H37Rv	Mesophilic	37C	0,28	12,4	5,39	194	3989	155377	1336162	11,6286	0,1784
Mycobacterium ulcerans Agy99	Mesophilic	32C	0,28	11,88	4,64	168	4160	149512	1353971	11,0425	0,1785
Mycobacterium vanbaalenii PYR-1	Mesophilic	24-37C	0,28	12,19	4,83	231	5979	227615	1974441	11,5281	0,1498
Mycoplasma agalactiae PG2	Psychrophilic	ND	0,18	4,95	2,56	17	742	10880	255606	4,25655	0,1706
Mycoplasma capricolum subsp. capricolum ATCC	Mesophilic	37C	0,17	4,84	1,72	12	812	12619	296533	4,25551	0,1601
Mycoplasma gallisepticum R	Mesophilic	37C	0,19	6,92	2,48	12	726	24048	291039	8,26281	0,1671
Mycoplasma genitalium G37	Mesophilic	37C	0,16	4,86	2,1	11	477	8436	175727	4,80063	0,1571
Mycoplasma hyopneumoniae 232	Mesophilic	37C	0,14	4	1,16	7	691	12153	268125	4,53259	0,1621
Mycoplasma hyopneumoniae 7448	Mesophilic	37C	0,15	4,05	1,06	7	663	11637	263225	4,42093	0,1607
Mycoplasma hyopneumoniae J	Mesophilic	37C	0,15	4,39	1,5	10	665	11768	261815	4,49478	0,1573
Mycoplasma mobile 163K	Mesophilic	20C	0,16	3,51	1,58	5	633	5574	234509	2,37688	0,2101
Mycoplasma mycoides subsp. mycoides SC str. P	Mesophilic	37C	0,16	4,37	1,87	12	1016	11621	329458	3,52731	0,2017
Mycoplasma penetrans HF-2	Mesophilic	37C	0,18	5,76	1,35	15	1037	21341	400551	5,32791	0,1235
Mycoplasma pneumoniae M129	Mesophilic	37C	0,21	8,58	3,63	29	689	21393	239716	8,92431	0,2038
Mycoplasma pulmonis UAB CTIP	Mesophilic	37C	0,17	5,38	2,69	19	782	15924	290153	5,48814	0,1908
Mycoplasma synoviae 53	Mesophilic	37C	0,18	5,36	1,04	6	672	11215	237323	4,72563	0,1685
Myxococcus xanthus DK 1622	Mesophilic	20-30C	0,3	15,53	7,97	498	7331	393687	2780010	14,1614	0,1635
Nanoarchaeum equitans Kin4-M	Hyperthermophilic	90C	0,12	1,75	0,19	1	536	1820	151570	1,20077	0,1921
Neisseria gonorrhoeae FA 1090	Mesophilic	35-37C	0,24	8,51	3,65	63	2002	42997	562017	7,65048	0,1470
Neisseria meningitidis FAM18	Mesophilic	35-37C	0,22	6,91	2,03	34	1917	41384	585009	7,07408	0,1354
Neisseria meningitidis MC58	Mesophilic	35-37C	0,23	7,79	3,3	60	2063	45894	597438	7,6818	0,2086
Neisseria meningitidis Z2491	Mesophilic	35-37C	0,23	7,29	2,54	44	2049	40370	583381	6,92001	0,1819
Nitratiruptor sp. SB155-2	Thermophilic	ND	0,16	2,48	0,54	8	1843	12403	587077	2,11267	0,1868
Nocardioides sp. JS614	Mesophilic	30C	0,29	13,07	4,28	176	4909	195585	1599588	12,2272	0,1895
Oceanobacillus iheyensis HTE831	Mesophilic	30C	0,21	6,91	2,77	88	3500	62369	1018006	6,12658	0,2099
Oenococcus oeni PSU-1	Mesophilic	17-25C	0,19	5,37	2,66	39	1691	22497	487917	4,61083	0,1915

Paracoccus denitrificans PD1222	Mesophilic	25-30C	0,26	9,86	3,47	164	5077	139079	1579571	8,80486	0,1197
Pasteurella multocida subsp. multocida str. Pm7(	Mesophilic	37C	0,18	3,78	0,74	16	2015	26681	668007	3,99412	0,1381
Pediococcus pentosaceus ATCC 25745	Mesophilic	30C	0,22	6,63	2,56	40	1755	34890	535200	6,51906	0,1832
Pelobacter propionicus DSM 2379	Mesophilic	30C	0,22	6,94	1,97	60	3804	77547	1240249	6,25253	0,1529
Pelodictyon luteolum DSM 273	Mesophilic	25C	0,22	6,62	1,44	24	2083	40106	700673	5,72393	0,1744
Pelotomaculum thermopropionicum SI	Thermophilic	55C	0,2	4,83	1,34	31	2920	35374	868388	4,07352	0,1625
Petrotoga mobilis SJ95	Thermophilic	ND	0,16	2,36	0,42	6	1898	13358	642353	2,07954	0,1804
Photobacterium profundum SS9	Psychrophilic	15C	0,2	4,72	1,11	55	5489	85252	1733919	4,91672	0,1937
Picrophilus torridus DSM 9790	Thermophilic	60C	0,14	1,94	0,59	7	1535	6056	471359	1,2848	0,1077
Polaromonas naphthalenivorans CJ2	Mesophilic	20C	0,24	8,45	2,86	124	5453	118074	1714773	6,88569	0,1952
Polaromonas sp. JS666	Mesophilic	20C	0,24	7,91	2,44	146	4929	115482	1575666	7,32909	0,1736
Porphyromonas gingivalis W83	Mesophilic	37C	0,2	5,55	1,83	31	1909	27836	643202	4,32772	0,1548
Propionibacterium acnes KPA171202	Mesophilic	37C	0,29	13,62	5,53	109	2297	94292	761895	12,376	0,1907
Pseudoalteromonas haloplanktis TAC125	Psychrophilic	ND	0,2	4,67	1,41	40	3486	48053	1116827	4,30264	0,1790
Pseudomonas aeruginosa PA7	Mesophilic	25-30C	0,24	9,2	4,22	96	5568	119146	1861588	6,40023	0,1522
Pseudomonas aeruginosa PAO1	Mesophilic	25-30C	0,23	6,9	1,89	259	6286	138811	1961256	7,07766	0,1560
Pseudomonas aeruginosa UCBPP-PA14	Mesophilic	25-30C	0,23	7,19	2,1	112	5892	128097	1942833	6,59331	0,1547
Pseudomonas fluorescens Pf-5	Mesophilic	25-30C	0,22	6,83	2,02	109	6138	129459	2087344	6,20209	0,1603
Psychrobacter cryohalolentis K5	Psychrophilic	ND	0,23	7,98	2,39	54	2511	63463	855759	7,41599	0,1242
Psychrobacter sp. PRwf-1	Mesophilic	4-37C	0,24	9,21	2,94	64	2385	81068	826440	9,8093	0,1781
Psychromonas ingrahamii 37	Psychrophilic	ND	0,18	3,69	0,85	29	3545	40918	1176783	3,47711	0,1904
Pyrobaculum aerophilum str. IM2	Hyperthermophilic	100C	0,14	2,36	0,65	17	2605	11295	655910	1,72204	0,2132
Pyrobaculum arsenaticum DSM 13514	Hyperthermophilic	ND	0,14	2,22	0,48	8	2299	10654	611416	1,74251	0,1874
Pyrobaculum calidifontis JCM 11548	Hyperthermophilic	90-95C	0,14	2,13	0,47	7	2149	10486	605656	1,73135	0,2182
Pyrobaculum islandicum DSM 4184	Thermophilic	100C	0,14	2,42	0,71	10	1978	9231	528999	1,74499	0,2135
Pyrococcus abyssi GE5	Hyperthermophilic	103C	0,14	1,65	0,26	3	1898	7629	549764	1,38769	0,1926
Pyrococcus furiosus DSM 3638	Hyperthermophilic	100C	0,14	1,86	0,47	8	2125	8182	586906	1,39409	0,2319
Pyrococcus horikoshii OT3	Hyperthermophilic	98C	0,14	1,88	0,46	7	1955	7857	548155	1,43335	0,2147
Ralstonia eutropha H16	Mesophilic	30C	0,25	8,64	2,85	163	6626	165647	2161404	7,66386	0,1485
Ralstonia eutropha JMP134	Mesophilic	30C	0,25	8,84	3,09	185	6446	167235	2113107	7,91418	0,2130
Ralstonia metallidurans CH34	Mesophilic	30C	0,25	9,26	2,99	154	6319	169351	2038372	8,30815	0,1288
Renibacterium salmoninarum ATCC 33209	Mesophilic	15C	0,26	10,48	5,08	153	3507	89701	946584	9,47629	0,2053
Rhizobium leguminosarum bv. viciae 3841	Mesophilic	25-30C	0,24	7,91	2,94	180	7143	152541	2231743	6,83506	0,1880
Rhodobacter sphaeroides ATCC 17025	Mesophilic	25-35C	0,26	10,41	4,2	155	4333	121863	1334338	9,13284	0,1960
Rhodobacter sphaeroides ATCC 17029	Mesophilic	25-35C	0,25	9,43	3,61	137	4132	112619	1320298	8,52982	0,1445
Rhodococcus sp. RHA1	Mesophilic	30C	0,31	16,08	8,58	711	9145	382763	2889415	13,2471	0,1434
Rhodoferax ferrireducens T118	Mesophilic	25C	0,22	6,42	1,72	67	4418	83366	1478398	5,63894	0,1468
Rhodopseudomonas palustris BisA53	Mesophilic	25-30C	0,25	10,06	4,14	186	4878	135209	1570051	8,61176	0,2219
Rhodopseudomonas palustris BisB18	Mesophilic	25-30C	0,25	9,99	4,46	196	4886	129802	1567480	8,28094	0,1724
Rhodopseudomonas palustris BisB5	Mesophilic	25-30C	0,25	10,12	4,53	174	4397	125150	1412295	8,86146	0,1857
Rhodopseudomonas palustris CGA009	Mesophilic	25-35C	0,25	9,64	3,98	166	4820	133055	1582923	8,40565	0,1288
Rhodopseudomonas palustris HaA2	Mesophilic	25-30C	0,25	9,69	4,06	180	4683	129691	1546520	8,38599	0,2198
Rhodospirillum rubrum ATCC 11170	Mesophilic	25-30C	0,25	9,28	3,54	112	3841	106006	1293864	8,19298	0,1793
Roseiflexus castenholzii DSM 13941	Thermophilic	50C	0,22	7,19	2,24	89	4330	111819	1564659	7,14654	0,1527
Roseiflexus sp. RS-1	Thermophilic	ND	0,22	7,55	2,15	84	4517	125178	1641453	7,62605	0,1800
Rubrobacter xylanophilus DSM 9941	Thermophilic	60C	0,27	10,59	2,93	75	3140	95454	985393	9,6869	0,1804
Saccharophagus degradans 2-40	Mesophilic	4-37C	0,22	6,25	1,52	54	4008	96793	1457661	6,6403	0,2261
Saccharopolyspora erythraea NRRL 2338	Mesophilic	25-32 (28)C	0,31	16,35	8,36	503	7197	356241	2317888	15,3692	0,1870

Salinibacter ruber DSM 13855	Mesophilic	37-47C	0,35	22,62	12,14	291	2833	223011	1011954	22,0377	0,1363
Salinispora tropica CNB-440	Mesophilic	28C	0,3	15,53	7,45	288	4536	220640	1520499	14,511	0,1810
Salmonella enterica subsp. enterica serovar Chol	Mesophilic	37C	0,21	6,15	2,19	96	4648	77380	1375501	5,62559	0,2042
Salmonella enterica subsp. enterica serovar Para	Mesophilic	37C	0,2	6,5	2,87	146	5601	79869	1446025	5,52335	0,1656
Salmonella typhimurium LT2	Mesophilic	37C	0,21	5,92	1,81	75	4527	78600	1419886	5,53566	0,1524
Shewanella amazonensis SB2B	Mesophilic	37C	0,21	5,41	1,21	40	3645	67014	1261259	5,31326	0,1369
Shewanella denitrificans OS217	Mesophilic	20-25C	0,2	4,94	1,36	49	3754	62371	1276492	4,88613	0,1791
Shewanella frigidimarina NCIMB 400	Mesophilic	20-22C	0,2	4,76	1,14	45	4029	62003	1355375	4,5746	0,1530
Shewanella sediminis HAW-EB3	Psychrophilic	10C	0,21	5,22	1,22	50	4497	78935	1524193	5,17881	0,1835
Shewanella woodyi ATCC 51908	Mesophilic	25C	0,2	4,75	1,09	52	4880	89996	1679397	5,35883	0,1513
Shigella boydii Sb227	Mesophilic	37C	0,22	6,54	1,87	70	4285	67447	1240038	5,43911	0,1866
Shigella dysenteriae Sd197	Mesophilic	37C	0,22	5,4	1,46	59	4506	60832	1168017	5,20814	0,1783
Shigella flexneri 2a str. 2457T	Mesophilic	37C	0,22	6,06	1,62	55	4445	69514	1282744	5,41916	0,1555
Shigella flexneri 2a str. 301	Mesophilic	37C	0,22	6,22	1,69	58	4068	62442	1183933	5,27412	0,1759
Shigella flexneri 5 str. 8401	Mesophilic	37C	0,22	6,07	1,43	53	4116	65005	1228721	5,29046	0,1868
Shigella sonnei Ss046	Mesophilic	37C	0,22	5,85	1,65	69	4475	72360	1348708	5,36513	0,1601
Sinorhizobium medicae WSM419	Mesophilic	28C	0,24	8,36	3,43	190	6213	135931	1938198	7,01327	0,2014
Sinorhizobium meliloti 1021	Mesophilic	25-30C	0,24	8,44	3,29	187	6205	135449	1917146	7,06514	0,1696
Solibacter usitatus Ellin6076	Mesophilic	30C	0,25	8,32	2,26	143	7826	230342	2997215	7,6852	0,1624
Staphylococcus aureus subsp. aureus COL	Mesophilic	30-37C	0,21	6,49	3,63	81	2618	58593	788421	7,43169	0,1939
Staphylococcus aureus subsp. aureus JH1	Mesophilic	30-37C	0,21	6,71	3,53	86	2780	53713	766424	7,00826	0,1419
Staphylococcus aureus subsp. aureus JH9	Mesophilic	30-37C	0,21	6,78	3,56	85	2726	63788	772955	8,25249	0,1456
Staphylococcus aureus subsp. aureus MRSA252	Mesophilic	30-37C	0,21	6,3	3,24	73	2656	66397	816155	8,13534	0,1429
Staphylococcus aureus subsp. aureus MSSA476	Mesophilic	30-37C	0,2	6,42	3,46	76	2598	66179	813148	8,13862	0,2090
Staphylococcus aureus subsp. aureus Mu3	Mesophilic	3-37C	0,21	6,49	3,41	78	2698	64400	802271	8,02721	0,1657
Staphylococcus aureus subsp. aureus Mu50	Mesophilic	30-37C	0,21	6,52	3,44	79	2731	64440	808163	7,97364	0,1614
Staphylococcus aureus subsp. aureus MW2	Mesophilic	30-37C	0,21	6,45	3,5	78	2632	63417	784009	8,08881	0,1917
Staphylococcus aureus subsp. aureus N315	Mesophilic	30-37C	0,21	6,33	3,32	74	2619	62195	787210	7,90069	0,1724
Staphylococcus aureus subsp. aureus NCTC 8325	Mesophilic	30-37C	0,2	6,54	3,56	95	2892	63471	797443	7,95931	0,1592
Staphylococcus aureus subsp. aureus USA300	Mesophilic	37C	0,21	6,66	3,53	80	2604	64029	796141	8,04242	0,2086
Staphylococcus aureus subsp. aureus USA300_T(	Mesophilic	37C	0,2	6,22	3,21	78	2683	59196	772140	7,66649	0,1744
Staphylococcus epidermidis ATCC 12228	Mesophilic	30-37C	0,21	7,31	3,66	83	2485	60223	711855	8,46001	0,1862
Staphylococcus epidermidis RP62A	Mesophilic	30-37C	0,21	6,76	3,13	70	2526	61280	725515	8,44641	0,2212
Staphylococcus haemolyticus JCSC1435	Mesophilic	30-37C	0,21	6,9	3,33	88	2676	63668	767364	8,29698	0,1736
Staphylothermus marinus F1	Hyperthermophilic	92C	0,12	1,74	0,32	3	1570	5660	454821	1,24445	0,1902
Streptococcus agalactiae 2603V/R	Mesophilic	37C	0,19	4,65	1,51	33	2124	29121	621081	4,68876	0,1731
Streptococcus agalactiae A909	Mesophilic	37C	0,19	4,64	1,5	31	1996	29041	609422	4,76534	0,1958
Streptococcus agalactiae NEM316	Mesophilic	37C	0,19	5,22	2,05	41	2094	33859	644518	5,25338	0,1404
Streptococcus gordonii str. Challis substr. CH1	Mesophilic	37C	0,19	5,06	2,1	38	2051	41285	642413	6,42655	0,1931
Streptococcus mutans UA159	Mesophilic	37C	0,18	3,99	1,33	27	1960	23356	579731	4,02877	0,1383
Streptococcus pneumoniae D39	Mesophilic	30-35C	0,19	4,54	1,83	34	1914	27681	568862	4,86603	0,1558
Streptococcus pneumoniae R6	Mesophilic	30-35C	0,19	4,23	1,57	28	2043	28162	588817	4,78281	0,2022
Streptococcus pneumoniae TIGR4	Mesophilic	30-35C	0,18	4,57	2,14	39	2105	27138	599453	4,52713	0,1584
Streptococcus pyogenes M1 GAS	Mesophilic	30-35C	0,2	5,06	2,06	29	1697	25226	515662	4,89196	0,1625
Streptococcus pyogenes MGAS10394	Mesophilic	35C	0,2	5,15	1,86	27	1745	23753	512089	4,63845	0,1557
Streptococcus pyogenes MGAS315	Mesophilic	30-35C	0,2	4,88	1,88	33	1886	27784	550166	5,05011	0,1347
Streptococcus pyogenes MGAS5005	Mesophilic	35C	0,19	4,85	1,98	30	1865	25869	541788	4,77475	0,1165
Streptococcus pyogenes MGAS6180	Mesophilic	35C	0,19	5,19	2,38	33	1865	24833	529358	4,69115	0,1895

Streptococcus pyogenes MGAS8232	Mesophilic	30-35C	0,2	4,96	1,69	41	1894	30159	548400	5,49945	0,1641
Streptococcus pyogenes SSI-1	Mesophilic	30-35C	0,2	4,95	1,93	25	1839	25674	535851	4,79126	0,1935
Streptococcus pyogenes str. Manfredo	Mesophilic	30-35C	0,2	4,95	1,72	30	1861	25638	534381	4,7977	0,2045
Streptococcus suis 05ZYH33	Mesophilic	37C	0,19	4,57	1,83	36	2186	29023	610698	4,75243	0,1440
Streptococcus suis 98HAH33	Mesophilic	37C	0,19	4,51	1,65	34	2185	28566	610686	4,67769	0,1805
Streptococcus thermophilus CNRZ1066	Thermophilic	45C	0,2	4,88	2,14	35	1915	21265	500777	4,2464	0,1685
Streptococcus thermophilus LMD-9	Mesophilic	37C	0,2	5,62	2,56	42	1716	22034	475746	4,63146	0,1552
Streptococcus thermophilus LMG 18311	Thermophilic	45C	0,2	4,94	2,17	34	1889	21912	501240	4,37156	0,1885
Streptomyces avermitilis MA-4680	Mesophilic	25-35 (26)C	0,32	17,91	8,99	561	7676	442540	2614811	16,9244	0,1873
Streptomyces coelicolor A3(2)	Mesophilic	25-35C	0,34	19,45	10,06	671	8154	491025	2661507	18,4491	0,1326
Sulfolobus acidocaldarius DSM 639	Thermophilic	70-75C	0,14	1,74	0,27	5	2223	7869	632187	1,24473	0,1536
Sulfolobus solfataricus P2	Hyperthermophilic	85C	0,13	1,48	0,24	7	2977	8769	840528	1,04327	0,1757
Sulfolobus tokodaii str. 7	Hyperthermophilic	80C	0,12	1,58	0,46	10	2825	8452	759190	1,11329	0,1757
Synechococcus sp. PCC 7002	Mesophilic	38C	0,22	7,14	2,76	77	3186	66326	990621	6,6954	0,2084
Syntrophobacter fumaroxidans MPOB	Mesophilic	37C	0,23	8,12	3,72	139	4064	88146	1363806	6,46324	0,1779
Syntrophus aciditrophicus SB	Mesophilic	35C	0,21	6,19	1,93	60	3168	44509	940750	4,73123	0,1485
Thermoanaerobacter pseudethanolicus ATCC 33	Thermophilic	ND	0,16	2,62	0,94	18	2243	13955	681995	2,0462	0,2046
Thermoanaerobacter sp. X514	Thermophilic	60C	0,16	2,44	0,72	12	2588	13991	778470	1,79724	0,1769
Thermoanaerobacter tengcongensis MB4	Hyperthermophilic	75C	0,16	2,14	0,54	16	2349	13569	708708	1,91461	0,2265
Thermobifida fusca YX	Thermophilic	50-55C	0,3	15,37	7,01	190	3110	147391	1030353	14,3049	0,1667
Thermofilum pendens Hrk 5	Hyperthermophilic	88C	0,16	2,54	0,43	6	1876	10122	544101	1,86032	0,2079
Thermoplasma acidophilum DSM 1728	Thermophilic	59C	0,17	2,5	0,4	4	1482	9194	454297	2,02379	0,2084
Thermoplasma volcanium GSS1	Thermophilic	60C	0,16	2,51	0,53	5	1499	8602	450122	1,91104	0,2130
Thermosipho melanesiensis BI429	Thermophilic	70C	0,13	1,46	0,27	5	1879	6269	582244	1,0767	0,1795
Thermosynechococcus elongatus BP-1	Thermophilic	55C	0,21	6,28	1,7	35	2476	42738	774371	5,51906	0,1748
Thermotoga lettingae TMO	Hyperthermophilic	65C	0,14	1,58	0,29	6	2040	7941	668518	1,18785	0,1886
Thermotoga maritima MSB8	Hyperthermophilic	80C	0,16	2,57	0,75	14	1858	10780	584963	1,84285	0,2106
Thermotoga petrophila RKU-1	Hyperthermophilic	80C	0,16	2,21	0,45	8	1785	9849	574004	1,71584	0,2437
Thermus thermophilus HB27	Thermophilic	68C	0,21	5,24	1,31	25	2210	32545	670210	4,85594	0,1979
Thermus thermophilus HB8	Thermophilic	ND	0,21	5,62	1,79	34	2238	32262	667505	4,83322	0,1818
Thiobacillus denitrificans ATCC 25259	Mesophilic	28-32C	0,24	8,08	2,97	74	2827	61974	894776	6,9262	0,1639
Thiomicrospira crunogena XCL-2	Mesophilic	28-32C	0,23	6,56	1,87	30	2196	43222	725298	5,95921	0,2119
Treponema denticola ATCC 35405	Mesophilic	30-42C	0,16	3,39	1,08	28	2767	28899	866832	3,33386	0,0908
Tropheryma whipplei str. Twist	Mesophilic	37C	0,19	6,01	2,85	17	808	14324	265157	5,40208	0,1625
Vibrio cholerae O1 biovar eltor str. N16961	Mesophilic	20-30C	0,2	5,12	1,75	54	3835	53919	1166196	4,62349	0,1639
Vibrio cholerae O395	Mesophilic	20-30C	0,2	5,24	1,55	53	3875	57419	1207459	4,75536	0,1611
Vibrio harveyi ATCC BAA-1116	Mesophilic	20-30C	0,21	6,11	1,83	105	6055	101243	1726505	5,86404	0,1227
Vibrio parahaemolyticus RIMD 2210633	Mesophilic	20-30C	0,21	5,5	1,59	69	4832	80678	1492202	5,40664	0,1803
Vibrio vulnificus CMCP6	Mesophilic	20-30C	0,21	5,2	1,47	52	4484	71460	1446965	4,93861	0,1917
Vibrio vulnificus YJ016	Mesophilic	20-30C	0,2	5,39	1,89	87	5024	76204	1557222	4,89359	0,1704
Xanthomonas axonopodis pv. citri str. 306	Mesophilic	25-30C	0,26	10,55	4,02	160	4181	137418	1431661	9,5985	0,1718
Xanthomonas campestris pv. campestris str. 800	Mesophilic	25-30C	0,26	10,78	4,45	172	4273	140364	1450946	9,67396	0,1940
Xanthomonas campestris pv. vesicatoria str. 85-1	Mesophilic	25-30C	0,27	11,55	4,99	207	4726	162968	1572380	10,3644	0,1383
Xylella fastidiosa 9a5c	Mesophilic	26-28C	0,23	10,23	5,61	152	2832	63444	756399	8,38764	0,1936
Xylella fastidiosa Temecula1	Mesophilic	26-28C	0,23	7,88	2,26	47	2036	47824	654382	7,30827	0,2011
Yersinia enterocolitica subsp. enterocolitica 8081	Mesophilic	28-30C	0,21	5,47	1,36	46	4051	66910	1298267	5,15379	0,1764
Yersinia pestis CO92	Mesophilic	28-30C	0,21	5,6	1,48	51	4066	70950	1285246	5,52034	0,1572
Yersinia pestis KIM	Mesophilic	28-30C	0,21	6,03	1,9	70	4202	71939	1299885	5,53426	0,2211

Zymomonas mobilis subsp. mobilis ZM4	Mesophilic	25-30C	0,24	9,34	4,25	76	1998	48852	588985	8,29427	0,1908
