# Supplementary material for: Reduction in Structural Disorder and Functional Complexity in the Thermal Adaptation of Prokaryotes
Source: PLoS One. 2010 Aug 11;5(8):e12069. doi: 10.1371/journal.pone.0012069 (PMC2920320; doi:10.1371/journal.pone.0012069)
Supplement: Table S2 — Annotated TFs in Swiss-Prot in the four OGT groups psychrophiles, mesophiles, thermophiles and hyperthermophiles. (0.21 MB PDF) [file pone.0012069.s004.pdf]

SwissProt ID	Thermal category	protein length	Frequency of disordered residues
AADR_RHOPA	Mesophiles	239	0.0000
AAER_ECO57	Mesophiles	309	0.0032
ABGR_ECOLI	Mesophiles	302	0.0000
ACNR_CORDI	Mesophiles	190	0.0421
ACNR_COREF	Mesophiles	188	0.0691
ACNR_CORGL	Mesophiles	188	0.0904
ACOR_BACSU	Mesophiles	605	0.0149
ACOR_RALEH	Mesophiles	668	0.1003
ACRR_ECOL6	Mesophiles	215	0.0326
ADAA_BACSU	Mesophiles	211	0.1469
ADA_ECOLI	Mesophiles	354	0.0254
ADA_MYCTU	Mesophiles	496	0.0181
ADA_SALTY	Mesophiles	353	0.0000
ADCR_STRP6	Mesophiles	147	0.0068
ADHR_BACSU	Mesophiles	140	0.1500
ADIY_ECOLI	Mesophiles	253	0.0040
AGAR_ECO57	Mesophiles	269	0.1599
AGGR_ECOLX	Mesophiles	265	0.0000
AGMR_PSEAE	Mesophiles	221	0.1041
AHYR_AERHY	Mesophiles	260	0.0000
ALGB_PSEAE	Mesophiles	449	0.0423
ALLS_ECO57	Mesophiles	308	0.0032
ALLS_ECOK1	Mesophiles	308	0.0032
ALLS_ECOL5	Mesophiles	308	0.0032
ALLS_KLEPN	Mesophiles	270	0.0074
ALLS_SALTY	Mesophiles	308	0.0000
ALSR_BACSU	Mesophiles	302	0.0331
AMPR_PSEAE	Mesophiles	296	0.0000
AMPR_YEREN	Mesophiles	294	0.0000
ANR_PSEAE	Mesophiles	244	0.0041
APPY_ECOLI	Mesophiles	249	0.0000
ARAC_ECO57	Mesophiles	292	0.0000
ARAC_SALTY	Mesophiles	281	0.0036
ARAR_BACSU	Mesophiles	362	0.0525
ARGR1_BACC1	Mesophiles	149	0.0000
ARGR1_BACCR	Mesophiles	149	0.0000
ARGR1_CLOPE	Mesophiles	151	0.0000
ARGR1_LACPL	Mesophiles	153	0.0850
ARGR1_STRP1	Mesophiles	157	0.0127
ARGR2_BACC1	Mesophiles	149	0.0067
ARGR2_BACCR	Mesophiles	149	0.0067
ARGR2_CLOPE	Mesophiles	150	0.0000
ARGR2_LACPL	Mesophiles	152	0.0132
ARGR2_STRP1	Mesophiles	145	0.0069
ARGR2_STRP3	Mesophiles	145	0.0069
ARGR2_STRP6	Mesophiles	145	0.0069
ARGR_AERHH	Mesophiles	156	0.0128
ARGR_ANADE	Mesophiles	163	0.1043
ARGR_BACFN	Mesophiles	157	0.0000

ARGR_BACFR	Mesophiles	157	0.0000
ARGR_BACSU	Mesophiles	149	0.0201
ARGR_BIFAA	Mesophiles	172	0.1977
ARGR_BIFLD	Mesophiles	170	0.1176
ARGR_BIFLO	Mesophiles	170	0.1118
ARGR_CALS8	Thermophiles	152	0.0000
ARGR_CARHZ	Hyperthermophiles	150	0.0000
ARGR_CHLCV	Mesophiles	147	0.0000
ARGR_CHLTE	Thermophiles	149	0.0470
ARGR_CLOAB	Mesophiles	150	0.0000
ARGR_CLOB1	Mesophiles	150	0.0000
ARGR_CLOBA	Mesophiles	150	0.0000
ARGR_CLOBB	Mesophiles	150	0.0000
ARGR_CLOBK	Mesophiles	150	0.0000
ARGR_CLOBM	Mesophiles	150	0.0000
ARGR_CLOPH	Mesophiles	152	0.0000
ARGR_CLOTE	Mesophiles	153	0.0000
ARGR_CLOTH	Thermophiles	153	0.0000
ARGR_COLP3	Psychrophiles	157	0.0000
ARGR_CORDI	Mesophiles	163	0.1104
ARGR_COREF	Mesophiles	177	0.2147
ARGR_CORGB	Mesophiles	171	0.1754
ARGR_DEIRA	Mesophiles	157	0.0000
ARGR_DESRM	Mesophiles	152	0.0789
ARGR_ECO24	Mesophiles	156	0.0000
ARGR_ECO45	Mesophiles	156	0.0000
ARGR_ENTFA	Mesophiles	151	0.0199
ARGR_ENTS8	Mesophiles	156	0.0000
ARGR_GEOKA	Thermophiles	149	0.0067
ARGR_GEOTN	Thermophiles	149	0.0067
ARGR_HAEDU	Mesophiles	153	0.0000
ARGR_HAEI8	Mesophiles	151	0.0000
ARGR_HAEIE	Mesophiles	151	0.0000
ARGR_HAEIG	Mesophiles	151	0.0000
ARGR_HAEIN	Mesophiles	151	0.0000
ARGR_HAES1	Mesophiles	155	0.0000
ARGR_HAES2	Mesophiles	155	0.0000
ARGR_HELMI	Thermophiles	151	0.0993
ARGR_KLEP3	Mesophiles	156	0.0000
ARGR_LACDA	Mesophiles	157	0.0064
ARGR_LACLA	Mesophiles	152	0.0000
ARGR_LACLM	Mesophiles	152	0.0000
ARGR_LISIN	Mesophiles	149	0.0000
ARGR_LISMF	Mesophiles	149	0.0067
ARGR_MANSM	Mesophiles	155	0.0000
ARGR_MOOTA	Thermophiles	151	0.0000
ARGR_MYCA1	Mesophiles	164	0.0915
ARGR_MYCBO	Mesophiles	170	0.1235
ARGR_MYCLE	Mesophiles	167	0.1138
ARGR_MYCUA	Mesophiles	166	0.0542

ARGR_OCEIH	Mesophiles	149	0.0000
ARGR_PASMU	Mesophiles	155	0.0000
ARGR_PELLD	Mesophiles	148	0.0068
ARGR_PELTS	Thermophiles	151	0.0000
ARGR_PHOPR	Psychrophiles	156	0.0128
ARGR_PSYIN	Psychrophiles	150	0.0133
ARGR_RENSM	Mesophiles	179	0.0000
ARGR_SALTY	Mesophiles	156	0.0000
ARGR_SHEFN	Mesophiles	156	0.0192
ARGR_SHESH	Psychrophiles	156	0.0128
ARGR_SHEWM	Mesophiles	156	0.0000
ARGR_SOLUE	Mesophiles	145	0.0759
ARGR_STAA1	Mesophiles	150	0.0000
ARGR_STAEQ	Mesophiles	150	0.0000
ARGR_STAHJ	Mesophiles	150	0.0000
ARGR_STRAW	Mesophiles	178	0.2584
ARGR_STRCO	Mesophiles	179	0.2682
ARGR_STRGC	Mesophiles	156	0.0000
ARGR_STRMU	Mesophiles	145	0.0000
ARGR_STRPN	Mesophiles	148	0.0000
ARGR_STRSU	Mesophiles	157	0.0000
ARGR_THEMA	Hyperthermophiles	152	0.0000
ARGR_THEP3	Thermophiles	151	0.0000
ARGR_THET2	Thermophiles	164	0.0122
ARGR_THETN	Hyperthermophiles	151	0.0000
ARGR_VIBCH	Mesophiles	156	0.0000
ARGR_VIBHB	Mesophiles	156	0.0128
ARGR_VIBVU	Mesophiles	156	0.0128
ARGR_YERE8	Mesophiles	156	0.0064
ARGR_YERPA	Mesophiles	156	0.0064
ARSR1_ECOLX	Mesophiles	117	0.0000
ARSR2_ECOLX	Mesophiles	117	0.0000
ARSR_BACSU	Mesophiles	105	0.0000
ARSR_ECOLI	Mesophiles	117	0.0000
ARSR_STAAU	Mesophiles	104	0.0000
ASCG_ECOLI	Mesophiles	337	0.0119
ASNC_ECO57	Mesophiles	152	0.0000
ASNC_HAEIN	Mesophiles	150	0.0000
ATOC_ECOLI	Mesophiles	461	0.0152
AZLB_BACSU	Mesophiles	157	0.0000
BADR_RHOPA	Mesophiles	175	0.0629
BBUR_BORBR	Mesophiles	325	0.0185
BCNA_CLOPE	Mesophiles	185	0.0000
BETI1_CHRSD	Mesophiles	202	0.0446
BETI2_CHRSD	Mesophiles	207	0.0193
BETI_BRUA2	Mesophiles	198	0.0051
BETI_BRUME	Mesophiles	198	0.0051
BETI_BRUSI	Mesophiles	198	0.0051
BETI_BRUSU	Mesophiles	198	0.0051
BETI_BURM1	Mesophiles	194	0.0309

BETI_BURTA	Mesophiles	195	0.0000
BETI_BURXL	Mesophiles	195	0.0051
BETI_ECO24	Mesophiles	195	0.0051
BETI_ECO45	Mesophiles	195	0.0051
BETI_ECO57	Mesophiles	195	0.0051
BETI_ECO7I	Mesophiles	195	0.0051
BETI_ECO8A	Mesophiles	195	0.0051
BETI_ECODH	Mesophiles	195	0.0051
BETI_ECOL5	Mesophiles	195	0.0051
BETI_ECOL6	Mesophiles	195	0.0051
BETI_ECOLU	Mesophiles	195	0.0051
BETI_ECOSM	Mesophiles	195	0.0051
BETI_ENTS8	Mesophiles	202	0.0594
BETI_KLEP3	Mesophiles	195	0.0103
BETI_PSEA7	Mesophiles	197	0.0102
BETI_PSEF5	Mesophiles	197	0.0102
BETI_PSEPF	Mesophiles	197	0.0102
BETI_VIBHB	Mesophiles	199	0.0101
BETI_VIBPA	Mesophiles	199	0.0101
BETI_VIBVU	Mesophiles	198	0.0101
BETI_VIBVY	Mesophiles	198	0.0152
BETI_YERPA	Mesophiles	198	0.0101
BGLJ_ECOLI	Mesophiles	175	0.0000
BIGR_XYLFA	Mesophiles	114	0.1930
BIGR_XYLFT	Mesophiles	114	0.1930
BKDR_PSEAE	Mesophiles	153	0.0000
BLTR_BACSU	Mesophiles	273	0.0000
BMRR_BACSU	Mesophiles	278	0.0000
BPHR_BURXL	Mesophiles	245	0.0653
BSCR_BACSU	Mesophiles	194	0.0000
BSDA_BACSU	Mesophiles	290	0.0000
BTR_BORPE	Mesophiles	242	0.0083
BVGA_BORBR	Mesophiles	209	0.0000
CADC_LACLA	Mesophiles	119	0.0000
CADC_LISMO	Mesophiles	119	0.0000
CADC_STAAU	Mesophiles	122	0.0000
CADF_STAAU	Mesophiles	121	0.0083
CAF1R_YERPE	Mesophiles	301	0.0000
CARQ_MYXXA	Mesophiles	174	0.1724
CBBR_RHORU	Mesophiles	298	0.0034
CBBR_RHOSH	Mesophiles	310	0.0258
CBBR_SINMW	Mesophiles	313	0.0064
CBIO2_MYCS5	Mesophiles	311	0.0193
CBL_ECOLI	Mesophiles	316	0.0000
CCPA_BACSU	Mesophiles	334	0.0299
CCPA_STAAC	Mesophiles	329	0.0304
CCPA_STAAR	Mesophiles	329	0.0213
CCPA_STAEQ	Mesophiles	329	0.0669
CCPA_STRMU	Mesophiles	333	0.0330
CCPB_BACSU	Mesophiles	311	0.0000

CCPN_BACSU	Mesophiles	212	0.0000
CFAD_ECOLX	Mesophiles	265	0.0000
CFXR_RALEH	Mesophiles	317	0.0063
CHBR_ECOLI	Mesophiles	280	0.0000
CITR_BACSU	Mesophiles	291	0.0000
CLP_XANCP	Mesophiles	230	0.0957
CMPA_BACSU	Mesophiles	214	0.0421
CMTR_MYCBO	Mesophiles	118	0.0085
CNRH_RALME	Mesophiles	191	0.0524
CRP_ECO57	Mesophiles	210	0.0238
CRP_HAEIN	Mesophiles	224	0.0714
CRP_PASMU	Mesophiles	209	0.0191
CRP_SALTY	Mesophiles	210	0.0238
CSCR_ECOLX	Mesophiles	331	0.0060
CSGD_ECOLI	Mesophiles	216	0.0000
CSGD_SALTY	Mesophiles	216	0.0000
CSVR_ECOLX	Mesophiles	301	0.0033
CUER_BACSU	Mesophiles	143	0.0070
CUER_ECO57	Mesophiles	135	0.1185
CUER_ECOL6	Mesophiles	135	0.1333
CUER_ECOLI	Mesophiles	135	0.1185
CUER_SALTY	Mesophiles	138	0.0580
CUER_VIBC3	Mesophiles	139	0.0432
CUER_YERPE	Mesophiles	139	0.0000
CYNR_ECO57	Mesophiles	299	0.0100
CYNR_ECOLI	Mesophiles	299	0.0000
CYSB_ECO57	Mesophiles	324	0.0000
CYSB_HAEIN	Mesophiles	323	0.0000
CYSB_KLEPN	Mesophiles	324	0.0031
CYSB_SALTY	Mesophiles	324	0.0000
CYSL_BACSU	Mesophiles	299	0.0134
CYTR_ECO57	Mesophiles	341	0.0674
CZCR_BACC1	Mesophiles	288	0.0000
CZCR_BACCR	Mesophiles	288	0.0000
CZCR_BACSU	Mesophiles	288	0.0000
DASR_STRAW	Mesophiles	254	0.1850
DASR_STRCO	Mesophiles	254	0.2047
DCTD_RHILE	Mesophiles	448	0.1004
DCTR_BACSU	Mesophiles	226	0.0044
DCTR_ECO57	Mesophiles	176	0.0000
DCTR_ECOL6	Mesophiles	176	0.0000
DCTR_ECOLI	Mesophiles	176	0.0000
DCTR_SHIFL	Mesophiles	176	0.0000
DEGA_BACSU	Mesophiles	337	0.1157
DEGU_BACSU	Mesophiles	229	0.0218
DEOR_ECO57	Mesophiles	252	0.0040
DESR_BACSU	Mesophiles	199	0.0050
DGOR_ECOLI	Mesophiles	229	0.0742
DHAR_ECOLI	Mesophiles	639	0.0203
DHAS_LACLA	Mesophiles	187	0.0000

DSDC_ECOLI	Mesophiles	311	0.0000
DTXR_CORDI	Mesophiles	226	0.1283
DTXR_COREF	Mesophiles	230	0.1348
DTXR_CORGL	Mesophiles	228	0.1491
EBGR_ECOLI	Mesophiles	327	0.0061
ENVR_ECO57	Mesophiles	220	0.0091
ENVY_ECOLI	Mesophiles	253	0.0040
EUTR_ECOLI	Mesophiles	350	0.0171
EUTR_SALTY	Mesophiles	350	0.0171
EVGA_ECO57	Mesophiles	204	0.0000
EXSA_PSEAE	Mesophiles	278	0.0468
EXUR_BACSU	Mesophiles	153	0.0065
EXUR_ECO57	Mesophiles	258	0.0465
FABR_ECO24	Mesophiles	215	0.0419
FABR_ECOHS	Mesophiles	215	0.0419
FABR_ECOK1	Mesophiles	215	0.0558
FABR_ENTS8	Mesophiles	211	0.0474
FABR_KLEP7	Mesophiles	210	0.0429
FABR_SALTY	Mesophiles	210	0.0381
FABR_SHIB3	Mesophiles	215	0.0372
FABR_YERE8	Mesophiles	210	0.0190
FABR_YERPA	Mesophiles	211	0.0190
FADR_BACSU	Mesophiles	194	0.0000
FADR_ECO24	Mesophiles	239	0.0084
FADR_ENTS8	Mesophiles	239	0.0042
FADR_HAEI8	Mesophiles	241	0.0166
FADR_HAEIE	Mesophiles	241	0.0166
FADR_HAEIG	Mesophiles	241	0.0166
FADR_KLEP3	Mesophiles	239	0.0000
FADR_KLEP7	Mesophiles	239	0.0000
FADR_PASMU	Mesophiles	241	0.0373
FADR_SALTY	Mesophiles	239	0.0084
FADR_SHEAM	Mesophiles	240	0.0208
FADR_SHEDO	Mesophiles	239	0.0042
FADR_SHEFN	Mesophiles	239	0.0209
FADR_VIBC3	Mesophiles	279	0.0143
FADR_VIBPA	Mesophiles	279	0.0215
FADR_VIBVU	Mesophiles	279	0.0287
FADR_YERE8	Mesophiles	239	0.0126
FADR_YERPA	Mesophiles	239	0.0084
FAPR_BACC1	Mesophiles	197	0.0558
FAPR_BACCN	Mesophiles	197	0.0508
FAPR_BACCR	Mesophiles	197	0.0558
FAPR_BACSU	Mesophiles	188	0.0106
FAPR_ECOLX	Mesophiles	260	0.0000
FAPR_GEOKA	Thermophiles	198	0.0556
FAPR_GEOTN	Thermophiles	198	0.0707
FAPR_LISIN	Mesophiles	189	0.0053
FAPR_LISMF	Mesophiles	189	0.0053
FAPR_OCEIH	Mesophiles	191	0.0052

FAPR_STAA1	Mesophiles	185	0.0108
FAPR_STAEQ	Mesophiles	186	0.0000
FAPR_STAHJ	Mesophiles	186	0.0000
FAPR_THETN	Hyperthermophiles	200	0.0000
FEAR_ECOLI	Mesophiles	301	0.0100
FECI_ECOLI	Mesophiles	173	0.0520
FHLA_ECOLI	Mesophiles	692	0.0101
FHLA_SALTY	Mesophiles	692	0.0434
FIMW_SALTY	Mesophiles	198	0.0000
FIMZ_ECO57	Mesophiles	210	0.0000
FIMZ_SALTY	Mesophiles	210	0.0286
FISL_NEIMA	Mesophiles	79	0.0000
FISL_PSEAE	Mesophiles	104	0.4808
FISL_XANAC	Mesophiles	90	0.2556
FISL_XANCP	Mesophiles	90	0.2556
FISL_XYLFA	Mesophiles	90	0.2000
FISL_XYLFT	Mesophiles	90	0.2000
FIS_ACTSZ	Mesophiles	99	0.0404
FIS_AERHH	Mesophiles	98	0.2041
FIS_ECO24	Mesophiles	98	0.1020
FIS_HAEDU	Mesophiles	98	0.3469
FIS_HAEI8	Mesophiles	99	0.1111
FIS_HAEIE	Mesophiles	99	0.0505
FIS_HAES1	Mesophiles	98	0.1429
FIS_KLEPN	Mesophiles	93	0.1075
FIS_MANSM	Mesophiles	99	0.2424
FIS_PASMU	Mesophiles	99	0.2828
FIS_PHOPR	Psychrophiles	98	0.0918
FIS_PSYIN	Psychrophiles	99	0.1818
FIS_SHEAM	Mesophiles	101	0.2079
FIS_SHEDO	Mesophiles	101	0.1089
FIS_SHEFN	Mesophiles	101	0.0891
FIS_SHESH	Psychrophiles	101	0.2475
FIS_VIBC3	Mesophiles	98	0.0714
FIS_YERE8	Mesophiles	98	0.0510
FIXJ_BRAJA	Mesophiles	205	0.0000
FIXK_BRAJA	Mesophiles	237	0.0000
FLBD_CAUCR	Mesophiles	455	0.0703
FLHD_BORBR	Mesophiles	107	0.0000
FLHD_BORPE	Mesophiles	107	0.0000
FLHD_ECO24	Mesophiles	116	0.0603
FLHD_ECO45	Mesophiles	119	0.0588
FLHD_ECO57	Mesophiles	116	0.0517
FLHD_ENTS8	Mesophiles	119	0.0504
FLHD_RALEH	Mesophiles	105	0.0000
FLHD_RALEJ	Mesophiles	105	0.0000
FLHD_SALTY	Mesophiles	113	0.0177
FLHD_SHIBS	Mesophiles	119	0.0588
FLHD_YEREN	Mesophiles	119	0.0168
FLIA_ECO57	Mesophiles	239	0.0544

FLIA_PSEAE	Mesophiles	247	0.0567
FLIA_SALTY	Mesophiles	239	0.1213
FLIA_YEREN	Mesophiles	230	0.1435
FLJA_SALTY	Mesophiles	179	0.0000
FLP_LACCA	Mesophiles	219	0.0000
FNRL_RHOS4	Mesophiles	248	0.0161
FNRN_RHILV	Mesophiles	240	0.0042
FNR_BACSU	Mesophiles	238	0.0000
FNR_ECO57	Mesophiles	250	0.0080
FNR_HAEIN	Mesophiles	257	0.0039
FNR_PASMU	Mesophiles	273	0.0000
FNR_SALTY	Mesophiles	250	0.0080
FNR_SHIDY	Mesophiles	250	0.0080
FNR_VIBC3	Mesophiles	250	0.0080
FNR_YERPE	Mesophiles	250	0.0120
FRLR_ECO57	Mesophiles	243	0.0000
FRLR_ECOLI	Mesophiles	243	0.0000
FRLR_SHIFL	Mesophiles	243	0.0000
FRUR_BACSU	Mesophiles	251	0.0000
FRUR_ECO57	Mesophiles	334	0.0000
FRUR_SALTY	Mesophiles	334	0.0000
FUCR_ECOLI	Mesophiles	243	0.0329
FUCR_HAEIN	Mesophiles	249	0.0000
FURH_ARCFU	Hyperthermophiles	128	0.0000
FUR_BACSU	Mesophiles	149	0.0000
FUR_BORBR	Mesophiles	139	0.0000
FUR_BRUA2	Mesophiles	141	0.1844
FUR_BRUME	Mesophiles	141	0.1844
FUR_BRUSU	Mesophiles	141	0.1773
FUR_ECO57	Mesophiles	148	0.0676
FUR_HAEDU	Mesophiles	149	0.0201
FUR_HAEIN	Mesophiles	146	0.0000
FUR_HELPJ	Mesophiles	150	0.0400
FUR_HELPY	Mesophiles	150	0.0400
FUR_KLEPN	Mesophiles	155	0.0968
FUR_MYCBO	Mesophiles	147	0.0136
FUR_NEIGO	Mesophiles	144	0.0000
FUR_NEIMA	Mesophiles	144	0.0000
FUR_NEIMC	Mesophiles	144	0.0000
FUR_PSEAE	Mesophiles	134	0.0000
FUR_PSEFL	Mesophiles	123	0.0325
FUR_RALME	Mesophiles	143	0.0280
FUR_RHILV	Mesophiles	142	0.0845
FUR_SHIFL	Mesophiles	148	0.0676
FUR_STAA8	Mesophiles	136	0.0000
FUR_STAEP	Mesophiles	138	0.0000
FUR_STAEQ	Mesophiles	139	0.0000
FUR_VIBC3	Mesophiles	150	0.0867
FUR_VIBCH	Mesophiles	150	0.0867
FUR_VIBPA	Mesophiles	149	0.0671

FUR_VIBVU	Mesophiles	149	0.0671
FUR_YERPE	Mesophiles	148	0.0676
GABR_BACSU	Mesophiles	479	0.0063
GACA_PSEAE	Mesophiles	214	0.0000
GACA_PSEFL	Mesophiles	213	0.0000
GADE_ECO57	Mesophiles	175	0.0000
GADW_ECO57	Mesophiles	242	0.0000
GADW_ECOL6	Mesophiles	242	0.0000
GADX_ECO27	Mesophiles	274	0.0000
GADX_ECO57	Mesophiles	274	0.0109
GADX_ECOL6	Mesophiles	274	0.0000
GADX_ECOLI	Mesophiles	274	0.0073
GADX_SHIFL	Mesophiles	274	0.0146
GALR_ECOLI	Mesophiles	343	0.0758
GALR_HAEIN	Mesophiles	332	0.0000
GALR_LACCA	Mesophiles	331	0.0151
GALR_SALTY	Mesophiles	342	0.0965
GALR_STRTR	Thermophiles	331	0.0030
GALS_ECOLI	Mesophiles	346	0.0260
GALS_SALTY	Mesophiles	340	0.0059
GATR_ECOLI	Mesophiles	259	0.0425
GCVA_ECO57	Mesophiles	305	0.0000
GCVA_HAEIN	Mesophiles	301	0.0000
GERE_BACSU	Mesophiles	74	0.1486
GLCC_ECOL6	Mesophiles	254	0.0039
GLCR_BACSU	Mesophiles	258	0.0039
GLK_BURTA	Mesophiles	641	0.0328
GLK_BURXL	Mesophiles	638	0.0392
GLNR_BACCE	Mesophiles	129	0.0698
GLNR_BACSU	Mesophiles	135	0.2074
GLPR_ECOLI	Mesophiles	252	0.1389
GLPR_HAEIN	Mesophiles	255	0.0235
GLPR_PSEAE	Mesophiles	251	0.0438
GLTC_BACSU	Mesophiles	300	0.0000
GLTR_BACSU	Mesophiles	296	0.0473
GLVR_BACSU	Mesophiles	254	0.0079
GMUR_BACSU	Mesophiles	237	0.0042
GNTR_BACSU	Mesophiles	243	0.0000
GNTR_ECOL6	Mesophiles	331	0.0000
GRP_ZYMMO	Mesophiles	164	0.0183
HCAR_ECOLI	Mesophiles	296	0.0000
HDFR_ECO24	Mesophiles	279	0.0000
HDFR_ECO45	Mesophiles	279	0.0000
HDFR_ECO7I	Mesophiles	279	0.0000
HDFR_ECODH	Mesophiles	279	0.0000
HDFR_ECOLU	Mesophiles	279	0.0000
HDFR_SALTY	Mesophiles	278	0.0000
HDFR_SHIB3	Mesophiles	279	0.0000
HDFR_YERE8	Mesophiles	293	0.0751
HDFR_YERPA	Mesophiles	293	0.0819

HEXR_ECOLI	Mesophiles	289	0.0173
HEXR_PSEAE	Mesophiles	285	0.0105
HILD_SALTY	Mesophiles	309	0.0194
HLYU_VIBCH	Mesophiles	108	0.0000
HMMR_RHILV	Mesophiles	129	0.0620
HMRR_SINMW	Mesophiles	147	0.0680
HOSA_ECO11	Mesophiles	135	0.0519
HOSA_ECO27	Mesophiles	135	0.0074
HOSA_ECO57	Mesophiles	135	0.0074
HOXA_BRAJA	Mesophiles	485	0.0454
HOXA_RALEH	Mesophiles	482	0.0249
HPCR_ECOLX	Mesophiles	148	0.0541
HPR_BACC1	Mesophiles	185	0.0000
HPR_BACCN	Mesophiles	185	0.0000
HPR_BACCR	Mesophiles	185	0.0000
HPR_BACSU	Mesophiles	203	0.0936
HPR_GEOKA	Thermophiles	201	0.0945
HPR_GEOTN	Thermophiles	200	0.0900
HRCA_KINRD	Mesophiles	346	0.0347
HRCA_LACC3	Mesophiles	348	0.0144
HRCA_LACCB	Mesophiles	348	0.0144
HRCA_MYCPE	Mesophiles	338	0.0000
HRCA_PEDPA	Mesophiles	346	0.0347
HRCA_ROSCS	Thermophiles	359	0.0334
HRCA_STRGC	Mesophiles	344	0.0029
HRCA_STRP2	Mesophiles	344	0.0087
HRCA_STRP4	Mesophiles	344	0.0087
HRCA_STRPI	Mesophiles	344	0.0087
HRDA_STRCO	Mesophiles	396	0.2601
HRDB_STRCO	Mesophiles	511	0.4481
HRDC_STRCO	Mesophiles	339	0.3097
HRDD_STRCO	Mesophiles	332	0.1988
HSPR_STRCO	Mesophiles	151	0.1258
HUTC_PSEAE	Mesophiles	250	0.0840
HYFR_ECOLI	Mesophiles	670	0.0463
ICAR_STAA8	Mesophiles	186	0.0000
ICAR_STAEP	Mesophiles	185	0.0000
ICAR_STAEQ	Mesophiles	185	0.0000
ICIA_ECO24	Mesophiles	297	0.0202
ICIA_ECO7I	Mesophiles	297	0.0202
ICIA_ENTS8	Mesophiles	297	0.0404
ICIA_KLEP3	Mesophiles	297	0.0236
ICIA_KLEP7	Mesophiles	297	0.0236
ICIA_PHOPR	Psychrophiles	298	0.0034
ICIA_PSEAB	Mesophiles	300	0.0100
ICIA_PSEF5	Mesophiles	299	0.0000
ICIA_PSEPF	Mesophiles	297	0.0067
ICIA_SALTY	Mesophiles	297	0.0269
ICIA_VIBC3	Mesophiles	298	0.0000
ICIA_VIBCH	Mesophiles	298	0.0000

ICIA_VIBPA	Mesophiles	298	0.0034
ICIA_VIBVU	Mesophiles	298	0.0000
ICIA_VIBVY	Mesophiles	298	0.0000
ICIA_YERE8	Mesophiles	297	0.0236
ICIA_YERPA	Mesophiles	302	0.0066
IDER_MYCBO	Mesophiles	230	0.2696
IDER_MYCLE	Mesophiles	230	0.1565
IDNR_ECOLI	Mesophiles	332	0.0000
IF2B_AERPE	Hyperthermophiles	148	0.0068
IF2B_METS3	Mesophiles	135	0.0000
IF2B_METTP	Thermophiles	208	0.0000
IF2B_SULSO	Hyperthermophiles	139	0.0000
ILVR_CAUCR	Mesophiles	296	0.0034
ILVY_ECOLI	Mesophiles	297	0.0236
ILVY_HAEIN	Mesophiles	292	0.0479
ILVY_SALTY	Mesophiles	295	0.0237
INVF_SALTY	Mesophiles	216	0.0000
IOLR_BACSU	Mesophiles	251	0.0000
IRGB_VIBC3	Mesophiles	298	0.0067
IRGB_VIBCH	Mesophiles	298	0.0067
ISCR_ECO24	Mesophiles	162	0.1358
ISCR_ECO8A	Mesophiles	162	0.0926
ISCR_ENTS8	Mesophiles	162	0.1235
ISCR_KLEP3	Mesophiles	163	0.1227
ISCR_KLEP7	Mesophiles	163	0.1227
ISCR_PHOPR	Psychrophiles	172	0.1163
ISCR_SALTY	Mesophiles	164	0.1159
ISCR_VIBCH	Mesophiles	173	0.0000
ISCR_VIBHB	Mesophiles	168	0.0119
ISCR_VIBPA	Mesophiles	168	0.0119
ISCR_VIBVU	Mesophiles	168	0.0000
ISCR_YERE8	Mesophiles	164	0.1646
ISCR_YERPA	Mesophiles	164	0.1646
KDGR_BACSU	Mesophiles	339	0.0501
LACI_ECOLI	Mesophiles	360	0.0306
LACI_KLEPN	Mesophiles	354	0.0424
LACR_LACLA	Mesophiles	255	0.0039
LACR_STAA8	Mesophiles	251	0.0159
LACR_STAAM	Mesophiles	251	0.0159
LACR_STAAR	Mesophiles	251	0.0159
LACR_STAEQ	Mesophiles	251	0.0080
LACR_STRMU	Mesophiles	251	0.0478
LAFS_VIBPA	Mesophiles	242	0.2479
LASR_PSEAE	Mesophiles	239	0.0000
LCRF_YERPE	Mesophiles	271	0.0000
LEU11_SULSO	Hyperthermophiles	461	0.0000
LEU12_SULTO	Hyperthermophiles	460	0.0000
LEUO_ECOLI	Mesophiles	314	0.0478
LEUO_SALTY	Mesophiles	314	0.0382
LIAR_BACSU	Mesophiles	211	0.0047

LLDR_ECOLI	Mesophiles	258	0.0930
LMRA_BACSU	Mesophiles	188	0.0000
LRHA_ECO57	Mesophiles	312	0.0128
LRHA_ECOLI	Mesophiles	312	0.0256
LRPA_BACSU	Mesophiles	136	0.0000
LRPB_BACSU	Mesophiles	149	0.0000
LRPC_BACSU	Mesophiles	144	0.0000
LRP_ECO57	Mesophiles	164	0.0366
LRP_HAEIN	Mesophiles	166	0.0241
LRP_KLEPN	Mesophiles	164	0.0366
LRP_SALTY	Mesophiles	164	0.0305
LTRA_KLEPN	Mesophiles	309	0.0485
LUXO_VIBC3	Mesophiles	530	0.0434
LUXO_VIBCH	Mesophiles	455	0.0308
LUXO_VIBHA	Mesophiles	453	0.0486
LUXO_VIBPA	Mesophiles	453	0.0574
LUXO_VIBVU	Mesophiles	453	0.0309
LUXR_VIBHA	Mesophiles	205	0.0098
LYSG_COREF	Mesophiles	290	0.0000
LYSG_CORGL	Mesophiles	290	0.0103
LYSM_SULSO	Hyperthermophiles	142	0.0000
LYSM_SULTO	Hyperthermophiles	140	0.0000
LYSR_ECOLI	Mesophiles	311	0.0129
MALI_ECOLI	Mesophiles	342	0.0029
MALR_STRCO	Mesophiles	344	0.0959
MALR_STRPN	Mesophiles	328	0.0366
MALT_ECO24	Mesophiles	421	0.0024
MALT_ECO45	Mesophiles	421	0.0024
MALT_ECODH	Mesophiles	421	0.0024
MALT_ECOL6	Mesophiles	421	0.0024
MALT_ECOLC	Mesophiles	421	0.0024
MALT_ENTS8	Mesophiles	421	0.0024
MALT_KLEP3	Mesophiles	421	0.0071
MALT_KLEP7	Mesophiles	421	0.0071
MALT_SALTY	Mesophiles	421	0.0048
MALT_SHIF8	Mesophiles	421	0.0024
MALT_SHISS	Mesophiles	421	0.0024
MALT_VIBCH	Mesophiles	422	0.0024
MALT_VIBHB	Mesophiles	422	0.0166
MALT_VIBPA	Mesophiles	422	0.0095
MALT_VIBVU	Mesophiles	422	0.0024
MALT_VIBVY	Mesophiles	422	0.0024
MALT_YERE8	Mesophiles	423	0.0024
MALT_YERPA	Mesophiles	423	0.0024
MARA_ECO57	Mesophiles	127	0.0315
MARA_SALTY	Mesophiles	127	0.0079
MARR_ECOLI	Mesophiles	144	0.0000
MARR_SALTY	Mesophiles	144	0.0000
MATA_ECO24	Mesophiles	196	0.0000
MATA_ECO55	Mesophiles	196	0.0000

MATA_ECO57	Mesophiles	196	0.0000
MATA_ECO81	Mesophiles	196	0.0000
MATA_ECODH	Mesophiles	196	0.0000
MATA_ECOK1	Mesophiles	196	0.0000
MATA_ECOLU	Mesophiles	196	0.0000
MATA_KLEP3	Mesophiles	180	0.0000
MATA_KLEP7	Mesophiles	180	0.0000
MAUR_KLEPN	Mesophiles	308	0.0000
MAUR_PARDE	Mesophiles	283	0.0000
MCBR_ECOLI	Mesophiles	221	0.0045
MELR_ECOL6	Mesophiles	302	0.0298
MERD_PSEAE	Mesophiles	121	0.0661
MERD_PSEFL	Mesophiles	55	0.0000
MERD_SHIFL	Mesophiles	120	0.0333
MERR_BACCE	Mesophiles	132	0.0076
MERR_PSEAE	Mesophiles	144	0.0139
MERR_SHIFL	Mesophiles	144	0.0069
MERR_STAAU	Mesophiles	135	0.0074
METJ_ACTSZ	Mesophiles	105	0.1238
METJ_ECO24	Mesophiles	105	0.2571
METJ_ENTS8	Mesophiles	105	0.2667
METJ_HAEDU	Mesophiles	105	0.1429
METJ_HAEI8	Mesophiles	105	0.1524
METJ_HAEIE	Mesophiles	105	0.1524
METJ_HAES1	Mesophiles	105	0.1524
METJ_KLEP3	Mesophiles	105	0.2381
METJ_KLEP7	Mesophiles	105	0.2095
METJ_PASMU	Mesophiles	105	0.1905
METJ_SALTY	Mesophiles	105	0.2190
METJ_VIBC3	Mesophiles	105	0.2476
METJ_VIBHB	Mesophiles	106	0.2547
METJ_VIBVU	Mesophiles	105	0.2857
METJ_YERE8	Mesophiles	105	0.2190
METJ_YERPA	Mesophiles	105	0.1905
METR_ECO57	Mesophiles	317	0.0505
METR_HAEIN	Mesophiles	309	0.0000
METR_SALTY	Mesophiles	317	0.0442
MEXR_PSEAE	Mesophiles	147	0.0408
MFD_BACSU	Mesophiles	1177	0.0229
MFD_ECOLI	Mesophiles	1148	0.0200
MFD_HAEIN	Mesophiles	1146	0.0297
MFD_HELPJ	Mesophiles	1001	0.0130
MFD_HELPY	Mesophiles	999	0.0110
MFD_MYCBO	Mesophiles	1234	0.0916
MFD_MYXXA	Mesophiles	61	0.0000
MFD_STAA3	Mesophiles	1168	0.0265
MFD_STAAB	Mesophiles	1168	0.0265
MFD_STAAC	Mesophiles	1168	0.0265
MFD_STAAM	Mesophiles	1168	0.0265
MFD_STAAR	Mesophiles	1168	0.0240

MFD_STAAS	Mesophiles	1168	0.0265
MFD_STAEQ	Mesophiles	1169	0.0359
MFD_STAES	Mesophiles	1169	0.0359
MFD_STAHJ	Mesophiles	1169	0.0376
MGRA_STAA8	Mesophiles	147	0.0136
MHQR_BACSU	Mesophiles	145	0.0000
MLER_LACLA	Mesophiles	291	0.0000
MLRA_ECOLI	Mesophiles	243	0.0000
MLRA_SALTY	Mesophiles	243	0.0000
MMSR_PSEAE	Mesophiles	307	0.0228
MNGR_ECOLI	Mesophiles	240	0.0292
MNTR_BACC0	Mesophiles	142	0.0704
MNTR_BACC2	Mesophiles	142	0.0563
MNTR_BACC4	Mesophiles	142	0.0563
MNTR_BACCN	Mesophiles	142	0.0211
MNTR_BACCR	Mesophiles	142	0.0704
MNTR_BACSU	Mesophiles	142	0.0915
MNTR_ECO57	Mesophiles	155	0.1419
MNTR_ECOL6	Mesophiles	155	0.1097
MNTR_GEOKA	Thermophiles	141	0.0567
MNTR_GEOTN	Thermophiles	141	0.0496
MNTR_LISIN	Mesophiles	142	0.0986
MNTR_LISMF	Mesophiles	142	0.0915
MNTR_OCEIH	Mesophiles	147	0.0476
MNTR_SALTY	Mesophiles	157	0.1210
MODE_ECO57	Mesophiles	262	0.0000
MODE_HAEIN	Mesophiles	255	0.0510
MODE_YERPE	Mesophiles	263	0.0114
MOXX_PARDE	Mesophiles	223	0.0000
MPRA_ECO57	Mesophiles	176	0.0682
MPRR1_STRCO	Mesophiles	316	0.0791
MPRR2_STRCO	Mesophiles	328	0.0518
MSMR_BACSU	Mesophiles	344	0.0291
MSMR_STRMU	Mesophiles	278	0.0144
MTA_BACSU	Mesophiles	257	0.0078
MTE8_ECOLX	Mesophiles	417	0.0552
MTRR_NEIGO	Mesophiles	210	0.0095
MXIE_SHIFL	Mesophiles	210	0.0000
NAC_ECOLI	Mesophiles	305	0.0000
NAGC_ECO57	Mesophiles	406	0.0074
NANR_ECO45	Mesophiles	263	0.2167
NANR_ECO57	Mesophiles	263	0.1863
NANR_ECO7I	Mesophiles	263	0.1787
NANR_ECO8A	Mesophiles	263	0.1825
NANR_ECOLU	Mesophiles	263	0.1749
NANR_SALTY	Mesophiles	263	0.1635
NANR_SHIDS	Mesophiles	263	0.1483
NANR_SHISS	Mesophiles	263	0.1825
NARL_ECO57	Mesophiles	216	0.0417
NARP_ECOLI	Mesophiles	215	0.0000

NARP_HAEIN	Mesophiles	208	0.0048
NFXB_PSEAE	Mesophiles	187	0.0053
NHAR_ECOLI	Mesophiles	301	0.0000
NHAR_VIBCH	Mesophiles	296	0.0068
NIFA_BRAJA	Mesophiles	582	0.1289
NIFA_KLEPN	Mesophiles	524	0.0706
NIFA_RHILE	Mesophiles	519	0.0559
NIFA_RHILT	Mesophiles	353	0.0255
NIKR1_METAC	Mesophiles	140	0.0000
NIKR1_METMA	Mesophiles	146	0.0274
NIKR2_METAC	Mesophiles	140	0.0357
NIKR2_METMA	Mesophiles	140	0.0000
NIKR3_METAC	Mesophiles	140	0.0000
NIKR3_METMA	Mesophiles	140	0.0286
NIKR_ANADE	Mesophiles	139	0.0000
NIKR_ARCFU	Hyperthermophiles	138	0.0000
NIKR_BRAJA	Mesophiles	148	0.1622
NIKR_BRUA1	Mesophiles	132	0.1515
NIKR_BRUME	Mesophiles	132	0.1515
NIKR_CAMHC	Mesophiles	138	0.0072
NIKR_CHLTE	Thermophiles	134	0.0000
NIKR_DESDG	Mesophiles	139	0.0432
NIKR_DESPS	Psychrophiles	136	0.0221
NIKR_DESVH	Mesophiles	139	0.0000
NIKR_DESVM	Mesophiles	139	0.0000
NIKR_ECO24	Mesophiles	133	0.0827
NIKR_ECOL5	Mesophiles	133	0.0526
NIKR_ECOSE	Mesophiles	133	0.0827
NIKR_ECOSM	Mesophiles	133	0.0677
NIKR_GEOMG	Mesophiles	139	0.0791
NIKR_GEOSL	Mesophiles	139	0.0288
NIKR_HALMA	Mesophiles	139	0.0216
NIKR_HALS3	Thermophiles	142	0.0141
NIKR_HELAH	Mesophiles	148	0.0203
NIKR_HELHP	Mesophiles	143	0.0350
NIKR_HELP2	Mesophiles	148	0.0068
NIKR_HELPG	Mesophiles	148	0.0473
NIKR_HELPH	Mesophiles	148	0.0203
NIKR_HELPJ	Mesophiles	148	0.0135
NIKR_HELPS	Mesophiles	148	0.0338
NIKR_HELPY	Mesophiles	148	0.0270
NIKR_KLEP3	Mesophiles	132	0.1364
NIKR_KLEP7	Mesophiles	132	0.1439
NIKR_META3	Mesophiles	0	0.0000
NIKR_METBU	Mesophiles	140	0.0000
NIKR_METKA	Hyperthermophiles	141	0.0071
NIKR_METTP	Thermophiles	140	0.0000
NIKR_PELPD	Mesophiles	139	0.0000
NIKR_PYRAB	Hyperthermophiles	138	0.0000
NIKR_PYRFU	Hyperthermophiles	138	0.0000

NIKR_PYRHO	Hyperthermophiles	138	0.0000
NIKR_RHOP2	Mesophiles	139	0.1511
NIKR_RHOPA	Mesophiles	148	0.2432
NIKR_RHOPS	Mesophiles	140	0.1929
NIKR_RHOPT	Mesophiles	147	0.2313
NIKR_SALTY	Mesophiles	133	0.0677
NIKR_SOLUE	Mesophiles	139	0.0216
NIKR_SULSO	Hyperthermophiles	133	0.0000
NIKR_SYNAS	Mesophiles	137	0.0000
NIKR_THETN	Hyperthermophiles	154	0.0000
NOD11_BRAJA	Mesophiles	314	0.0478
NOD12_BRAJA	Mesophiles	321	0.0000
NOD21_BRAJA	Mesophiles	330	0.0788
NOD22_BRAJA	Mesophiles	312	0.0224
NODD1_RHILP	Mesophiles	314	0.0000
NODD2_RHILP	Mesophiles	318	0.0000
NODD3_RHILP	Mesophiles	302	0.0000
NODD_RHILE	Mesophiles	322	0.0124
NODD_RHILT	Mesophiles	318	0.0472
NODD_RHILV	Mesophiles	303	0.0000
NODW_BRAJA	Mesophiles	227	0.0485
NOLA_BRAJA	Mesophiles	237	0.1603
NORG_STAA3	Mesophiles	442	0.0090
NORG_STAAB	Mesophiles	442	0.0000
NORG_STAAM	Mesophiles	442	0.0090
NORR1_RALEH	Mesophiles	514	0.0700
NORR2_RALEH	Mesophiles	521	0.0653
NORR_AERHH	Mesophiles	508	0.0965
NORR_ECO24	Mesophiles	504	0.0179
NORR_ECO45	Mesophiles	504	0.0099
NORR_ECO57	Mesophiles	504	0.0079
NORR_ECO7I	Mesophiles	504	0.0278
NORR_ECO8A	Mesophiles	504	0.0099
NORR_ECODH	Mesophiles	504	0.0079
NORR_ECOHS	Mesophiles	504	0.0099
NORR_ECOL5	Mesophiles	504	0.0099
NORR_ECOL6	Mesophiles	504	0.0079
NORR_ECOLC	Mesophiles	504	0.0179
NORR_ECOLU	Mesophiles	504	0.0099
NORR_ECOSE	Mesophiles	504	0.0099
NORR_ECOSM	Mesophiles	504	0.0079
NORR_KLEP3	Mesophiles	516	0.0484
NORR_KLEP7	Mesophiles	516	0.0523
NORR_SALTY	Mesophiles	506	0.0059
NORR_SHIBS	Mesophiles	504	0.0179
NORR_SHIDS	Mesophiles	504	0.0099
NORR_SHIF8	Mesophiles	504	0.0099
NORR_SHISS	Mesophiles	504	0.0179
NORR_VIBVU	Mesophiles	510	0.0333
NORR_VIBVY	Mesophiles	510	0.0314

NREC_STAA1	Mesophiles	217	0.0046
NREC_STAAB	Mesophiles	217	0.0046
NREC_STAEQ	Mesophiles	218	0.0046
NREC_STAES	Mesophiles	218	0.0046
NREC_STAHJ	Mesophiles	217	0.0000
NSRR_ECO24	Mesophiles	141	0.0000
NSRR_ECO45	Mesophiles	141	0.0000
NSRR_ECO5E	Mesophiles	141	0.0000
NSRR_KLEP3	Mesophiles	141	0.0000
NSRR_PHOPR	Psychrophiles	144	0.0000
NSRR_SALTY	Mesophiles	141	0.0000
NSRR_SHIB3	Mesophiles	141	0.0000
NSRR_VIBHB	Mesophiles	141	0.0000
NSRR_VIBPA	Mesophiles	141	0.0000
NSRR_VIBVU	Mesophiles	141	0.0000
NSRR_YERE8	Mesophiles	141	0.0000
NSRR_YERPA	Mesophiles	141	0.0000
NTDR_BACSU	Mesophiles	329	0.0000
NTRC_ECO57	Mesophiles	469	0.0917
NTRC_KLEPN	Mesophiles	469	0.1322
NTRC_SALTY	Mesophiles	469	0.1279
NUSA_BACSU	Mesophiles	371	0.1321
NUSA_COXBU	Mesophiles	503	0.0775
NUSA_ECO57	Mesophiles	495	0.0283
NUSA_HAEIN	Mesophiles	495	0.0121
NUSA_HELPJ	Mesophiles	395	0.0380
NUSA_HELPY	Mesophiles	395	0.0304
NUSA_MYCBO	Mesophiles	347	0.1527
NUSA_MYCGE	Mesophiles	531	0.1620
NUSA_MYCLE	Mesophiles	347	0.0865
NUSA_MYCPN	Mesophiles	540	0.2500
NUSA_SALTY	Mesophiles	500	0.0200
NUSA_THET8	Thermophiles	387	0.1447
OHRR_BACSU	Mesophiles	147	0.0272
OPRR_PSEAE	Mesophiles	306	0.0098
ORUR_PSEAE	Mesophiles	339	0.0088
OXYR_ECO57	Mesophiles	305	0.0033
OXYR_HAEIN	Mesophiles	301	0.0066
OXYR_MYCAV	Mesophiles	311	0.0032
OXYR_MYCLE	Mesophiles	311	0.0064
P13_MYCMY	Mesophiles	113	0.0000
PAPX_ECOLX	Mesophiles	164	0.0061
PCF_BACSU	Mesophiles	169	0.1302
PCHR_PSEAE	Mesophiles	296	0.0878
PDHR_ECO57	Mesophiles	254	0.0984
PDHR_SALTY	Mesophiles	254	0.0984
PDXR_CORGL	Mesophiles	453	0.2009
PEPR_LACDL	Mesophiles	333	0.0060
PERA_ECO27	Mesophiles	274	0.0292
PERR_BACSU	Mesophiles	145	0.0000

PERR_ECOLI	Mesophiles	297	0.0000
PERR_STAA3	Mesophiles	148	0.0000
PERR_STAAM	Mesophiles	148	0.0000
PERR_STAEQ	Mesophiles	150	0.0000
PERR_STAHJ	Mesophiles	150	0.0000
PGTA_SALTY	Mesophiles	415	0.0024
PHNF_ECOLI	Mesophiles	241	0.0332
PHNF_MYCS2	Mesophiles	244	0.0123
PHNR_SALTY	Mesophiles	239	0.0126
PHNXW_CLOD6	Mesophiles	636	0.0126
PHYR_METEX	Mesophiles	267	0.0150
PHZR_PSEFL	Mesophiles	244	0.0000
PILR_PSEAE	Mesophiles	445	0.0629
PKSA_BACSU	Mesophiles	205	0.0829
POCR_SALTY	Mesophiles	303	0.0825
PRFA_LISMO	Mesophiles	237	0.0000
PRPR_ECOLI	Mesophiles	528	0.0284
PRPR_SALTY	Mesophiles	541	0.0591
PRSX_ECOL6	Mesophiles	166	0.0422
PSPF_ECOLI	Mesophiles	325	0.0185
PTSJ_SALTY	Mesophiles	430	0.1070
PTXR_PSEAE	Mesophiles	312	0.0000
PURR_ACTSZ	Mesophiles	336	0.0119
PURR_ECO24	Mesophiles	341	0.0411
PURR_ECO45	Mesophiles	341	0.0381
PURR_ECO57	Mesophiles	341	0.0411
PURR_ECOL6	Mesophiles	341	0.0381
PURR_ENTS8	Mesophiles	341	0.0264
PURR_HAEDU	Mesophiles	339	0.0059
PURR_HAEI8	Mesophiles	336	0.0060
PURR_HAEIG	Mesophiles	336	0.0060
PURR_HAEIN	Mesophiles	336	0.0000
PURR_HAES1	Mesophiles	333	0.0000
PURR_HAES2	Mesophiles	333	0.0000
PURR_KLEP3	Mesophiles	341	0.0264
PURR_KLEP7	Mesophiles	341	0.0323
PURR_MANSM	Mesophiles	334	0.0060
PURR_PASMU	Mesophiles	334	0.0000
PURR_PHOPR	Psychrophiles	334	0.0060
PURR_SALTY	Mesophiles	341	0.0381
PURR_SHIDS	Mesophiles	341	0.0411
PURR_SHISS	Mesophiles	341	0.0411
PURR_VIBC3	Mesophiles	336	0.0089
PURR_VIBHB	Mesophiles	334	0.0000
PURR_VIBPA	Mesophiles	334	0.0000
PURR_VIBVU	Mesophiles	334	0.0000
PURR_YERE8	Mesophiles	341	0.0440
PURR_YERPA	Mesophiles	341	0.0557
PURR_YERPE	Mesophiles	341	0.0440
QACR_STAAM	Mesophiles	188	0.0000

RACA_GEOKA	Thermophiles	172	0.3430
RAFR_ECOLX	Mesophiles	336	0.0506
RAFR_PEDPE	Mesophiles	277	0.0000
RAMA_KLEPN	Mesophiles	113	0.0973
RBSR_BACSU	Mesophiles	326	0.0215
RBSR_ECO57	Mesophiles	330	0.0303
RBSR_HAEIN	Mesophiles	332	0.0000
RBSR_LACLA	Mesophiles	327	0.0061
RBSR_PASMU	Mesophiles	337	0.0000
RCSA_ECO57	Mesophiles	207	0.0097
RCSA_SALTY	Mesophiles	207	0.0097
RCSB_ECO57	Mesophiles	216	0.0046
RCSB_SALTY	Mesophiles	216	0.0093
REG1_PYRAB	Hyperthermophiles	155	0.0000
REG1_PYRFU	Hyperthermophiles	155	0.0000
REG1_PYRHO	Hyperthermophiles	155	0.0000
REG2_PYRAB	Hyperthermophiles	148	0.0000
REG2_PYRFU	Hyperthermophiles	148	0.0000
REG2_PYRHO	Hyperthermophiles	148	0.0000
REG3_PYRAB	Hyperthermophiles	158	0.0000
REG3_PYRFU	Hyperthermophiles	148	0.0000
REG3_PYRHO	Hyperthermophiles	158	0.0000
REG4_PYRAB	Hyperthermophiles	162	0.0000
REG4_PYRFU	Hyperthermophiles	162	0.0000
REG4_PYRHO	Hyperthermophiles	162	0.0000
REG5_PYRHO	Hyperthermophiles	159	0.0000
REG6_PYRAB	Hyperthermophiles	151	0.0132
REG6_PYRFU	Hyperthermophiles	151	0.0000
REG6_PYRHO	Hyperthermophiles	151	0.0132
REG7_PYRAB	Hyperthermophiles	141	0.0000
REG7_PYRFU	Hyperthermophiles	141	0.0000
REG7_PYRHO	Hyperthermophiles	141	0.0000
REG8_PYRAB	Hyperthermophiles	147	0.0000
REG8_PYRFU	Hyperthermophiles	148	0.0000
REG8_PYRHO	Hyperthermophiles	148	0.0000
REG9_PYRAB	Hyperthermophiles	150	0.0000
REG9_PYRFU	Hyperthermophiles	150	0.0000
REG9_PYRHO	Hyperthermophiles	150	0.0000
REGA_CLOAB	Mesophiles	334	0.0000
REMA_BACSU	Mesophiles	146	0.0000
REMA_STAAU	Mesophiles	158	0.0000
REMA_STAEP	Mesophiles	162	0.0000
REX1_ENTFA	Mesophiles	216	0.0093
REX1_THEMA	Hyperthermophiles	208	0.0000
REX2_ENTFA	Mesophiles	215	0.0140
REX2_THEMA	Hyperthermophiles	204	0.0000
REX_BACC0	Mesophiles	209	0.0048
REX_BACC1	Mesophiles	209	0.0048
REX_BACC2	Mesophiles	209	0.0048
REX_BACC7	Mesophiles	209	0.0048

REX_BACCN	Mesophiles	209	0.0048
REX_BACCZ	Mesophiles	209	0.0048
REX_BACFN	Mesophiles	220	0.0636
REX_BACSU	Mesophiles	215	0.0233
REX_CALS8	Thermophiles	219	0.0000
REX_CLOAB	Mesophiles	214	0.0000
REX_CLOB1	Mesophiles	210	0.0000
REX_CLOBA	Mesophiles	210	0.0000
REX_CLOBB	Mesophiles	210	0.0000
REX_CLOBM	Mesophiles	210	0.0000
REX_CLOD6	Mesophiles	210	0.0000
REX_CLOP1	Mesophiles	212	0.0000
REX_CLOPS	Mesophiles	212	0.0000
REX_CLOTE	Mesophiles	211	0.0000
REX_DEIRA	Mesophiles	234	0.0085
REX_GEOKA	Thermophiles	213	0.0282
REX_GEOTN	Thermophiles	213	0.0282
REX_LACAC	Mesophiles	211	0.0000
REX_LACBA	Mesophiles	212	0.0047
REX_LACGA	Mesophiles	214	0.0047
REX_LACLA	Mesophiles	216	0.0509
REX_LACLC	Mesophiles	216	0.0509
REX_LACLS	Mesophiles	216	0.0509
REX_LACPL	Mesophiles	225	0.0578
REX_LEUMM	Mesophiles	216	0.0185
REX_LISIN	Mesophiles	215	0.0093
REX_OCEIH	Mesophiles	209	0.0096
REX_OENOB	Mesophiles	221	0.0000
REX_PEDPA	Mesophiles	208	0.0000
REX_PETMO	Thermophiles	211	0.0095
REX_PORG3	Mesophiles	218	0.0000
REX_PORGI	Mesophiles	218	0.0000
REX_ROSCS	Thermophiles	220	0.0182
REX_RUBXD	Thermophiles	216	0.0231
REX_STAA1	Mesophiles	211	0.0000
REX_STAAB	Mesophiles	211	0.0000
REX_STAEQ	Mesophiles	211	0.0000
REX_STAES	Mesophiles	211	0.0000
REX_STAHJ	Mesophiles	212	0.0000
REX_STRA1	Mesophiles	210	0.0000
REX_STRA3	Mesophiles	212	0.0000
REX_STRAW	Mesophiles	252	0.1786
REX_STRCO	Mesophiles	258	0.2171
REX_STRMU	Mesophiles	213	0.0000
REX_STRP1	Mesophiles	214	0.0000
REX_STRP2	Mesophiles	213	0.0000
REX_STRP4	Mesophiles	213	0.0000
REX_STRPB	Mesophiles	214	0.0000
REX_STRPF	Mesophiles	214	0.0000
REX_STRSU	Mesophiles	178	0.0056

REX_THEP1	Hyperthermophiles	208	0.0048
REX_THEP3	Thermophiles	224	0.0000
REX_THET2	Thermophiles	211	0.0000
REX_THETN	Hyperthermophiles	224	0.0000
REX_TREDE	Mesophiles	210	0.0000
RFAY_XANCP	Mesophiles	400	0.0275
RHAR_ECO24	Mesophiles	282	0.0284
RHAR_ECO57	Mesophiles	282	0.0284
RHAR_ECOHS	Mesophiles	282	0.0284
RHAR_ECOK1	Mesophiles	282	0.0284
RHAR_ENTS8	Mesophiles	283	0.0353
RHAR_KLEP7	Mesophiles	281	0.0178
RHAR_MANSM	Mesophiles	276	0.0072
RHAR_SALTY	Mesophiles	282	0.0496
RHAR_SHIDS	Mesophiles	282	0.0284
RHAR_SHIF8	Mesophiles	282	0.0284
RHAR_YERPA	Mesophiles	290	0.0069
RHAS_ECO24	Mesophiles	278	0.0360
RHAS_ECO45	Mesophiles	278	0.0360
RHAS_ECO57	Mesophiles	278	0.0360
RHAS_ECO8A	Mesophiles	278	0.0360
RHAS_ECOL5	Mesophiles	278	0.0360
RHAS_ECOL6	Mesophiles	278	0.0360
RHAS_ECOSE	Mesophiles	278	0.0360
RHAS_ECOSM	Mesophiles	278	0.0360
RHAS_KLEP3	Mesophiles	278	0.0000
RHAS_KLEP7	Mesophiles	278	0.0000
RHAS_MANSM	Mesophiles	268	0.0149
RHAS_SALTY	Mesophiles	278	0.0036
RHAS_SHIB3	Mesophiles	278	0.0360
RHAS_SHIDS	Mesophiles	278	0.0324
RHAS_SHIF8	Mesophiles	278	0.0252
RHAS_SHISS	Mesophiles	278	0.0360
RHAS_YERPA	Mesophiles	273	0.0037
RHAS_YERPG	Mesophiles	273	0.0037
RHAS_YERPP	Mesophiles	273	0.0037
RHIR_RHILV	Mesophiles	247	0.0000
RHLR_PSEAE	Mesophiles	241	0.0000
RIFK_ARCFU	Hyperthermophiles	233	0.0000
RIFK_CALMQ	Hyperthermophiles	230	0.0000
RIFK_METHJ	Mesophiles	221	0.0181
RIFK_METLZ	Mesophiles	222	0.0045
RIFK_METS5	Thermophiles	217	0.0000
RIFK_PYRAB	Hyperthermophiles	212	0.0000
RIFK_PYRFU	Hyperthermophiles	212	0.0000
RIFK_PYRHO	Hyperthermophiles	212	0.0000
RIFK_THEAC	Thermophiles	220	0.0000
RIFK_THEVO	Thermophiles	220	0.0000
RIPA_CORDI	Mesophiles	335	0.0925
RIPA_COREF	Mesophiles	332	0.0452

RIPA_CORGL	Mesophiles	331	0.0967
RNS_ECOLX	Mesophiles	265	0.0000
ROB_ECO57	Mesophiles	289	0.0727
ROCR_BACSU	Mesophiles	461	0.0868
ROT_STAA8	Mesophiles	166	0.0000
ROT_STAAC	Mesophiles	166	0.0000
ROT_STAAM	Mesophiles	166	0.0000
ROT_STAAR	Mesophiles	166	0.0000
RP28_BACCR	Mesophiles	237	0.0169
RP32_CAUCN	Mesophiles	295	0.1254
RP32_ECO57	Mesophiles	284	0.1232
RP32_HAEIN	Mesophiles	281	0.0214
RP32_PSEAE	Mesophiles	284	0.1620
RP32_VIBCH	Mesophiles	286	0.0350
RP32_VIBVU	Mesophiles	285	0.0351
RP32_ZYMMO	Mesophiles	302	0.1523
RP35_BACCR	Mesophiles	239	0.0000
RP54_BACSU	Mesophiles	436	0.1216
RP54_BRAJA	Mesophiles	484	0.1736
RP54_CAUCR	Mesophiles	497	0.1911
RP54_ECOLI	Mesophiles	477	0.1761
RP54_HELPJ	Mesophiles	414	0.0024
RP54_HELPY	Mesophiles	414	0.0000
RP54_PSEAE	Mesophiles	497	0.2254
RP54_RALEH	Mesophiles	73	0.1233
RP54_RHOSH	Mesophiles	434	0.0691
RP54_SALTY	Mesophiles	477	0.1908
RP55_BRAJA	Mesophiles	537	0.2980
RPIR_ECOL6	Mesophiles	296	0.0000
RPOD_BACSU	Mesophiles	371	0.2075
RPOD_CAUCR	Mesophiles	652	0.2408
RPOD_CHLMU	Mesophiles	571	0.1208
RPOD_CHLTR	Mesophiles	571	0.1068
RPOD_CLOAB	Mesophiles	378	0.1190
RPOD_COXBU	Mesophiles	698	0.3209
RPOD_ECOLI	Mesophiles	613	0.2480
RPOD_ENTFA	Mesophiles	368	0.1902
RPOD_HAEIN	Mesophiles	629	0.3402
RPOD_HELPJ	Mesophiles	681	0.1395
RPOD_HELPY	Mesophiles	671	0.1162
RPOD_LACLA	Mesophiles	386	0.0881
RPOD_LACLC	Mesophiles	364	0.0742
RPOD_LEPIC	Mesophiles	585	0.0615
RPOD_LISIN	Mesophiles	374	0.2380
RPOD_LISMO	Mesophiles	374	0.2326
RPOD_MYCBO	Mesophiles	528	0.4527
RPOD_MYCGE	Mesophiles	497	0.2797
RPOD_MYCPN	Mesophiles	499	0.3126
RPOD_MYXXA	Mesophiles	708	0.3319
RPOD_NEIGO	Mesophiles	642	0.2321

RPOD_PSEAE	Mesophiles	617	0.3323
RPOD_PSEFL	Mesophiles	615	0.3268
RPOD_SALTY	Mesophiles	615	0.2813
RPOD_STAA8	Mesophiles	368	0.2283
RPOD_STAAN	Mesophiles	368	0.2337
RPOD_STAEQ	Mesophiles	368	0.2690
RPOD_STRMU	Mesophiles	371	0.1348
RPOD_STRPN	Mesophiles	369	0.1599
RPOD_THEMA	Hyperthermophiles	399	0.2431
RPOD_XANAC	Mesophiles	625	0.2480
RPOD_XANCP	Mesophiles	624	0.2500
RPOD_XYLFA	Mesophiles	618	0.2799
RPOD_XYLFT	Mesophiles	618	0.2799
RPOE_ECO57	Mesophiles	191	0.0157
RPOE_HAEIN	Mesophiles	189	0.1111
RPOE_MYCBO	Mesophiles	216	0.2917
RPOE_SALTY	Mesophiles	191	0.0157
RPOE_STRAW	Mesophiles	179	0.1844
RPOE_STRCO	Mesophiles	177	0.1864
RPOF_STRCO	Mesophiles	287	0.2056
RPOM_ARCFU	Hyperthermophiles	103	0.0000
RPOM_SULAC	Thermophiles	111	0.0090
RPOS_COXBU	Mesophiles	352	0.2841
RPOS_ECOLI	Mesophiles	330	0.1303
RPOS_PSEAE	Mesophiles	334	0.2066
RPOS_SALTY	Mesophiles	330	0.1788
RPOS_SHIFL	Mesophiles	330	0.1394
RPOS_VIBCH	Mesophiles	335	0.0627
RPOS_YEREN	Mesophiles	331	0.2145
RPSB_BACSU	Mesophiles	262	0.0573
RPSB_CORDI	Mesophiles	329	0.1611
RPSB_MYXXA	Mesophiles	246	0.1301
RPSC_MYCBO	Mesophiles	185	0.0973
RPSC_MYXXA	Mesophiles	295	0.0576
RPSD_BACSU	Mesophiles	254	0.0276
RPSD_MYCBO	Mesophiles	212	0.0472
RPSE_BACSU	Mesophiles	239	0.0000
RPSE_CLOAB	Mesophiles	235	0.0000
RPSF_BACSU	Mesophiles	255	0.0196
RPSG_BACSU	Mesophiles	260	0.0154
RPSG_CLOAB	Mesophiles	257	0.0039
RPSH_BACSU	Mesophiles	218	0.0000
RPSH_PSEAE	Mesophiles	193	0.0622
RPSK_BACSU	Mesophiles	294	0.0204
RPSW_STRCO	Mesophiles	280	0.1357
RUTR_ECO57	Mesophiles	212	0.0094
RUTR_ECOL6	Mesophiles	212	0.0094
SACR_LACLA	Mesophiles	318	0.0000
SARS_STAA1	Mesophiles	250	0.0160
SARS_STAAR	Mesophiles	250	0.0160

SARU_STAA8	Mesophiles	247	0.0000
SARU_STAAC	Mesophiles	247	0.0000
SARV_STAA8	Mesophiles	116	0.0000
SARZ_STAA3	Mesophiles	148	0.0000
SARZ_STAAB	Mesophiles	148	0.0000
SARZ_STAAR	Mesophiles	148	0.0068
SARZ_STAEQ	Mesophiles	148	0.0068
SARZ_STAHJ	Mesophiles	147	0.0000
SCRR_KLEPN	Mesophiles	334	0.0180
SCRR_PEDPE	Mesophiles	326	0.0215
SCRR_SALTY	Mesophiles	334	0.0180
SCRR_STRMU	Mesophiles	320	0.0094
SDIA_ECOLI	Mesophiles	240	0.0125
SDPR_BACSU	Mesophiles	90	0.0000
SGCR_ECOLI	Mesophiles	260	0.0077
SIGK_MYCBO	Mesophiles	187	0.0107
SIGK_MYCUA	Mesophiles	187	0.0749
SIGK_MYCVP	Mesophiles	196	0.0204
SIGM_BACSU	Mesophiles	163	0.0000
SIGO_BACSU	Mesophiles	176	0.2784
SIGS_STAA1	Mesophiles	156	0.0000
SIGS_STAA3	Mesophiles	156	0.0000
SIGS_STAA8	Mesophiles	156	0.0000
SIGS_STAAB	Mesophiles	156	0.0000
SIGS_STAAR	Mesophiles	156	0.0000
SIGV_BACSU	Mesophiles	166	0.0000
SIGW_BACSU	Mesophiles	187	0.0000
SIGX_BACSU	Mesophiles	194	0.0825
SIGY_BACSU	Mesophiles	178	0.0506
SIGZ_BACSU	Mesophiles	176	0.0000
SINR_SALTY	Mesophiles	315	0.0000
SIRC_SALTY	Mesophiles	295	0.0000
SKGA_CAUCN	Mesophiles	255	0.0392
SLMA_ECO24	Mesophiles	198	0.0303
SLMA_ECOL5	Mesophiles	198	0.0303
SLMA_ENTS8	Mesophiles	198	0.0303
SLMA_HAEDU	Mesophiles	202	0.0149
SLMA_HAEI8	Mesophiles	218	0.0505
SLMA_IDILO	Mesophiles	196	0.0255
SLMA_KLEP7	Mesophiles	198	0.0303
SLMA_MANSM	Mesophiles	216	0.1019
SLMA_PASMU	Mesophiles	199	0.0402
SLMA_PHOPR	Psychrophiles	196	0.0255
SLMA_SALTY	Mesophiles	198	0.0303
SLMA_SHEAM	Mesophiles	197	0.0000
SLMA_SHEDO	Mesophiles	197	0.0051
SLMA_SHEFN	Mesophiles	197	0.0051
SLMA_SHESH	Psychrophiles	197	0.0000
SLMA_VIBCH	Mesophiles	196	0.0051
SLMA_VIBHB	Mesophiles	196	0.0255

SLMA_VIBPA	Mesophiles	196	0.0204
SLMA_VIBVU	Mesophiles	196	0.0153
SLMA_YERE8	Mesophiles	198	0.0354
SLMA_YERPA	Mesophiles	198	0.0354
SLYA_ECO24	Mesophiles	144	0.0069
SLYA_ECO27	Mesophiles	144	0.0069
SLYA_SALTY	Mesophiles	144	0.0139
SLYA_YERE8	Mesophiles	143	0.0070
SLYA_YERPA	Mesophiles	143	0.0070
SLYA_YERPE	Mesophiles	143	0.0070
SOPA_ECO57	Mesophiles	388	0.0361
SORC_KLEPN	Mesophiles	315	0.0000
SOXR_CHRVO	Mesophiles	148	0.0000
SOXR_ECO57	Mesophiles	154	0.1104
SOXR_PSEAE	Mesophiles	156	0.1282
SOXR_SALTY	Mesophiles	152	0.0395
SOXS_ECO57	Mesophiles	107	0.0000
SOXS_SALTY	Mesophiles	107	0.0000
SP0A_BACCE	Mesophiles	150	0.0067
SP0A_BACCR	Mesophiles	264	0.0000
SP0A_BACSU	Mesophiles	267	0.0599
SP0A_CLOAB	Mesophiles	281	0.0142
SP0A_CLODI	Mesophiles	274	0.0474
SP0A_MOOTA	Thermophiles	256	0.0352
SP3D_BACSU	Mesophiles	93	0.0645
SRLR_ECOLI	Mesophiles	257	0.0156
SYFA_METKA	Hyperthermophiles	524	0.1718
SYFA_PYRAB	Hyperthermophiles	500	0.0020
SYFA_SULAC	Thermophiles	465	0.0000
TBP1_METAC	Mesophiles	183	0.0164
TBP1_METMA	Mesophiles	183	0.0000
TBP2_METAC	Mesophiles	185	0.0000
TBP2_METMA	Mesophiles	183	0.0164
TBP3_METAC	Mesophiles	185	0.0000
TBP3_METMA	Mesophiles	185	0.0000
TBP_AERPE	Hyperthermophiles	203	0.0000
TBP_ARCFU	Hyperthermophiles	183	0.0109
TBP_HALMA	Mesophiles	186	0.0269
TBP_META3	Mesophiles	181	0.0331
TBP_METKA	Hyperthermophiles	185	0.0054
TBP_METM6	Mesophiles	181	0.0000
TBP_METM7	Mesophiles	181	0.0000
TBP_METMP	Mesophiles	181	0.0000
TBP_METS3	Mesophiles	181	0.0000
TBP_METST	Mesophiles	183	0.0000
TBP_METTP	Thermophiles	183	0.0000
TBP_METVS	Mesophiles	181	0.0000
TBP_PICTO	Thermophiles	184	0.0000
TBP_PYRAB	Hyperthermophiles	191	0.0000
TBP_PYRAE	Hyperthermophiles	199	0.0302

TBP_PYRCJ	Hyperthermophiles	199	0.0804
TBP_PYRFU	Hyperthermophiles	191	0.0000
TBP_PYRHO	Hyperthermophiles	191	0.0000
TBP_PYRIL	Thermophiles	199	0.0402
TBP_SULAC	Thermophiles	197	0.0000
TBP_SULSO	Hyperthermophiles	198	0.0000
TBP_SULTO	Hyperthermophiles	198	0.0000
TBP_THEAC	Thermophiles	184	0.0000
TBP_THEVO	Thermophiles	184	0.0000
TCAR_STAAC	Mesophiles	151	0.0066
TCPN_VIBC3	Mesophiles	276	0.0000
TDCA_ECO57	Mesophiles	312	0.0000
TETC_ECOLX	Mesophiles	197	0.0305
TETD_ECOLX	Mesophiles	138	0.0000
TETR1_ECOLX	Mesophiles	216	0.1481
TETR2_ECOLX	Mesophiles	207	0.1014
TETR3_ECOLX	Mesophiles	219	0.1416
TETR4_ECOLX	Mesophiles	218	0.0826
TETR5_ECOLX	Mesophiles	211	0.0948
TETR8_PASMU	Mesophiles	207	0.1111
TF2B1_SULSO	Hyperthermophiles	309	0.0809
TF2B1_THEAC	Thermophiles	312	0.0865
TF2B1_THEVO	Thermophiles	312	0.0801
TF2B2_SULSO	Hyperthermophiles	293	0.0683
TF2B2_THEAC	Thermophiles	307	0.0749
TF2B2_THEVO	Thermophiles	313	0.0639
TF2B_AERPE	Hyperthermophiles	322	0.2919
TF2B_ARCFU	Hyperthermophiles	326	0.0123
TF2B_CALMQ	Hyperthermophiles	336	0.0982
TF2B_META3	Mesophiles	334	0.0000
TF2B_METAC	Mesophiles	337	0.0564
TF2B_METKA	Hyperthermophiles	307	0.1792
TF2B_METM5	Mesophiles	339	0.0118
TF2B_METM6	Mesophiles	339	0.0206
TF2B_METM7	Mesophiles	339	0.0354
TF2B_METMA	Mesophiles	337	0.0682
TF2B_METMJ	Mesophiles	337	0.0772
TF2B_METMP	Mesophiles	339	0.0265
TF2B_METS3	Mesophiles	310	0.0161
TF2B_METST	Mesophiles	311	0.0804
TF2B_METTP	Thermophiles	337	0.0534
TF2B_PYRAB	Hyperthermophiles	300	0.0200
TF2B_PYRAE	Hyperthermophiles	333	0.0541
TF2B_PYRAR	Hyperthermophiles	333	0.0180
TF2B_PYRFU	Hyperthermophiles	300	0.0200
TF2B_PYRHO	Hyperthermophiles	300	0.0200
TF2B_PYRIL	Thermophiles	333	0.0480
TF2B_SULAC	Thermophiles	307	0.0912
TF2B_SULTO	Hyperthermophiles	308	0.1623
TFDR_RALEJ	Mesophiles	295	0.0034

TFDS_RALEJ	Mesophiles	295	0.0034
TFDT_RALEJ	Mesophiles	228	0.0000
TIPA_STRCO	Mesophiles	253	0.2609
TNRA_BACSU	Mesophiles	110	0.1909
TRAJ8_ECOLX	Mesophiles	149	0.0000
TRER_BACSU	Mesophiles	238	0.0126
TRER_ECOLI	Mesophiles	315	0.0286
TRER_SALTY	Mesophiles	315	0.0825
TRPI_PSEAE	Mesophiles	295	0.0068
TRPO_PSEAE	Mesophiles	207	0.0242
TRPR_ACTSZ	Mesophiles	101	0.0594
TRPR_CHLCV	Mesophiles	101	0.0099
TRPR_CHLT2	Mesophiles	94	0.0000
TRPR_CHLTA	Mesophiles	94	0.0000
TRPR_ECO24	Mesophiles	108	0.0463
TRPR_ECO45	Mesophiles	108	0.0463
TRPR_ECO7I	Mesophiles	108	0.0463
TRPR_ECOSM	Mesophiles	108	0.0556
TRPR_ENTS8	Mesophiles	109	0.0826
TRPR_HAEI8	Mesophiles	101	0.0495
TRPR_HAES1	Mesophiles	106	0.0849
TRPR_HAES2	Mesophiles	106	0.0849
TRPR_KLEP3	Mesophiles	109	0.0459
TRPR_KLEP7	Mesophiles	109	0.0459
TRPR_MANSM	Mesophiles	101	0.1287
TRPR_PASMU	Mesophiles	102	0.1078
TRPR_SALTY	Mesophiles	108	0.0463
TRPR_VIBCH	Mesophiles	103	0.3301
TRPR_VIBHB	Mesophiles	100	0.3400
TRPR_VIBPA	Mesophiles	103	0.1359
TRPR_VIBVU	Mesophiles	102	0.2353
TRPR_XYLF2	Mesophiles	92	0.0000
TRPR_XYLFA	Mesophiles	92	0.0000
TRPR_YERE8	Mesophiles	110	0.0545
TRPR_YERPE	Mesophiles	125	0.2480
TTDR_ECO57	Mesophiles	310	0.0000
TTDR_ECOL5	Mesophiles	310	0.0000
TTDR_ECOL6	Mesophiles	310	0.0000
TTDR_ECOLI	Mesophiles	310	0.0000
UHPA_ECO57	Mesophiles	196	0.0255
UHPA_SALTY	Mesophiles	196	0.0204
UIDR_ECO57	Mesophiles	196	0.0663
ULAR_ECO24	Mesophiles	251	0.1514
ULAR_ECO7I	Mesophiles	251	0.1235
ULAR_SALTY	Mesophiles	251	0.1474
ULAR_SHIDS	Mesophiles	251	0.1554
ULAR_SHIF8	Mesophiles	251	0.1275
ULAR_SHISS	Mesophiles	251	0.1554
URER_ECOLX	Mesophiles	296	0.0068
UVRC_BACSU	Mesophiles	598	0.0033

UVRY_ECO57	Mesophiles	218	0.0275
UVRY_ECOLI	Mesophiles	218	0.0275
UXUR_ECOLI	Mesophiles	257	0.1595
UXUR_HAEIN	Mesophiles	266	0.0000
VFR_PSEAE	Mesophiles	214	0.0093
VIRF_SHIDY	Mesophiles	262	0.0000
VIRF_YERE8	Mesophiles	271	0.0000
VIRF_YEREN	Mesophiles	271	0.0000
VIRS_MYCTU	Mesophiles	340	0.0147
VJBR_BRUA2	Mesophiles	259	0.0000
VJBR_BRUO2	Mesophiles	259	0.0000
VQSM_PSEAE	Mesophiles	325	0.0031
VRAR_STAA1	Mesophiles	209	0.0000
VRAR_STAAC	Mesophiles	209	0.0000
VRAR_STAEQ	Mesophiles	209	0.0000
VRAR_STAHJ	Mesophiles	209	0.0000
VRPR_SALTY	Mesophiles	297	0.0000
XAPR_ECOLI	Mesophiles	294	0.0000
XYLR_ECO57	Mesophiles	392	0.0000
XYLR_HAEIN	Mesophiles	387	0.0155
Y043_MYCTU	Mesophiles	244	0.0205
Y069_CHLTR	Mesophiles	451	0.0488
Y077_STAAW	Mesophiles	745	0.0188
Y084_STAAC	Mesophiles	745	0.0188
Y086_METMP	Mesophiles	149	0.0872
Y089_PASMU	Mesophiles	67	0.0896
Y097_STAAN	Mesophiles	745	0.0188
Y097_STAAR	Mesophiles	191	0.0366
Y1005_METKA	Hyperthermophiles	325	0.1169
Y1009_HAEIN	Mesophiles	256	0.0352
Y1011_ZYMMO	Mesophiles	61	0.1148
Y1018_STRA3	Mesophiles	110	0.0000
Y1018_STRPC	Mesophiles	113	0.0000
Y101_MYCGE	Mesophiles	222	0.0000
Y103_KLEP7	Mesophiles	64	0.3125
Y1047_YERPG	Mesophiles	68	0.3529
Y1052_HAEIN	Mesophiles	298	0.0201
Y1056_HAEI8	Mesophiles	68	0.0735
Y1061_STRMU	Mesophiles	110	0.0000
Y1069_LACLM	Mesophiles	111	0.0000
Y1079_STAAN	Mesophiles	110	0.0091
Y107_STAAR	Mesophiles	745	0.0188
Y1098_METVS	Mesophiles	142	0.0352
Y1102_STAES	Mesophiles	255	0.0157
Y1122_STRGC	Mesophiles	110	0.0000
Y1129_HAEDU	Mesophiles	62	0.0000
Y1143_METST	Mesophiles	350	0.1600
Y1143_STRP2	Mesophiles	110	0.0000
Y1182_STRP4	Mesophiles	110	0.0000
Y1195_CLOBA	Mesophiles	109	0.0000

Y1195_GEOKA	Thermophiles	110	0.0000
Y1212_STAAR	Mesophiles	110	0.0000
Y1218_GEOSL	Mesophiles	61	0.3279
Y1244_CLOBB	Mesophiles	109	0.0000
Y1247_CLOTE	Mesophiles	117	0.0000
Y1255_MYCTU	Mesophiles	202	0.0545
Y1260_BRAJA	Mesophiles	60	0.2000
Y1266_PYRAB	Hyperthermophiles	110	0.0182
Y1268_SULTO	Hyperthermophiles	299	0.0000
Y1277_CHLTE	Thermophiles	197	0.3452
Y1278_THEP3	Thermophiles	117	0.0000
Y1288_STRPN	Mesophiles	110	0.0000
Y1298_BACC2	Mesophiles	110	0.0000
Y1300_PYRAB	Hyperthermophiles	145	0.0000
Y1344_SULAC	Thermophiles	305	0.0000
Y1353_MYCTU	Mesophiles	261	0.2759
Y1363_THEAC	Thermophiles	312	0.0000
Y1364_HAEIN	Mesophiles	288	0.0174
Y1378_NEIMB	Mesophiles	148	0.0068
Y1394_FUSNN	Mesophiles	103	0.0000
Y1395_MYCTU	Mesophiles	344	0.0116
Y142_AERPE	Hyperthermophiles	180	0.0389
Y1435_PYRAB	Hyperthermophiles	333	0.0390
Y143_HAEIN	Mesophiles	288	0.0069
Y1459_HAEIN	Mesophiles	194	0.0155
Y1463_THETN	Hyperthermophiles	117	0.0085
Y1514_METVS	Mesophiles	327	0.0183
Y1530_OCEIH	Mesophiles	105	0.0000
Y1556_MYCTU	Mesophiles	202	0.0743
Y1603_LACC3	Mesophiles	113	0.0000
Y160_METLZ	Mesophiles	317	0.1230
Y1619_VIBVU	Mesophiles	64	0.3906
Y161_STAAB	Mesophiles	252	0.0000
Y1627_PYRAE	Hyperthermophiles	250	0.0080
Y1634_LACPL	Mesophiles	115	0.0000
Y165_STAES	Mesophiles	251	0.0000
Y1672_NEIG1	Mesophiles	69	0.1739
Y1673_BRUME	Mesophiles	57	0.3333
Y1678_STAHJ	Mesophiles	110	0.0000
Y1686_CLOPS	Mesophiles	112	0.0000
Y1701_ENTFA	Mesophiles	112	0.0000
Y1702_METM7	Mesophiles	326	0.0460
Y1714_CLOPE	Mesophiles	112	0.0000
Y1731_HYPNA	Mesophiles	73	0.3973
Y1753_CLOAB	Mesophiles	116	0.0000
Y1758_BACCZ	Mesophiles	147	0.0612
Y1787_ARCFU	Hyperthermophiles	309	0.0000
Y1802_LISMO	Mesophiles	110	0.0000
Y1808_PYRHO	Hyperthermophiles	315	0.0032
Y1811_METBU	Mesophiles	323	0.0557

Y1813_NEIM0	Mesophiles	69	0.0290
Y1816_MYCTU	Mesophiles	234	0.0000
Y1827_CLOBM	Mesophiles	110	0.0000
Y1828_MYCTU	Mesophiles	247	0.0000
Y1829_LISMF	Mesophiles	110	0.0000
Y1830_MYCTU	Mesophiles	225	0.3156
Y1842_NEIMF	Mesophiles	69	0.1594
Y184_STAA8	Mesophiles	252	0.0000
Y1851_PYRFU	Hyperthermophiles	315	0.0159
Y186_HAEIN	Mesophiles	135	0.0000
Y189_CLOPE	Mesophiles	279	0.0036
Y1916_LISIN	Mesophiles	110	0.0000
Y191_CLOAB	Mesophiles	283	0.0177
Y1936_BACCR	Mesophiles	147	0.0612
Y1968_CLOP1	Mesophiles	112	0.0000
Y1985_MYCTU	Mesophiles	303	0.0528
Y199_HALMA	Mesophiles	159	0.0881
Y2019_BACC1	Mesophiles	147	0.0748
Y2057_DESRM	Mesophiles	117	0.0085
Y2090_METCA	Thermophiles	73	0.3562
Y210_METM6	Mesophiles	327	0.0306
Y211_THETN	Hyperthermophiles	282	0.0000
Y213_SINMW	Mesophiles	70	0.3857
Y2140_MYCLE	Mesophiles	143	0.0769
Y214_STAAR	Mesophiles	252	0.0000
Y2158_NEIMA	Mesophiles	69	0.1594
Y215_STAAN	Mesophiles	252	0.0000
Y2190_CLOBK	Mesophiles	110	0.0000
Y224_HAEIN	Mesophiles	168	0.0000
Y2250_MYCTU	Mesophiles	189	0.0159
Y2274_MYCBO	Mesophiles	189	0.0159
Y2282_MYCTU	Mesophiles	312	0.0641
Y2310_MYCTU	Mesophiles	114	0.0877
Y2340_CAUCR	Mesophiles	57	0.3509
Y2347_METMJ	Mesophiles	304	0.0329
Y2364_STAAN	Mesophiles	185	0.0162
Y2365_THIDA	Mesophiles	69	0.3333
Y2383_VIBCH	Mesophiles	286	0.0420
Y239_MYCPN	Mesophiles	222	0.0000
Y2406_STAEQ	Mesophiles	251	0.0080
Y2429_VIBCH	Mesophiles	65	0.3846
Y2452_STAAB	Mesophiles	185	0.0000
Y2498_BACCN	Mesophiles	110	0.0000
Y2506_CLOBL	Mesophiles	110	0.0000
Y2515_STAA3	Mesophiles	185	0.0162
Y2519_HALMA	Mesophiles	322	0.2702
Y2529_VIBPA	Mesophiles	64	0.3750
Y2548_METHJ	Mesophiles	306	0.0588
Y2658_STAAR	Mesophiles	185	0.0162
Y2784_VIBVY	Mesophiles	64	0.3906

Y280_BRUA2	Mesophiles	57	0.2807
Y2819_SYNFM	Mesophiles	156	0.1154
Y2887_MYCTU	Mesophiles	139	0.0935
Y2912_MYCTU	Mesophiles	195	0.0256
Y293_HAEIN	Mesophiles	128	0.0000
Y294_THEVO	Thermophiles	312	0.0000
Y300_SHEWM	Mesophiles	70	0.0000
Y3073_RHOS4	Mesophiles	456	0.1360
Y3206_PHOPR	Psychrophiles	76	0.3684
Y3238_ENTS8	Mesophiles	67	0.3582
Y326_THEMA	Hyperthermophiles	280	0.0000
Y329_MYCPN	Mesophiles	158	0.0000
Y330_NEIMB	Mesophiles	69	0.1594
Y3394_SHEDO	Mesophiles	74	0.0000
Y3405_MYCTU	Mesophiles	188	0.0957
Y341_CHLMU	Mesophiles	447	0.0224
Y3524_METAC	Mesophiles	328	0.0579
Y358_MANSM	Mesophiles	72	0.1667
Y364_SHEAM	Mesophiles	71	0.0000
Y367_PYRAB	Hyperthermophiles	316	0.0032
Y377_MYCTU	Mesophiles	321	0.0000
Y377_PSEHT	Psychrophiles	76	0.0658
Y379_HAEIN	Mesophiles	150	0.0000
Y3823_CHRVO	Mesophiles	63	0.2857
Y3874_AERHH	Mesophiles	64	0.3125
Y3946_HALS3	Thermophiles	320	0.1906
Y4158_STRCO	Mesophiles	340	0.0412
Y4167_CLOAB	Mesophiles	184	0.0000
Y420_SHESH	Psychrophiles	70	0.0000
Y424_MYCPN	Mesophiles	102	0.0098
Y428_MYCGE	Mesophiles	171	0.0000
Y444_METMA	Mesophiles	327	0.0642
Y4452_COLP3	Psychrophiles	78	0.0000
Y447_IDILO	Mesophiles	64	0.3125
Y4530_PSEAE	Mesophiles	66	0.2121
Y453_METS3	Mesophiles	313	0.0224
Y4628_STRCO	Mesophiles	269	0.2937
Y4637_KLEP3	Mesophiles	64	0.3125
Y4778_PSEAE	Mesophiles	132	0.0076
Y480_MYCCT	Mesophiles	113	0.0000
Y4824_PSEPF	Mesophiles	66	0.3030
Y494_MYCTU	Mesophiles	242	0.1446
Y5291_PSEF5	Mesophiles	67	0.3134
Y529_ARCFU	Hyperthermophiles	74	0.0135
Y535_PYRFU	Hyperthermophiles	142	0.0000
Y557_PICTO	Thermophiles	311	0.0000
Y570_HAEIN	Mesophiles	205	0.0000
Y586_MYCTU	Mesophiles	240	0.0667
Y603_AQUAE	Hyperthermophiles	272	0.0000
Y614_PYRHO	Hyperthermophiles	332	0.0361

Y620_PYRFU	Hyperthermophiles	108	0.0278
Y626_MYCPN	Mesophiles	172	0.0000
Y678_METMP	Mesophiles	326	0.0337
Y683_YERE8	Mesophiles	68	0.3235
Y763_PYRHO	Hyperthermophiles	143	0.0000
Y767_MYCTU	Mesophiles	213	0.1174
Y771_CLOTH	Thermophiles	117	0.0000
Y775_HAEIN	Mesophiles	315	0.0000
Y778_AERPE	Hyperthermophiles	329	0.0213
Y802_STAEQ	Mesophiles	110	0.0000
Y803_PYRHO	Hyperthermophiles	110	0.0273
Y845_PEDPA	Mesophiles	113	0.0088
Y875_STRSY	Mesophiles	113	0.0000
Y880_MYCTU	Mesophiles	143	0.0559
Y888_STRT1	Thermophiles	110	0.0000
Y890_MYCTU	Mesophiles	882	0.0351
Y891_HAEIN	Mesophiles	64	0.0625
Y893_HAEIN	Mesophiles	187	0.0000
Y898_METM5	Mesophiles	326	0.0583
Y905_STRP6	Mesophiles	113	0.0000
Y911_STAES	Mesophiles	110	0.0000
Y914_MYCBO	Mesophiles	882	0.0283
Y942_SULSO	Hyperthermophiles	310	0.0032
Y949_LACBA	Mesophiles	113	0.0000
YACG_ECO24	Mesophiles	65	0.3231
YACG_ECOL6	Mesophiles	65	0.3077
YACG_SALTY	Mesophiles	63	0.3016
YAFC_ECOLI	Mesophiles	304	0.0099
YAHA_ECOLI	Mesophiles	362	0.0000
YAHB_ECOLI	Mesophiles	310	0.0161
YBAO_ECO57	Mesophiles	152	0.0000
YBBB_BACSU	Mesophiles	529	0.0000
YBBH_BACSU	Mesophiles	283	0.0071
YBCM_ECOLI	Mesophiles	265	0.0113
YBDO_ECOLI	Mesophiles	300	0.0000
YBEF_ECOLI	Mesophiles	197	0.0000
YBFI_BACSU	Mesophiles	275	0.0073
YBFP_BACSU	Mesophiles	295	0.0237
YBGA_BACSU	Mesophiles	235	0.0043
YBHD_ECOLI	Mesophiles	317	0.0000
YBIH_ECOLI	Mesophiles	223	0.0493
YBJK_ECOLI	Mesophiles	178	0.0337
YCAN_ECOLI	Mesophiles	302	0.0066
YCBG_BACSU	Mesophiles	233	0.0043
YCEK_BACSU	Mesophiles	100	0.0000
YCFQ_ECOLI	Mesophiles	210	0.0095
YCGE_BACSU	Mesophiles	154	0.0974
YCGE_ECOLI	Mesophiles	243	0.0123
YCGK_BACSU	Mesophiles	324	0.0062
YCIT_ECOLI	Mesophiles	249	0.0000

YCJW_ECOLI	Mesophiles	332	0.0271
YCJZ_ECOLI	Mesophiles	299	0.0033
YCNC_BACSU	Mesophiles	292	0.0068
YCNK_BACSU	Mesophiles	190	0.0000
YCXD_BACSU	Mesophiles	444	0.0203
YCZG_BACSU	Mesophiles	104	0.0577
YDCH_BACSU	Mesophiles	147	0.0136
YDCI_ECOLI	Mesophiles	307	0.0326
YDCR_ECOLI	Mesophiles	468	0.0000
YDEC_BACSU	Mesophiles	291	0.0000
YDEE_BACSU	Mesophiles	290	0.0517
YDEF_BACSU	Mesophiles	462	0.0065
YDEL_BACSU	Mesophiles	463	0.0086
YDEO_ECO57	Mesophiles	253	0.0198
YDEO_ECOL6	Mesophiles	253	0.0198
YDEO_ECOLI	Mesophiles	253	0.0198
YDEO_SHIFL	Mesophiles	253	0.0158
YDES_BACSU	Mesophiles	198	0.0707
YDFD_BACSU	Mesophiles	62	0.0000
YDFF_BACSU	Mesophiles	226	0.0000
YDFH_ECO57	Mesophiles	228	0.0482
YDFH_ECOL6	Mesophiles	228	0.0482
YDFI_BACSU	Mesophiles	213	0.0094
YDFL_BACSU	Mesophiles	270	0.0000
YDGC_BACSU	Mesophiles	195	0.0000
YDGG_BACSU	Mesophiles	152	0.0132
YDGJ_BACSU	Mesophiles	164	0.1037
YDHB_ECO57	Mesophiles	310	0.0484
YDHC_BACSU	Mesophiles	224	0.0402
YDHM_ECOL6	Mesophiles	199	0.0452
YDIP_ECOLI	Mesophiles	303	0.0000
YDJF_ECOLI	Mesophiles	252	0.0000
YEAM_ECOLI	Mesophiles	273	0.0586
YEAT_ECOLI	Mesophiles	307	0.0000
YEEY_ECOLI	Mesophiles	309	0.0000
YEGW_ECO57	Mesophiles	248	0.0000
YEIE_ECO57	Mesophiles	293	0.0000
YENR_YEREN	Mesophiles	244	0.0287
YERO_BACSU	Mesophiles	289	0.0138
YESN_BACSU	Mesophiles	368	0.0217
YESS_BACSU	Mesophiles	761	0.0118
YETL_BACSU	Mesophiles	167	0.0240
YEZE_BACSU	Mesophiles	194	0.0000
YFER_ECOLI	Mesophiles	308	0.0032
YFET_ECOLI	Mesophiles	285	0.0246
YFHH_ECOLI	Mesophiles	282	0.0390
YFIE_ECOLI	Mesophiles	293	0.0068
YFIF_BACSU	Mesophiles	314	0.0127
YFIK_BACSU	Mesophiles	220	0.0000
YFIR_BACSU	Mesophiles	205	0.0585

YFIV_BACSU	Mesophiles	160	0.0125
YFJR_ECOLI	Mesophiles	233	0.0000
YFMP_BACSU	Mesophiles	140	0.0500
YGAE_ECOLI	Mesophiles	220	0.0000
YGAV_ECOLI	Mesophiles	99	0.0505
YGBI_ECOLI	Mesophiles	265	0.0453
YGEK_ECO57	Mesophiles	210	0.0000
YGEK_ECOLI	Mesophiles	210	0.0000
YGEV_ECOLI	Mesophiles	592	0.0473
YGFI_ECOLI	Mesophiles	298	0.0101
YHAJ_ECO57	Mesophiles	298	0.0168
YHBI_BACSU	Mesophiles	154	0.0260
YHCF_BACSU	Mesophiles	121	0.0331
YHCZ_BACSU	Mesophiles	214	0.1121
YHDI_BACSU	Mesophiles	469	0.0362
YHGD_BACSU	Mesophiles	191	0.0000
YHJB_ECOLI	Mesophiles	200	0.0000
YHJC_ECOLI	Mesophiles	299	0.0000
YHJH_BACSU	Mesophiles	175	0.0171
YHTH1_STAAU	Mesophiles	745	0.0228
YIAU_ECOLI	Mesophiles	324	0.0401
YIDL_ECOLI	Mesophiles	307	0.0391
YIDP_ECOLI	Mesophiles	238	0.0000
YIDZ_ECO24	Mesophiles	319	0.0063
YIDZ_ECO57	Mesophiles	319	0.0063
YIDZ_ECODH	Mesophiles	319	0.0000
YIDZ_ECOL6	Mesophiles	319	0.0063
YIDZ_ECOLU	Mesophiles	319	0.0063
YIDZ_SALTY	Mesophiles	319	0.0063
YIDZ_SHIDS	Mesophiles	319	0.0063
YIEP_ECOLI	Mesophiles	230	0.0000
YIHL_ECO57	Mesophiles	236	0.1356
YIHW_ECOLI	Mesophiles	261	0.0651
YIJO_ECOLI	Mesophiles	283	0.0636
YISR_BACSU	Mesophiles	287	0.0000
YISV_BACSU	Mesophiles	484	0.0455
YJCO_BACSU	Mesophiles	153	0.0458
YJDC_ECOL6	Mesophiles	191	0.0000
YJGJ_ECOLI	Mesophiles	197	0.0660
YJIE_ECOLI	Mesophiles	123	0.0000
YJIR_ECOLI	Mesophiles	470	0.0723
YJJM_ECOLI	Mesophiles	304	0.0230
YJJQ_ECO57	Mesophiles	241	0.0249
YKGA_ECOLI	Mesophiles	239	0.0000
YKGD_ECOLI	Mesophiles	284	0.0035
YKOM_BACSU	Mesophiles	154	0.0130
YKUM_BACSU	Mesophiles	293	0.0000
YKVZ_BACSU	Mesophiles	321	0.0000
YLA3_LACAC	Mesophiles	124	0.0565
YLAC_BACSU	Mesophiles	173	0.0405

YLXM_BACSU	Mesophiles	110	0.0000
YMFC_BACSU	Mesophiles	241	0.0041
YMFN_ECOLI	Mesophiles	455	0.0593
YNEJ_ECOLI	Mesophiles	293	0.0000
YNFL_ECOLI	Mesophiles	297	0.0168
YOAU_BACSU	Mesophiles	290	0.0000
YOBQ_BACSU	Mesophiles	241	0.0000
YOBS_BACSU	Mesophiles	191	0.0000
YOBV_BACSU	Mesophiles	313	0.0000
YOFA_BACSU	Mesophiles	285	0.0632
YOFM_LACLA	Mesophiles	111	0.0000
YPB3_LACLA	Mesophiles	196	0.0000
YPDC_ECOLI	Mesophiles	285	0.0000
YPOP_BACSU	Mesophiles	141	0.0000
YQHC_ECOLI	Mesophiles	318	0.0220
YQIR_BACSU	Mesophiles	692	0.0289
YRAN_BACSU	Mesophiles	289	0.0000
YSMB_BACSU	Mesophiles	146	0.0000
YTDP_BACSU	Mesophiles	772	0.0155
YTLI_BACSU	Mesophiles	308	0.0487
YTRA_BACSU	Mesophiles	130	0.0231
YTTP_BACSU	Mesophiles	207	0.0000
YTZE_BACSU	Mesophiles	73	0.1096
YUAB_ECOLI	Mesophiles	353	0.0000
YULB_BACSU	Mesophiles	258	0.0194
YURK_BACSU	Mesophiles	242	0.0041
YUSO_BACSU	Mesophiles	155	0.1419
YUST_BACSU	Mesophiles	295	0.0000
YUXN_BACSU	Mesophiles	291	0.0000
YVAF_BACSU	Mesophiles	190	0.0105
YVBU_BACSU	Mesophiles	292	0.0034
YVDE_BACSU	Mesophiles	316	0.0000
YVDT_BACSU	Mesophiles	194	0.0000
YVFU_BACSU	Mesophiles	200	0.0000
YVKB_BACSU	Mesophiles	189	0.0053
YVMB_BACSU	Mesophiles	169	0.0769
YVNA_BACSU	Mesophiles	37	0.0000
YVOA_BACSU	Mesophiles	243	0.1481
YWAE_BACSU	Mesophiles	171	0.0994
YWBI_BACSU	Mesophiles	301	0.0000
YWCC_BACSU	Mesophiles	223	0.0000
YWHA_BACSU	Mesophiles	139	0.0791
YWOH_BACSU	Mesophiles	137	0.0000
YWQM_BACSU	Mesophiles	293	0.0034
YWRC_BACSU	Mesophiles	158	0.0000
YXAD_BACSU	Mesophiles	143	0.0000
YXAF_BACSU	Mesophiles	191	0.0576
YXBF_BACSU	Mesophiles	380	0.0105
YXJL_BACSU	Mesophiles	218	0.0092
YXJO_BACSU	Mesophiles	291	0.0172

YYAN_BACSU	Mesophiles	138	0.0000
YYBA_BACSU	Mesophiles	150	0.0200
YYBE_BACSU	Mesophiles	292	0.0000
YYDK_BACSU	Mesophiles	236	0.0000
ZNTR_ECO57	Mesophiles	141	0.0284
ZNTR_HAEIN	Mesophiles	135	0.0741
ZRAR_ECO57	Mesophiles	441	0.0113
ZRAR_ECOLI	Mesophiles	441	0.0113
ZRAR_SALTY	Mesophiles	441	0.0159
ZRP_ZYMMO	Mesophiles	153	0.0000
ZURR_LISIN	Mesophiles	141	0.0000
ZUR_BACSU	Mesophiles	145	0.0138
ZUR_ECOLI	Mesophiles	171	0.0468
